# Oxygen and glucose deprivation induces widespread alterations in mRNA translation within 20 minutes

**DOI:** 10.1186/s13059-015-0651-z

**Published:** 2015-05-06

**Authors:** Dmitry E Andreev, Patrick BF O’Connor, Alexander V Zhdanov, Ruslan I Dmitriev, Ivan N Shatsky, Dmitri B Papkovsky, Pavel V Baranov

**Affiliations:** School of Biochemistry and Cell Biology, Western Gateway Building, University College Cork, Cork, Ireland; Belozersky Institute of Physico-Chemical Biology, Lomonosov Moscow State University, Moscow, 119992 Russia; Institute of Biomedical Chemistry, Pogodinskaya street, Moscow, 119121 Russia

## Abstract

**Background:**

Oxygen and glucose metabolism play pivotal roles in many (patho)physiological conditions. In particular, oxygen and glucose deprivation (OGD) during ischemia and stroke results in extensive tissue injury and cell death.

**Results:**

Using time-resolved ribosome profiling, we assess gene expression levels in a neural cell line, PC12, during the first hour of OGD. The most substantial alterations are seen to occur within the first 20 minutes of OGD. While transcription of only 100 genes is significantly altered during one hour of OGD, the translation response affects approximately 3,000 genes. This response involves reprogramming of initiation and elongation rates, as well as the stringency of start codon recognition. Genes involved in oxidative phosphorylation are most affected. Detailed analysis of ribosome profiles reveals salient alterations of ribosome densities on individual mRNAs. The mRNA-specific alterations include increased translation of upstream open reading frames, site-specific ribosome pauses, and production of alternative protein isoforms with amino-terminal extensions. Detailed analysis of ribosomal profiles also reveals six mRNAs with translated ORFs occurring downstream of annotated coding regions and two examples of dual coding mRNAs, where two protein products are translated from the same long segment of mRNA, but in two different frames.

**Conclusions:**

These findings uncover novel regulatory mechanisms of translational response to OGD in mammalian cells that are different from the classical pathways such as hypoxia inducible factor (HIF) signaling, while also revealing sophisticated organization of protein coding information in certain genes.

**Electronic supplementary material:**

The online version of this article (doi:10.1186/s13059-015-0651-z) contains supplementary material, which is available to authorized users.

## Background

The World Health Organization reports ischemic heart disease and stroke as the leading causes of death in humans [1]. Both diseases are characterized by the interruption of blood flow and oxygen and glucose supply, which induce severe tissue damage. Metabolic stress, acidosis, energy crisis, cytosolic Na^+^ and Ca^2+^ accumulation, over-production of reactive oxygen and nitrogen species and mitochondrial impairment are the hallmarks of the ischemic cascade, which ultimately leads to cell death [2]. A growing knowledge of the mechanisms of this cascade provides opportunities for its modulation in order to counteract disease-related outcomes [3,4].

The response to oxygen and glucose deprivation (OGD) is known to involve hypoxia inducible factors (HIFs) and 5′ AMP-activated protein kinase (AMPK) signaling pathways. HIFs modulate gene expression at the transcriptional level via binding to HIF-responsive elements (HREs) in the promoter regions of the regulated genes [5-7]. Recently it was shown that in conditions of overall suppression of protein synthesis under hypoxia (achieved by eIF4E sequestration), EPAS1 (also known as HIF-2α) can activate translation of mRNAs containing specific RNA secondary structure (RNA hypoxia responsive element, rHRE) signals in their 3′ UTRs [8]. AMPK is an important sensor of energy starvation and adaptive response to ischemia in mammalian cells [9,10]. It affects gene expression via phosphorylation of different proteins, including elongation factor 2 (eEF2) *via* eEF2k [11], which leads to a general inhibition of protein synthesis.

Whereas HIF-1α levels increase within minutes upon hypoxia onset [12], and reach maximal values *in vivo* within 1 to 5 h in a tissue-specific manner [13], changes in the levels of HIF target proteins (which depend on sequential transcription, maturation and export of new mRNAs, followed by translation) lag behind HIF activation. In contrast, hypoxia-dependent changes in the translation or stability of the already existing mRNA pool may provide a quicker adjustment of gene expression - for example, via reduced hydroxylation of the components of the translational machinery [14] or AMPK activation [9,10]. We reasoned that, during OGD, changes in *de novo* protein production in mammalian cells may occur prior to HIF-mediated reprogramming of gene transcription. To investigate this, we exposed neural PC12 cells to 20, 40 and 60 minutes of OGD in a hypoxia workstation (Figure A1 in Additional file 1) and examined the dynamics of genome-wide changes in transcription and translation by means of ribosome profiling (ribo-seq). We also closely monitored oxygen levels, mitochondrial polarization and the phosphorylation status of key components of relevant metabolic pathways.

Analysis of ribosomal profiling data revealed approximately 3,000 genes affected at the level of translation. RNA levels of only slightly more than a hundred genes were affected during the same time. The major phase of the response took place within the first 20 minutes. The response involved alteration of general parameters of translation; we observed reduced stringency of start codon selection and increased accuracy of translation termination. Detailed investigations of individual mRNA profiles allowed us to identify over 60 upregulated upstream ORFs (uORFs), numerous site-specific OGD-induced ribosome pauses, synthesis of proteins with terminal extensions and a few translated ORFs located downstream of annotated coding regions. These results suggest that the early OGD-mediated reprogramming of gene expression precedes the canonical HIF-mediated response and it may significantly affect the latter, as well as cell fate in general.

## Results and discussion

### Energy stress, HIF signaling and pathways regulating translation under oxygen and glucose deprivation

**Figure 1:**
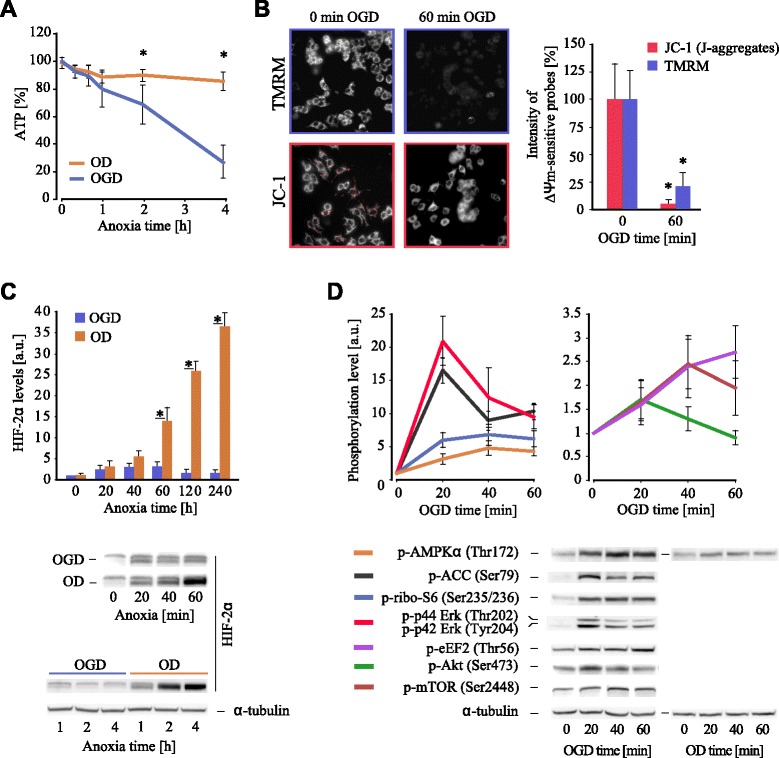
*In vitro* model of ischemia. **(A)** Analysis of cellular ATP during OGD. Reduction in ATP becomes significant after 2 h. In the presence of glucose, ATP levels remain unchanged. OD, oxygen deprivation. **(B)** Decreased intensities of TMRM and J-aggregated form of JC-1 (red) probes under OGD indicate ΔΨm depolarization. **(C)** Dissimilar to OD, only a minor transient elevation in Epas1 (also known as HIF-2α) levels is seen under OGD (arbitrary units (a.u.)). **(D)** Characteristic OGD-induced changes in phosphorylation of the proteins involved in regulation of translation. A minor increase in phosphorylated AMPKα under OD suggests a smaller effect of this condition on AMPK signaling. Scale bar is 50 μm. Asterisks demonstrate significant difference. Error bars show standard deviation (A,C,D) or a range in TMRM and JC-1 intensities (B), calculated for 40 to 50 randomly selected cells.

After 1 h of OGD cellular ATP levels decreased to approximately 80% of control conditions and continued to decrease further almost linearly with time (Figure 1A). In line with this, the mitochondrial membrane potential (ΔΨm) probes TMRM (Tetramethylrhodamine, methyl ester) and JC-1 showed a progressive depolarization of mitochondria in cells exposed to OGD (Figure 1B; Figure A2A,B in Additional file 1). As expected, these effects were not observed when glucose was available: ATP and ΔΨ_m_ levels decreased only slightly due to the continued ATP generation via glycolysis and activity of F1Fo ATP synthase working in reverse mode to maintain mitochondrial polarization. Under energy stress imposed on the cells, only a minor transient elevation in Epas1 (also known as HIF-2α) was observed along with a substantial increase in AMPK phosphorylation (Thr172) during the course of OGD, contrasting with oxygen deprivation (OD) conditions (Figure 1C,D; Figure A2C in Additional file 1) [15].

### Oxygen and glucose deprivation alters translation of approximately 3,000 mRNAs, while affecting RNA levels of only about 100 genes

**Figure 2:**
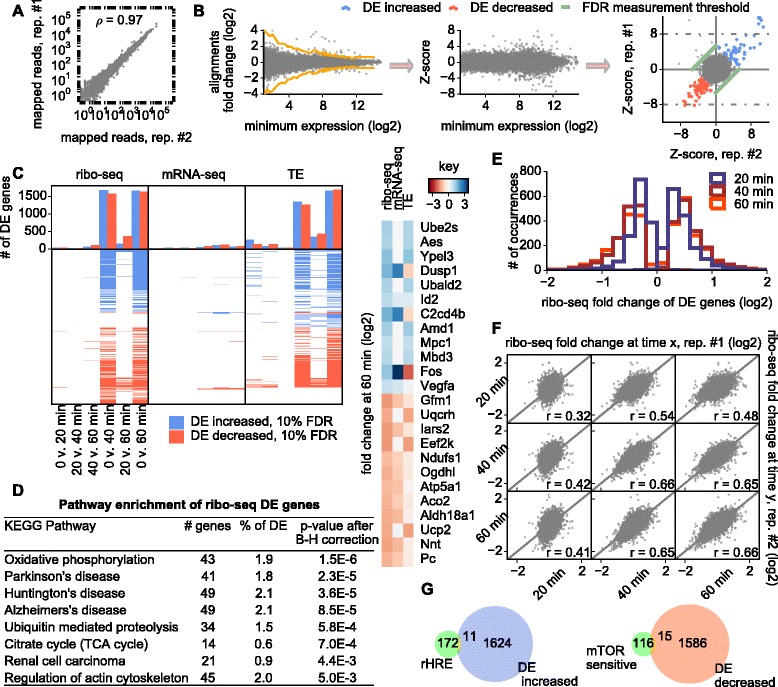
Analysis of differential gene expression. **(A)** Reproducibility of ribo-seq experiment under control conditions. **(B)** Z-score transformation and false discovery rate (FDR) estimation. **(C)** Heat map diagram of differentially expressed (DE) genes. TE, translation efficiency. **(D)** DE gene enrichment over KEGG (Kyoto Encyclopedia of Genes and Genomes) pathways. B-H, Benjamini-Hochberg. **(E)** The magnitude of ribo-seq occupancy alteration during the first 20, 40 and 60 minutes of OGD for the genes detected as DE after 40 minutes. **(F)** Correlations across replicates of ribo-seq fold changes of genes with >32 reads per time point at different time intervals of OGD exposure. **(G)** Venn diagrams showing the lack of a relationship between DE genes, mTOR sensitivity and the presence of rHRE.

Ribo-seq and mRNA-seq libraries were obtained for two independent biological replicates (Figure 2A; Figure A3 in Additional file 1) with between 7 and 17 million non-ribosomal RNA reads mapped from each library. The gene expression exhibited a neuronal signature with high expression of *Th* and *App*. Differentially expressed (DE) genes were identified upon Z-score transformation [16] followed by the determination of the 10% false discovery rate (FDR) threshold (Materials and methods; Figure 2B). RNA levels of 111 genes were found to be altered after 1 h of OGD (Figure 2C). The function of many of these genes is consistent with a response to bioenergetic stress as evident from frequently associated gene ontology terms: ‘response to reactive oxygen species’ (*Dusp1*, *Fos*, *Hspd1*), ‘response to hypoxia’ (*Actn4*, *Abat*, *Adm*, *Ddit4*, *Egln3*, *Vegfa*), ‘oxidative phosphorylation’ (*Atp5a1*, *Ndufs1*, *Ndufs2*, *Sdha*, *Uqcrc1*), ‘tricarboxylic acid (TCA) cycle’ (*Aco2*, *Idh3g*, *Ogdh*, *Ogdhl*, *Pck2*, *Pc*).

The same statistical procedure uncovered an overwhelming response at the level of translation. Analysis of ribo-seq occupancy revealed 3,202 DE genes after the first 40 minutes of OGD. Analysis of translation efficiency (ribo-seq signal normalized over mRNA) revealed 2,556 DE genes (Figure 2C) during the same period. Moreover, the greater part of this response occurred within the first 20 minutes (Figure 2E). The fold change of gene expression after the first 20 minutes correlated well with that after 40 and 60 minutes of OGD (Figure 2F). This suggests that most of the later response is an amplification of the differential expression that occurred during the first 20 minutes. Note, there seems to be a delayed response in replica 2 compared with replica 1 for the 20 minute data point. However, the trend of changes is the same across the entire time period. The variance observed between replicas at 20 minutes of stress may not be coincidental. If changes occur most rapidly around this time point, increased variance in response would be expected for that data point.

The enrichment analysis of the KEGG (Kyoto Encyclopedia of Genes and Genomes) pathways associated with DE genes (ribo-seq occupancy) revealed an overrepresentation of genes associated with the oxidative phosphorylation pathway and neurological diseases in which oxidative phosphorylation plays a significant role (reviewed in [17,18]; Figure 2D; Figure A4 in Additional file 1). Genes involved in ubiquitin proteolysis and the TCA cycle were also overrepresented (Figure 2D; Figure A5 in Additional file 1).

It has been shown previously that, upon prolonged hypoxia (24 h), Epas1 (also known as HIF-2α) induces translation of mRNAs bearing rHRE in their 3′ UTRs [8]. This occurs during general translation suppression by eIF4E sequestration. Therefore, we explored the response of genes containing rHRE [8], as well as of genes whose mRNA translation is mTOR (mammalian target of rapamycin) sensitive [19]. Neither group of genes significantly overlaps with DE genes (Figure 2G), suggesting that these pathways are unlikely to be involved in the early response to OGD.

### Ribosome density increases at mRNAs 5′ leaders during oxygen and glucose deprivation

**Figure 3:**
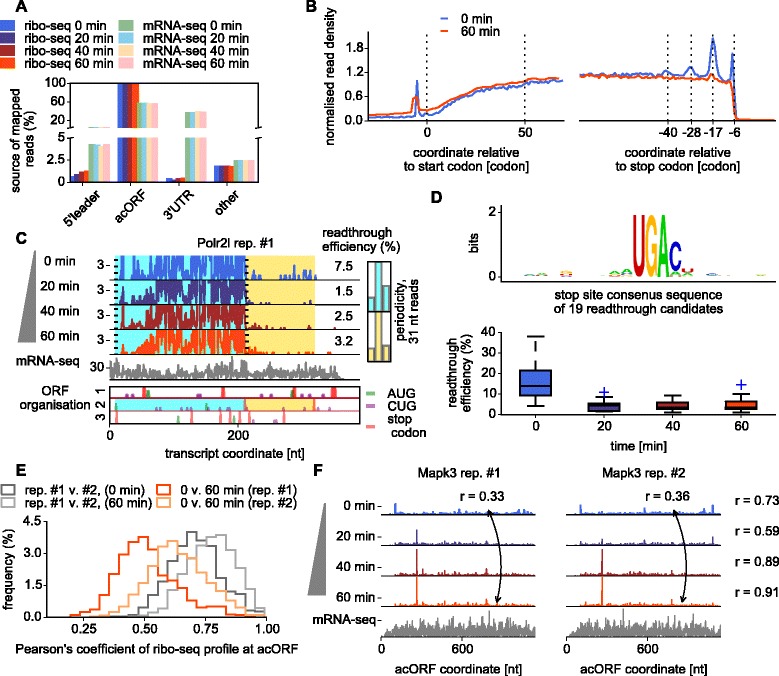
OGD-induced changes to initiation, elongation and termination. **(A)** Distribution of sequenced fragments across functional regions of mRNAs under different conditions. **(B)** Metagene profile of the ribosome density obtained under normal condition and after 1 h of OGD. Positions of footprints 5′ ends are shown relative to the coordinates of the start and stop codons of acORFs, which are set to 0. Over 5,000 mRNA profiles were aggregated to produce the profile. **(C)** Ribosomal density at *Polr2l* mRNA. The density in the 3′ UTR indicates leaky termination (readthrough). **(D)** Sequence logo of the nucleotide context surrounding stop codons in the 19 mRNAs exhibiting high levels of readthrough (top) and the change of stop codon readthrough efficiency during the time course of OGD. **(E)** Distribution of the Pearson’s correlations of ribosome densities for individual mRNA profiles between replicas and across conditions. **(F)** An example of mRNA (*Mapk3*) with changes in footprint density distribution between conditions and replicas.

In agreement with previous ribosome profiling studies of stress responses [20-22] we observed a widespread increase of ribosome density in mRNA 5′ leaders upstream of annotated coding ORFs (acORFs; Figure 3A). A metagene profile analysis indicates that this increase spreads throughout the entire 5′ leader and the beginning of the coding regions (Figure 3B). It has been argued recently that the increase in 5′ leader translation may be an artifact of ribosome accumulation in the vicinity of start codons caused by cycloheximide pretreatment blocking elongation but not scanning ribosome particles [23]. Therefore, it is important to emphasize that, in our procedures, we did not pretreat cells with cycloheximide and still observed the increase despite reduced ribosome densities at the beginning of acORFs in all conditions (Figure 3B).

### Effect of oxygen and glucose deprivation on translation termination

A striking feature of metagene profiles is the presence of distinct peaks of ribosome density at the 3′ ends of coding regions under control conditions (Figure 3B; Figure A6 in Additional file 1). The peaks are separated by 11 to 12 codon intervals roughly corresponding to the length of the ribosome protected fragment. Therefore, we believe that they occur due to ribosome queuing at the ends of coding regions of some mRNAs. This stacking is independent of the identity of stop codons and is observed in metagene profiles constructed based on the identity of stop codons (Figure A6B in Additional file 1). Indeed, it appears to be widespread among all mRNAs (Figure A7 in Additional file 1). Presumably the stacking occurs due to a slow rate of translation termination relative to elongation. The stacking disappears under OGD stress due either to an increased rate of translation termination or to a reduction of elongation rates.

Puzzled by this observation, we explored how OGD affects the accuracy of translation termination. We found 19 mRNAs with relatively high density of footprints immediately downstream of annotated stop codons. An example profile for *Polr2l* mRNA is shown in Figure 3C, and profiles for all other mRNAs can be found in Additional file 2. All of these mRNAs have acORFs terminating in a UGA codon, most of which have an adjacent 3′ C (Figure 3D), which is known to be frequent among mammalian genes that were recently discovered to utilize stop codon readthrough in their expression [24-26].

The efficiency of termination at these 19 genes increased considerably by 20 minutes of OGD and remained high during the rest of the time course (Figure 3D). This was surprising in the light of evidence that hydroxylation of eRF1 is known to promote termination efficiency [27]. However, it has also been shown recently that protein components of the ribosome decoding center in *Saccharomyces cerevisiae* is subject to hydroxylation and the hydroxylation affects stop codon readthrough in opposite ways depending on the stop codon and nucleotide context surrounding it [28]. The structures of oxygenases are highly similar across all kingdoms of life [29] and it is very likely that mammalian ribosomes could be affected in a similar way.

### Oxygen and glucose deprivation alters local decoding rates

The phosphorylation of eEF2 (Figure 1D) led us to examine the profile data for changes in translation rates. The density of ribosomes at any specific location of an acORF is expected to negatively correlate with the local rate of ribosome elongation, but to positively correlate with the rate of translation initiation for that acORF. Therefore, if the translation initiation rate at the acORF is changed but local elongation rates are unchanged, we expect the geometric shape of the ribosome profile to remain the same. To explore whether this is the case we measured pairwise profile similarities (by calculating Pearson’s correlation coefficients) across conditions and between replicas (Figure 3E; Figure A8 in Additional file 1).

We found that the ribosome density distributions over individual mRNAs correlated better between replicas for the same conditions than within replicas for different conditions, indicating that OGD induces alterations in the local elongation rates. We excluded the possibility that differences of mRNA sequencing depth was a confounding factor by repeating the analysis with pairwise comparison of profiles of equal sequencing depth (Figure A8A in Additional file 1). The change of profile shape was caused by both the alleviation of pauses observed under normal conditions and the induction of new pauses (Figure A8B in Additional file 1). In some cases, these alterations resulted in reproducible peaks of ribosomal density with their heights increasing as OGD progresses, as exemplified by the *Mapk3* mRNA profile in Figure 3F (see Figure A9 in Additional file 1 for more examples). These peaks most likely represent OGD-induced ribosome pauses. A simple sequence logo examination [30] of the nucleotide or amino acid context in the vicinity of the strongest pauses did not reveal a common sequence motif associated with them.

### Oxygen and glucose deprivation alters the recognition of translation initiation sites

**Figure 4:**
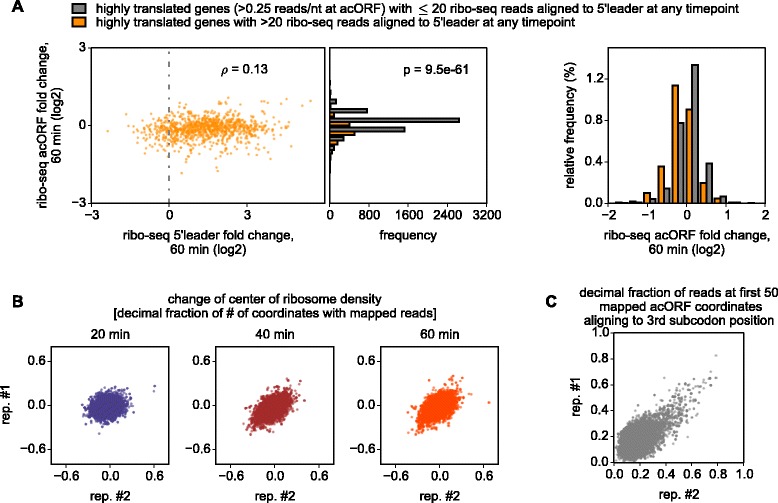
The effect of 5′ leader translation on translation of acORFs and identification of regulatory uORFs. **(A)** Correlation between fold change of ribosome density in the 5′ leaders and in acORFs and the change in acORF translation in mRNAs with translated 5′ leaders (orange bars) relative to all mRNA (grey): absolute (left) and relative (right) frequencies of genes. **(B)** Change in the position of the centre of ribosome density for each mRNA during the course of OGD. mRNAs with upregulation of inhibitory uORFs are likely to be found in the left bottom corner. Ribosome profiles of corresponding mRNAs were chosen for manual examination. **(C)** acORF regions translated in more than one reading frame are expected to have altered triplet periodicity (high density of footprints with 5′ ends at the third subcodon position). This analysis was used to detect overlapping uORFs.

**Figure 5:**
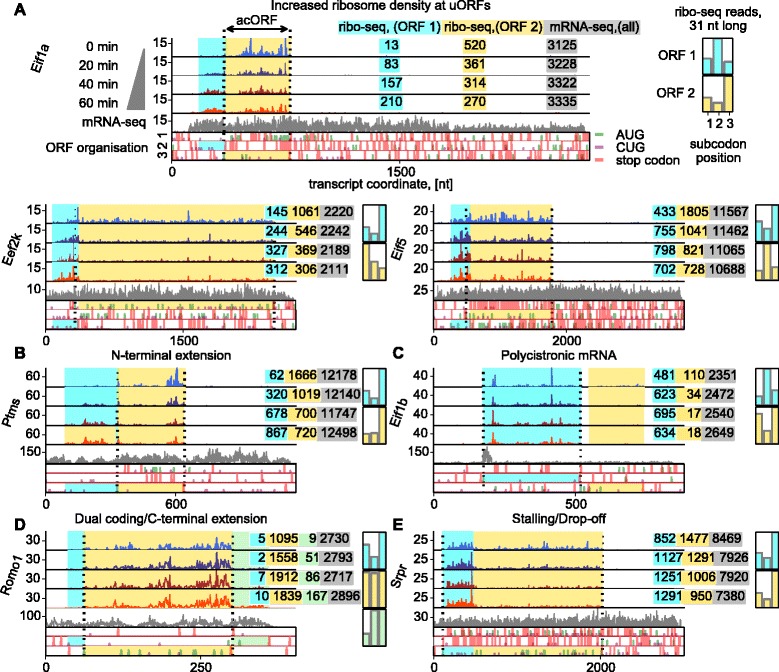
Ribosome profiles exhibiting mRNA-specific OGD-induced features. **(A)** Selected mRNAs whose ribosome densities increase at uORFs and decrease at acORFs during OGD. Ribosome profile for *Eif1a* mRNA is used as the legend. **(B)** Upregulation of parathymosin isoform with amino-terminal extension whose synthesis begins at a non-AUG codon. **(C)** Increased uORF density at *Eif1b* mRNA coincides with decreased density at the acORF and non-annotated downstream ORF. **(D)** Increased ribosome density downstream of *Romo1* acORF that may occur as a result of translation of a long overlapping ORF. **(E)** Ribosome stalling or an abortive translation event at *Srpr* mRNA.

We considered the possibility that the increase in ribo-seq reads may have contributed to the translational response by inhibiting translation of acORFs. Although the fold change of ribo-seq occupancy does not correlate well with that of the acORFs globally, we observed that translation of acORFs at mRNAs with translated 5′ leaders is more likely to be downregulated in comparison with the rest (Figure 4A). Therefore, it is likely that part of the translational response is due to the inhibitory roles of upregulated uORFs. We screened for such cases by using two metrics, the shift of the center of ribosome density [31] towards the 5′ end and the triplet periodicity signal at the start of acORFs [32,33] (Figure 4B,C). The triplet periodicity was used to infer the phase of translated frames, while a shift in the center of density is observed when translation is altered differently at the 5′ part of mRNAs (for example, uORFs) and at the 3′ part of mRNAs (acORF region that does not overlap any uORFs). Inhibitory uORFs should display a shift in density owing to the expected increase in 5′ leader occupancy and subsequent decrease in acORF occupancy. If a uORF overlaps an acORF, its translation would distort the triplet periodicity of ribosome footprints in the region of the overlap [32]. The screen was followed by manual evaluation of mRNAs and analysis of ribosome density changes in uORFs relative to acORF. We identified several dozen mRNAs for which translation of their uORFs increased during OGD with an accompanying decrease in acORF translation. Many of these uORFs lack AUG codons. Ribosome profiles of *Eif1a* and *Eef2k* mRNAs exemplify such regulation (Figure 5A). More examples of AUG and non-AUG initiated uORFs can be found in Additional file 2. The strongest increase in translation of uORFs was observed for *Eif1a* and *Junb* mRNAs. For these genes, ribosome density on uORFs increased over 10-fold during OGD from almost undetectable levels under normal conditions, and greatly exceeded ribosome density at the acORFs (Figure 5A; Additional file 2).

The likely mechanism of the increased uORF translation is the altered recognition of translation initiation sites. This is supported by the observed decrease in translation of acORF in *Eif5* as well as in *Eif1a* and its paralogs *Eif1ad* and *Eif1ax*, which are all implicated in start codon selection [34-36]. Interestingly, it is likely that decreased production of these factors is also mediated by uORF translation (Figure 5A; Additional file 2). Synthesis of another important factor controlling stringency of initiation site selection, eIF1 [37], increased about two-fold (Additional file 3). eIF1 and eIF5 have opposing effects on the stringency of start codon selection [38] and both regulate translation of their own mRNAs via negative control feedback loops. The negative feedback control mechanism is modulated by the initiation context in *Eif1* [39]. In *Eif5* the mechanism involves translation of inhibitory uORFs [38]. The inhibition of eIF5 synthesis and increased eIF1 synthesis under OGD are synergistic responses, expected to counteract the reduction of start codon selection stringency.

Another important example of uORF-mediated inhibition of acORF translation is in *Eef2k* mRNA (Figure 5A). The observed reduction of *Eef2k* acORF translation is consistent with a reduction in the levels of eEF2k protein (Figure A2D in Additional file 1). Once activated by AMPK, eEF2k phosphorylates eEF2 and induces a general suppression of translation [40-42]. Thus, the uORF-mediated inhibition of *Eef2k* expression counteracts activation of its protein product and may serve as a break mechanism to avoid complete suppression of translation elongation.

### Novel protein isoforms, and polycistronic and dual coding mRNAs

The altered recognition of translation initiation sites can potentially initiate translation at non-AUG codons that are in-frame with acORFs, leading to the production of protein isoforms with amino-terminal extensions. Evolutionarily conserved extended isoforms are known [43]; some of them have been shown to have important biological functions, for example, in PTEN [44]. Here we detected translation of OGD-induced extended protein isoforms with upstream in-frame non-AUG initiation sites of five genes, *Adm*, *Bcl211*, *Fam178b*, *Ptms*, and *Ppp1r2* (Figure 5B; Additional file 2). In *Ptms* mRNA, encoding parathymosin, under normal conditions nearly all ribosome footprints are concentrated within the acORF. After 1 h of OGD, the ribosome density upstream of the acORF increased 15-fold (Figure 5B).

Whereas translation of uORFs in 5′ leaders seems to be widespread in mammalian mRNAs, translation of ORFs downstream of acORFs is infrequent. One known example is the bicistronic mRNA *Rpp14* [45]. The first acORF of this mRNA codes for ribonuclease P subunit (Rpp14) while the second acORF codes for 3-hydroxyacyl-thioester dehydratase 2 (HsHTD2) [45]. Both appear to be inhibited by OGD-induced uORF translation. Intrigued by these cases we searched for other examples of mRNAs with translated ORFs located in 3′ UTRs. In total we identified six candidate bicistronic mRNAs (available in Additional file 2); one of the most highly translated downstream ORFs is in *Eif1b* mRNA (Figure 5C)*.* While translation of the acORF encoding eIF1b is increased during OGD, translation of the downstream ORF is inhibited (Figure 5C).

In *Romo1* mRNA, which encodes a reactive oxygen species modulator, we observed increased ribosome density immediately downstream of the acORF (Figure 5D). This may indicate a regulated carboxy-terminal extension due to frameshifting, stop codon readthrough or translation of a long non-AUG-initiated overlapping ORF. The latter is supported by the lack of clear triplet periodicity of ribosome footprints aligning to the acORF (presumably due to the simultaneous translation of two frames). A similar situation is observed in mRNA of *Amn* (available in Additional file 2).

Particularly interesting is the OGD-induced pause occurring in *Srpr* mRNA which encodes the β subunit of the signal recognition particle (SRP) receptor. Unlike other position-specific pauses that we described earlier (Figure 3F), *Srpr* pause is accompanied by decreased ribosome density downstream of the pause site (Figure 5E). This may occur in case of abortive translation due to ribosomal frameshifting or drop-off. Expression of truncated SRPR may influence endoplamic reticulum-localized translation.

## Conclusions

Using ribosome profiling we explored the immediate response of neural cell gene expression to OGD at the transcription and translation levels. This condition, mimicking ischemia, imposed a dramatic stress on cellular bioenergetics and mitochondrial function (Figure 1A,B). The most surprising finding is the widespread effect of OGD on translation in comparison with its effect on the transcriptome. While RNA levels of only slightly more than a hundred genes were affected during the course of 1 h OGD, translation of about a fifth of all expressed genes underwent significant alterations. The other striking observation is the speed of the response: most of the changes occurred rapidly within the first 20 minutes and were slowly amplified during continuation of the exposure to OGD (Figure 2). Rapid translational response (<30 minutes) has been described for a number of changing conditions, such as a change in the source of carbon in *S. cerevisiae* growth media [46] and oxidative stress [47]. In mammalian cells, fast translational response has been observed in response to certain drug treatments, for example, thapsigargin-induced unfolded protein response [48], arsenite-induced eIF2 phosphorylation [31] and tunicamycin-induced endoplasmic reticulum stress [49]. Therefore, a rapid translational response preceding (and likely shaping) the transcriptional response could be a general mode of how cells regulate their gene expression in reaction to various stimuli. The analysis of general translation parameters suggests that there is unlikely a single regulatory mechanism. The involvement of a multitude of factors is evident from global alteration of translation which include: increased translation of 5′ leaders similar to what has been observed in studies of translation response to other stresses [21,22,48]; numerous OGD-induced pauses that are indicative of context-dependent local changes in elongation rates; reduced stringency of start codon selection in translation initiation sites and improved accuracy of translation termination. The ceasing of ribosome queuing at the ends of mRNAs could be due to either increased translation termination or decreased elongation rates; the latter is anticipated as a result of eEF2 phosphorylation. It would be very interesting to identify individual causative factors responsible for these changes. Our study points to some potential players involved - a number of mRNAs whose translation was altered encode proteins involved in translation regulation, such as certain translation initiation factors and eEF2k, the only kinase targeting eEF2. The changes in the translation of these mRNAs may well be due to autoregulatory mechanisms.

Despite the widespread effect that OGD had on gene expression, the response targets functionally relevant genes (particularly at the transcriptional level) as is evident from gene ontology analysis of DE genes. Oxidative phosphorylation was affected the most along with the closely related TCA cycle, as well as neurodegeneration pathways and ubiquitin dependent proteolysis (Figure 2D).

We also conducted detailed characterization of individual translation responses for a number of affected mRNAs. This revealed dozens of regulatory uORFs (many of them are non-AUG initiated), translation of extended protein isoforms, six polycistronic mRNAs and two novel dual coding mRNAs.

This study suggests that cellular gene expression reacts to OGD prior to (or even instead of) the HIF-promoted transcriptional response by altering translation of available mRNAs. It is intriguing to speculate that some components of the translational machinery are regulated by oxygen availability and trigger specific responses upon oxygen deprivation. Indeed, a number of translation components have been shown to be hydroxylated under normoxic conditions [14,28]. It is likely that some of the alternative translation products and protein isoforms detected in this study have crucial implications in cell responses to OGD. Their functional roles under this stress merit further investigation.

## Materials and methods

### Materials

TMRM and JC-1 probes were from Invitrogen Life Technologies (Carlsbad, CA, USA and Dun Laoghaire, Ireland). Amersham™ ECL™ Prime Western blotting reagent was from GE Healthcare Life Sciences (Waukesha, WI, USA), pre-made acrylamide gels, running and transfer buffers were from GeneScript (Piscataway, NJ, USA), RIPA buffer, BCA™ Protein Assay kit and Pre-stained Protein Ladder were from Thermo Fisher Scientific (Rockford, IL, USA). CellTiter-Glo® ATP Assay was from Promega (Madison, WI, USA). PhosphoStop Phosphatase Inhibitor and complete Protease Inhibitor Cocktail Tablets were from Roche (Dublin, Ireland). Dulbecco’s Modified Eagle’s medium (DMEM), Roswell Park Memorial Institute (RPMI) media, cycloheximide and all the other reagents were from Sigma-Aldrich (St. Louis, MO, USA). Primary and secondary antibodies are listed in Additional file 4. Plastic and glassware were from Sarstedt (Ireland), Corning Life Sciences (Corning, NY), Greiner Bio One (Frickenhausen, Germany) and Pecon (Erbach, Germany).

### Tissue culture and experimental conditions

PC12 cells from ATCC (<40 passages) were maintained in suspension in RPMI 1640 medium supplemented with NaHCO_3_, 2 mM L-glutamine, 10% horse serum, 5% fetal bovine serum, 100 U/ml penicillin and 100 μg/ml streptomycin, in a humidified incubator set to 5% CO_2_ and 37°C. For all experiments, PC12 cells were seeded at 4 to 6 × 10^4^ cells/cm^2^ on 75 cm^2^ flasks, 10 cm or 15 cm Petri dishes and 96-well plates (all coated with collagen IV at 0.01 mg/ml) or 4.2 cm glass coverslips (PeCon, Erbach, Germany), coated with a mixture of collagen IV and poly-D-lysine [50]. Cells were maintained in the adherent state for up to 3 days. In a cell monolayer O_2_ is distributed more uniformly than in small clamps, common for PC12 cell suspension [51]; therefore, data variability was expected to be lower for adherent cells.

### OGD and OD experiments

Media for OGD and O_2_ deprivation (OD) were prepared similarly to [50] as follows. Powder DMEM (Sigma, catalog number 5030) was reconstituted in deionised water, buffered with 20 mM HEPES (pH 7.2) and filter-sterilized. Using this plain DMEM, the experimental medium were composed by addition of 10 mM glucose, 2 mM glutamine and 1 mM pyruvate (OD medium) or glutamine and pyruvate only (OGD). No serum was added. Media were equilibrated for 20 h at 0% O_2_ using the hypoxia workstation (Coy Laboratory Products, Grass Lake, MI, USA). Media deoxygenation was monitored using pre-calibrated phosphorescent dOxyBead™ sensors (Luxcel Biosciences, Cork, Ireland) and OpTech™ O_2_ Platinum handheld detector (Mocon, MN, USA) (Figure A1 in Additional file 1).

Adherent cells grown in a regular CO_2_ incubator were transferred to the hypoxia workstation (Coy), and equilibrated with 95% N_2_ and 5% CO_2_ (0% O_2_) at 37°C. Growth medium was rapidly replaced with deoxygenated OGD or OD media: 20 ml for 15 cm Petri dish, 10 ml for 10 cm dish, 1 ml for 4.2 cm coverslip, 100 μl for 1 well of a 96-well plate. Cells were then incubated in anoxic conditions for 20 minutes, 40 minutes, 1 h, 2 h and 4 h. Control cells were exposed to the oxygenated OGD or OD media under normoxia for 1 h. At the time points indicated, cells were rapidly extracted from the workstation and subjected to analysis.

### Generation of ribo-seq and mRNA-seq libraries

The ribosomal profiling technique was carried out as in Ingolia *et al*. [52] but with a few modifications as outlined in Andreev *et al*. [31]. Libraries were sequenced on an Illumina HiSeq 2000 system at the Beijing Genomics Institute (BGI).

### Protein isolation and western blotting analysis

Standard analysis of whole cell lysates prepared using RIPA buffer was performed using western blotting analysis as in Zhdanov *et al*. [50]. Quantitative data analysis was conducted with the ImageJ program using α-tubulin signals for normalization. Images were processed with Picasa, Photoshop, and Illustrator programs. Experiments were carried out in triplicates.

### ATP measurement

Cellular ATP was measured using CellTiter-Glo® Assay (Promega), which allows for the end-point high throughput quantitative analysis of cellular energy state. Pre-mixed kit reagents (100 μl) were added to the cells directly in the hypoxia workstation. Plates with cell lysates were transferred to normoxia. After intensive shaking the lysates were transferred into white 96-well plates (Greiner Bio One, Frickenhausen, Germany) and their luminescence was measured on a Victor 2 reader.

### Measurement of the mitochondrial membrane potential

For monitoring of the mitochondrial membrane potential (ΔΨm) we utilized two commercial fluorescent probes, TMRM and JC-1. When used at non-quenching concentrations (1 to 20 nM), TMRM rapidly accumulates in polarized mitochondria and is easily released from depolarized mitochondria. TMRM is also sensitive to the changes in the plasma membrane potential; therefore, additional controls are required for ΔΨm analysis. Upon the accumulation in the polarized mitochondria, JC-1 exhibits a split to green and red fluorescent spectra. The latter is only produced by highly polarized (‘energized’) mitochondria, when JC-1 forms J-aggregates. To ensure equal loading conditions, we stained the cells at 21% O_2_ for 25 minutes (1 μM JC-1 and 20 nM TMRM). The TMRM probe was maintained in the media at 20 nM during the whole experiment. For imaging, the coverslips with cells were locked in a mini hypoxia chamber in approximately 1 ml of the oxygenated or deoxygenated medium, using three silicon ring gadgets and a second coverslip, which were sandwiched together to be air-tight with a metallic base with the help of the screwing cap as shown in Figure A1D in Additional file 1. Upon incubation of the cells as described in the Results section, ΔΨm was then quickly monitored (within 10 minutes) on a wide field fluorescence microscope (Zeiss, Germany). TMRM was excited using a 590 nm 10 mW LED with emissions collected at 604 to 644 nm. JC-1 was excited at 488 nm and 590 nm, while collecting emissions at 510 to 550 nm and 604 to 644 nm, respectively.

### Initial processing of sequence libraries

Cutadapt [53] was used for the removal of the adapter sequence (CTGTAGGCACCATCAATAGATCGGAAGAGCACACGTCTGAACTCCAGTCA). The reads were then aligned to rRNA and the introduced ‘spike-in’ (ATGTACACGGAGTCGACCCGCAACGCGA) to remove reads that originated from these sources. Reads were aligned to the rat RefSeq gene catalogue downloaded from NCBI on January 2014 [54] with bowtie [55]. The alignment parameters used were (−a -m 100 -v 2 -norc), that is, reads were aligned to the positive strand allowing no more than two mismatches and no more than 100 mappings per read.

### Differential gene expression analysis

For differential expression analysis we calculated the number of reads aligning to an exon union of a gene [56]. Any read aligning to any of the RefSeq transcripts originating from the same gene was counted once irrespective of the number of isoforms to which it aligns. Such a gene centric approach should provide a more accurate representation of gene expression than alignment to the longest transcript, since even the longest transcript may not contain all exons. Approximately 2% more reads were aligned to the exon unions of the genes in comparison with the alignment to the longest transcripts. For certain genes (including *Nnat*, *My16*, *Cabin1*) we observed more than a two-fold increase in the number of aligned ribo-seq and mRNA-seq reads.

Ribo-seq reads were assigned to mRNA coordinates based on the inferred location of the A-site. The A-site location was set at 17 nucleotides downstream of the 5′ end of reads of length 29 to 33 inclusive and 18 for reads of length 34 and 35. Ribo-seq reads of length less than 29 or greater than 35 were discarded. For genes that had transcripts with an annotated coding region we used only the ribo-seq reads aligning to it. Otherwise the ribo-seq read mapped to the entire transcript were included (note that for the mRNA-seq, the reads aligning to the entire transcript were used). Some ribo-seq reads were mapped to more than one region of a transcript. We assigned them to unique locations based on the following priority order: acORF, 5′ leader, 3′ UTR.

Approximately 80% of mapped ribo-seq reads were mapped to a single gene, 9% mapped to two genes and 4% mapped to three. For the mRNA-seq reads these values are 84%, 8% and 3%, respectively. Ambiguously aligned reads that aligned to more than three locations were discarded. The weighting of reads aligned to two or three locations was reduced by 2 and 3, respectively.

Normalization (the rescaling of read counts to remove the differences due to the total number of mapped reads) and differential analysis were carried out with an approach similar to the one we used previously [31]. The read counts aligning to a feature in the sample *k* were multiplied by the rescaling factor *x*[*k*]/*min*(*x*[*i*]) where *x*[*k*] is the number of all sequence reads aligning to all features within the sample *k*, and *min*(*x*[*i*]) is the number of all reads aligning to all features in the sample *i* which has the smallest number of reads. This rescaling was carried out independently for the mRNA-seq and ribo-seq data, but both replicates were normalized together to enable comparison between replicates.

To identify DE genes we carried out Z-score transformation of log ratios [16]. Genes were grouped in bins of 300 based on the minimal level of their expression (minimal number of mRNA-seq, ribo-seq or both depending on the analysis). The parameters of expression fold-change distribution were used to calculate local Z-score for every gene. The gene was classified as DE increased if (*Z*_1_ + *Z*_2_)/2 > *T* and DE decreased if (*Z*_1_ + *Z*_2_)/2 < −*T*, where *Z*_1_ and *Z*_2_ are Z-scores for the same gene obtained in two replicas and *T* is the threshold. The threshold *T* was set based on the desired FDR. The number of false positives was estimated as the number of genes for which (*|Z*_1_| + |*Z*_2_|)/2 > *T* when *Z*_1_*Z*_2_ < 0 (see Figure 2B for a graphical illustration of the procedure).

Owing to misalignments, amplification biases and changes in elongation rates between the conditions, the total number of reads mapped to a gene is not necessarily an accurate representation of its level of expression. For example, a strong condition-dependent ribosome stalling at a specific location could lead to spurious upregulation. A gene was considered robustly regulated only if it was detected as regulated after exclusion of acORF coordinates corresponding to the three highest density peaks of the ribosome density for each of its transcripts. The analysis of peak exclusion effect on detection of differentially expressed genes is illustrated in Figure A10 in Additional file 1. In this image, the genes were clustered by the Scipy linkage function using the ‘single’ linkage criteria and ‘hamming’ column distance metric.

### Analysis of pairwise similarity of ribo-seq profiles of individual mRNAs

The pairwise similarity between two acORF ribo-seq profiles was assessed by calculating Pearson’s correlation coefficients for local footprint densities of compared profiles. Only unambiguously aligned ribo-seq reads were used for this analysis. The position of the reads aligning to the acORF region was determined at a codon, rather than at a nucleotide level. Only the longest transcript variants of each gene that had an average acORF density exceeding one footprint per nucleotide in both compared samples were used in this analysis. For the analysis in Figure A8B in Additional file 1 the process was repeated using alignments produced by randomly sampling a fixed number of reads for each mRNA from the real data. In each pairwise comparison, for the conditions with the higher sequence coverage ribo-seq reads were sampled from the original alignment until the number of reads equalled that for a condition with lower sequencing coverage. The average pairwise Pearson’s coefficient of these profiles were used to produce the distribution.

### Identification of genes with leaky termination

The profiles of transcripts that had a total number of ribo-seq reads aligning to their 3′ UTR greater than 20 were manually examined. Ambiguous alignments were included. The selection was made without prior knowledge of the stop codon identity and nucleotide context. The context of termination sites was visualized using a sequence logo produced with weblogo [30]. The ratio of ribo-seq read density (Alignments at ORF/Length of ORF) at the acORF and downstream ORF was used to measure stop codon readthrough efficiency.

### Identification of translated uORFs

The probability that the distributions of highly translated genes with and without a translated uORF (Figure 4A) are sampled from the same distribution was calculated by Wilcoxon rank sum test implemented with the ranksums function in Scipy library. To enrich the gene pool for those with regulatory uORFs, we analyzed the shift of the center of ribosome density [31]. To determine the center of ribosome density we first produced ‘compressed profiles’, where every coordinate of an mRNA profile that did not contain an alignment in at least one of the time points was removed. This has the advantage of producing a more robust result with a sparse number of reads. The center of ribosome density was defined as the minimal coordinate of a compressed profile at which the cumulative number of ribo-seq reads aligning upstream of it exceeds the cumulative number of reads downstream of it. The shift of the center was measured relative to the length of the translated regions of mRNAs, that is, divided by the number of coordinates with at least one ribo-seq read alignment. The change of center of density was obtained for all annotated coding transcripts with greater than 64 ribo-seq read alignments. Profiles of over 200 transcripts with the greatest average shift of density towards the 5′ end after 1 h of OGD (Figure 4B) were manually evaluated.

As the strongest triplet periodicity was observed with ribo-seq reads of 31 nucleotides in length, these alone were used for the analysis of the triplet periodicity signal. To identify cases with an atypical periodicity, we used the alignments to the first 50 coordinates of the acORF. For most transcripts approximately 20% of reads aligned to the third subcodon position; a higher proportion of ribo-seq reads aligning to the third subcodon position was considered as an indicator of triplet periodicity distortion. Transcripts containing less than 50 coordinates with aligned ribo-seq reads were excluded from this analysis.

### Production of metagene profile at initiation and termination sites

A transcript used for generating a metagene profile was selected to satisfy the following criteria: 1) it is the longest among other transcript variants for the corresponding gene; 2) it has at least 100 mapped alignments; 3) its length is greater than 600 nucleotides; 4) the length of both its 5′ leader and 3′ UTR is greater than 45 nucleotides. Each individual transcript profile 45 nucleotides upstream of the initiation site and 45 nucleotides downstream of the termination site was normalized by the average gene density before aggregation by the determination of a position-specific average. This was converted to the average number of alignments per codon.

### Analysis of biological pathways

The 3,000 genes found to have the greatest absolute average ribo-seq Z-score after 60 minutes of OGD (DE increased and DE decreased ) were used for gene ontology and KEGG pathway enrichment analysis using DAVID [57]. The statistical significance of gene enrichment was corrected for multiple testing using the Benjamini-Hochberg method. DE genes found to belong to oxidative phosphorylation and the TCA cycle are highlighted in Figures A4 and A5 in Additional file 1.

### rHRE-containing and mTOR-sensitive mRNAs

The list of transationally regulated PP242 responsive genes from Supplementary Figure 5 of Hsieh *et al*. [19] was used as a set of mTOR-sensitive genes. The set of transcripts containing rHRE was built from Supplementary data 2 of Uniacle *et al*. [8] requiring at least three PAR-CLIP reads supporting the EPAS1/RBM4 target.

All computations and plots were produced with custom python scripts using Matplotlib library.

### Data access

Sequences of ribo-seq and mRNA-seq cDNA libraries have been deposited in the NCBI Genome Expression Omnibus (GEO) under accession number GSE60752.

## Additional files

Additional file 1:
**Additional Figures A1 to A10.**
Additional file 2:
**This file contains a mini web site with additional ribosome profiles of individual mRNAs that are mentioned in the manuscript.** The same mini web site is available at http://lapti.ucc.ie/ogd/. Additional file 3:
**Meta-data for sequencing and alignment, differentially expressed genes, further information on novel translated segments, raw and normalized counts, Z-scores for RNA-seq, ribo-seq and TE signals for all conditions and time points.**
Additional file 4:
**Additional Table.** Primary and secondary antibodies used.

## Abbreviations

acORF: annotated coding ORF
AMPK: AMP-activated protein kinase
DE: differentially expressed
DMEM: Dulbecco’s Modified Eagle’s medium
FDR: false discovery rate
GEO: gene expression omnibus
HIF: hypoxia inducible factor
HRE: HIF-responsive element
KEGG: Kyoto encyclopedia of genes and genomes
mTOR: mammalian target of rapamycin
OD: oxygen deprivation
OGD: oxygen and glucose deprivation
ORF: open reading frame
rHRE: RNA hypoxia responsive element
uORF: upstream ORF
UTR: untranslated terminal region
TCA: tricarboxylic acid
