# Supplementary material for: Oxygen and glucose deprivation induces widespread alterations in mRNA translation within 20 minutes
Source: Genome Biol. 2015 May 6;16(1):90. doi: 10.1186/s13059-015-0651-z (PMC4419486; doi:10.1186/s13059-015-0651-z)
Supplement: Additional file 1: — Additional Figures A1 to A10. [file 13059_2015_651_MOESM1_ESM.pdf]

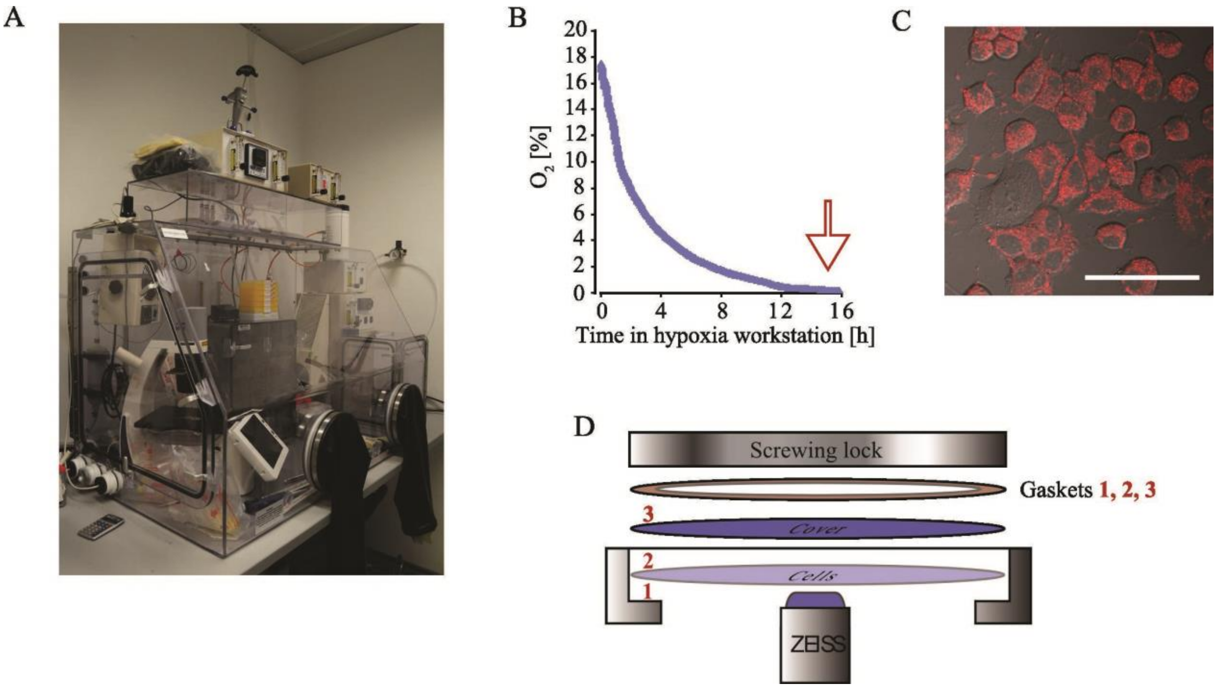

Additional Figure A1. Setup of OGD, medium oxygenation control and cell microscopy experiments.

(A) Hypoxia workstation (Coy Laboratory Products). **(B)** Medium deoxygenation profile measured using OpTech™ O_2 platinum system for 16 h after transferring of the medium (200 mL) from 21% to 0% atmospheric O_2 in the workstation. Arrow shows the time point of complete medium deoxygenation, when OGD experiments may proceed. **(C)** Mitochondrial network in PC12 cells demonstrated by TMRM staining. Bar is 50 µm. **(D)** Portable mini-chamber used for the mitochondria depolarisation study (POC-R perfusion adapter, Pecon). For the microscopy analysis, cells were grown on the 4.2 cm glass coverslip coated with collagen IV / Poly-D-lysine, and then locked in a round metal microscopy chamber using 1 mm thick silicon gaskets, upper coverslip and air-tight screwing lock. In such a chamber the original air conditions (21% or 0% O2) were preserved during the course of microscopy (5-10 min).

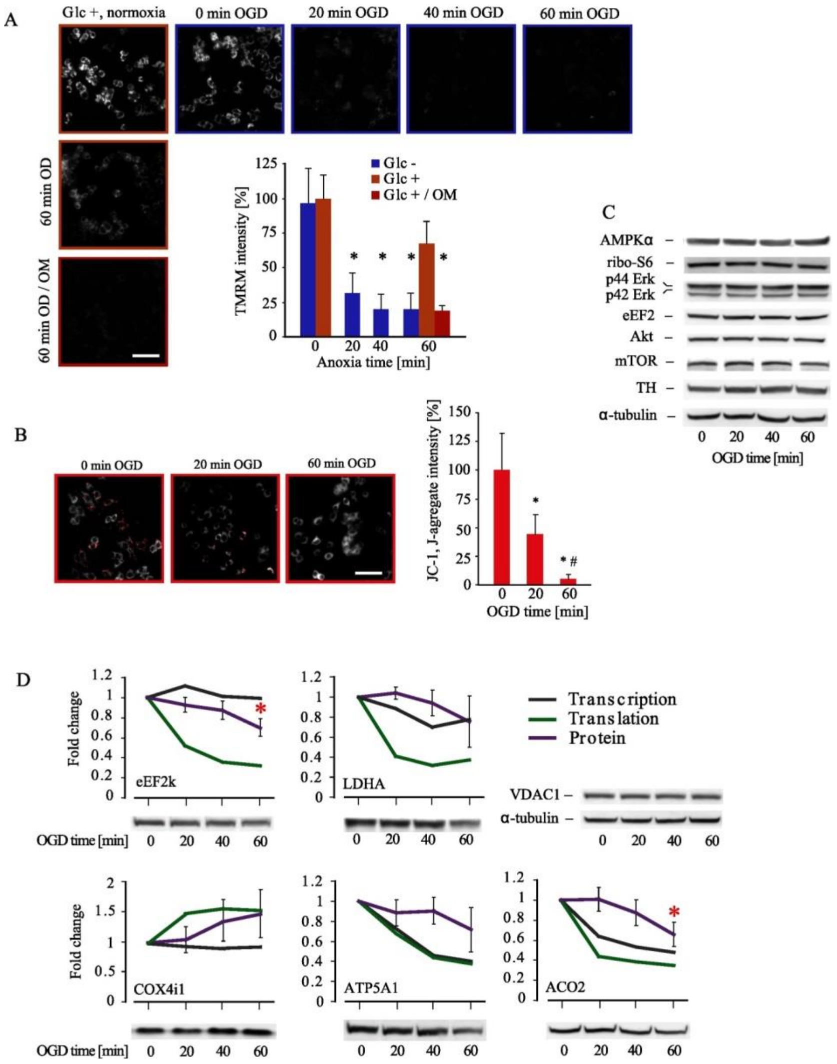

Additional Figure A2. Analysis of mitochondrial membrane potential and protein levels under OGD.

(A) Changes in TMRM probe intensity under OGD and OD conditions. Without glucose TMRM fluorescence significantly decreases, in contrast to Glc + samples. Treatment with oligomycin (OM, 10 µM) causes the $\Delta\Psi$m depolarisation under OD, suggesting that in Glc + cells F1Fo ATP synthase is capable to maintain mitochondrial polarisation, working in reverse mode and utilising glycolytic ATP. **(B)** Decrease in JC-1 probe fluorescence (J-aggregated form, red) under OGD confirms a prominent mitochondrial depolarisation. In the microscopy images bar is 50 µm. Error bars demonstrate a range in TMRM and J-aggregate intensities, calculated as SD for 40-50 cells (in total) in 4 randomly selected fields of view. **(C)** Western blotting demonstrate no changes in the total levels of proteins, subjected to phosphorylation analysis (see Fig 1D). The level of tyrosine hydroxylase (TH), one of the major functional markers of PC12 cells, also was not affected by OGD. **(D)** Trajectories of RNA, translation and protein levels for individual genes assessed with ribosome profiling and Western blotting. Voltage-dependent anion channel 1 (VDAC1) and α-tubulin are used as loading controls for the mitochondrial and total cellular proteins. Ribosome profiling data are averaged from two replicates and Western blotting data are in triplicates, error bars represent standard deviation. Asterisks indicate significant difference from control sample (0 min OGD). A hash in the panel **B** indicates significant difference from 20 min OGD.

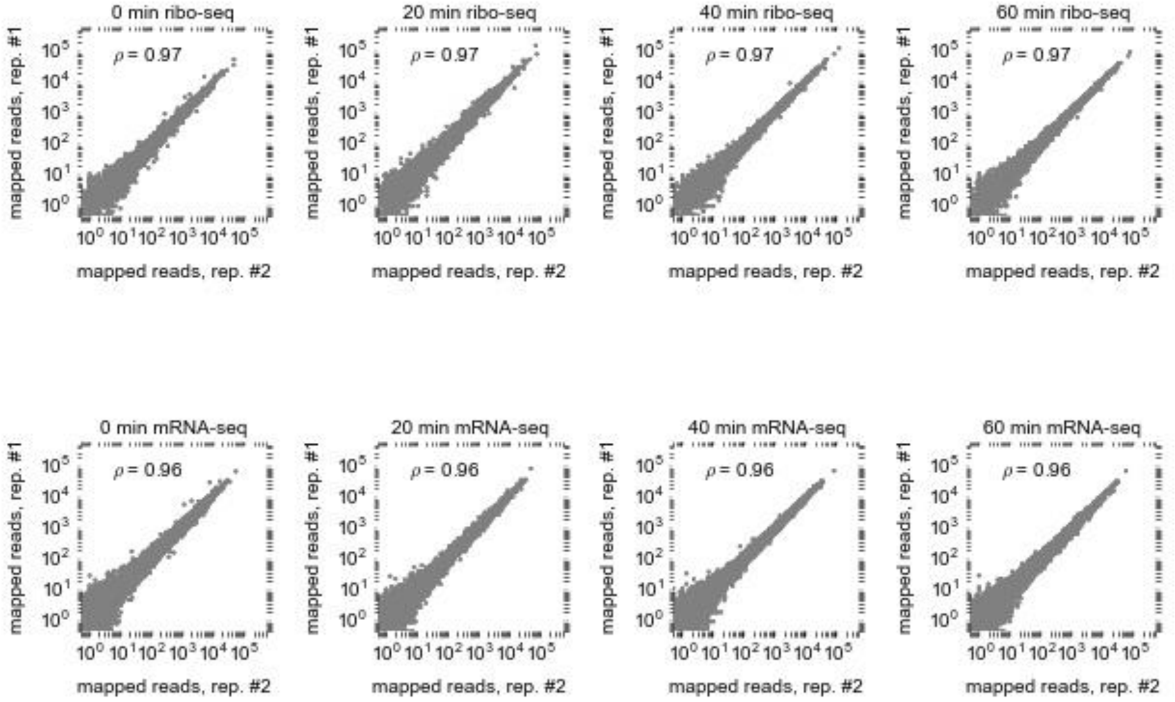

Additional Figure A3. Reproducibility of biological replicas.

Scatter plot represents the number of raw ribosome protected fragments aligning to each gene between replicas for four different time points (from left to right), and ribo-seq are at the top and mRNA-seq are at the bottom. The Spearman coefficients are indicated for each plot.

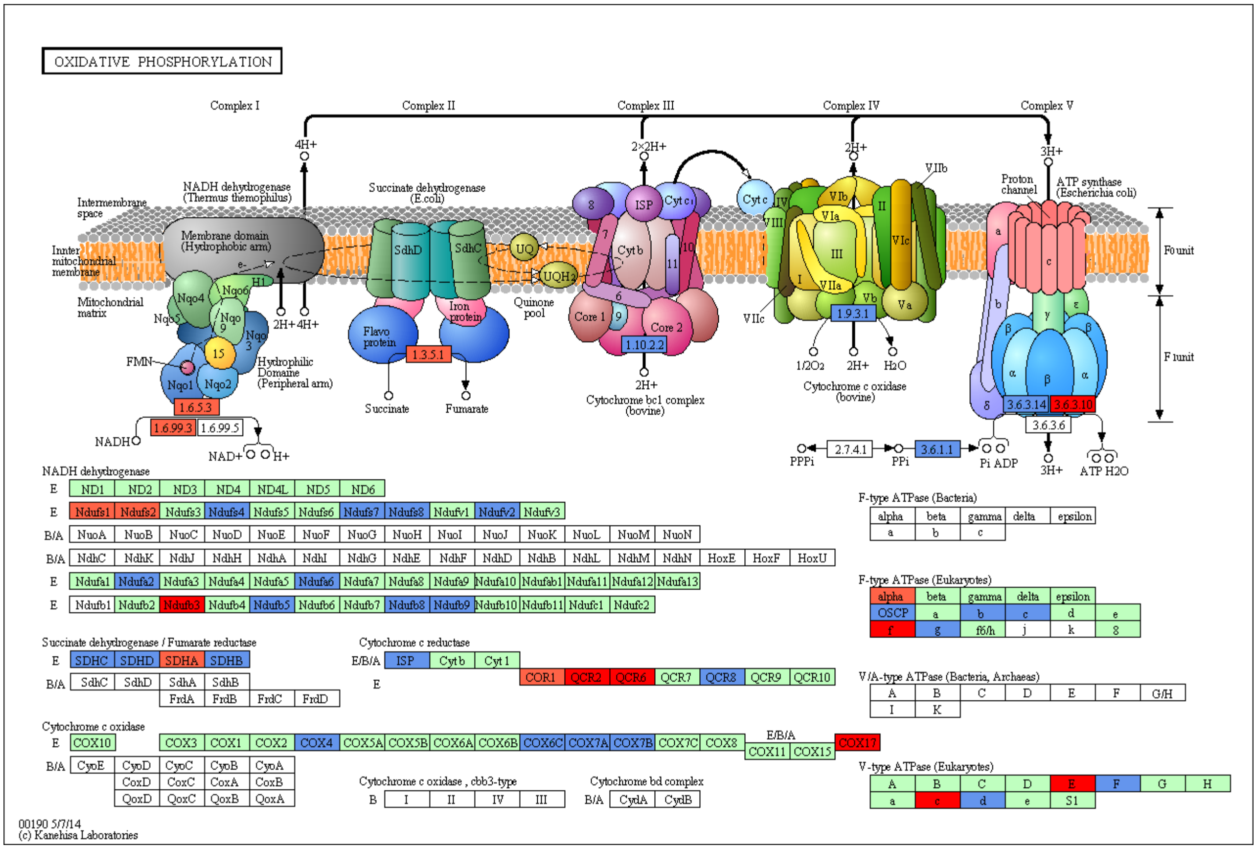

Additional Figure A4. DE genes involved in oxidative phosphorylation.

KEGG oxidative phosphorylation pathway is shown. Genes and enzymatic processes present in Rat are highlighted green, those found to be transcriptionally downregulated are red while others found to DE increased and decreased at a ribo-seq level are colored blue and tomato respectively.

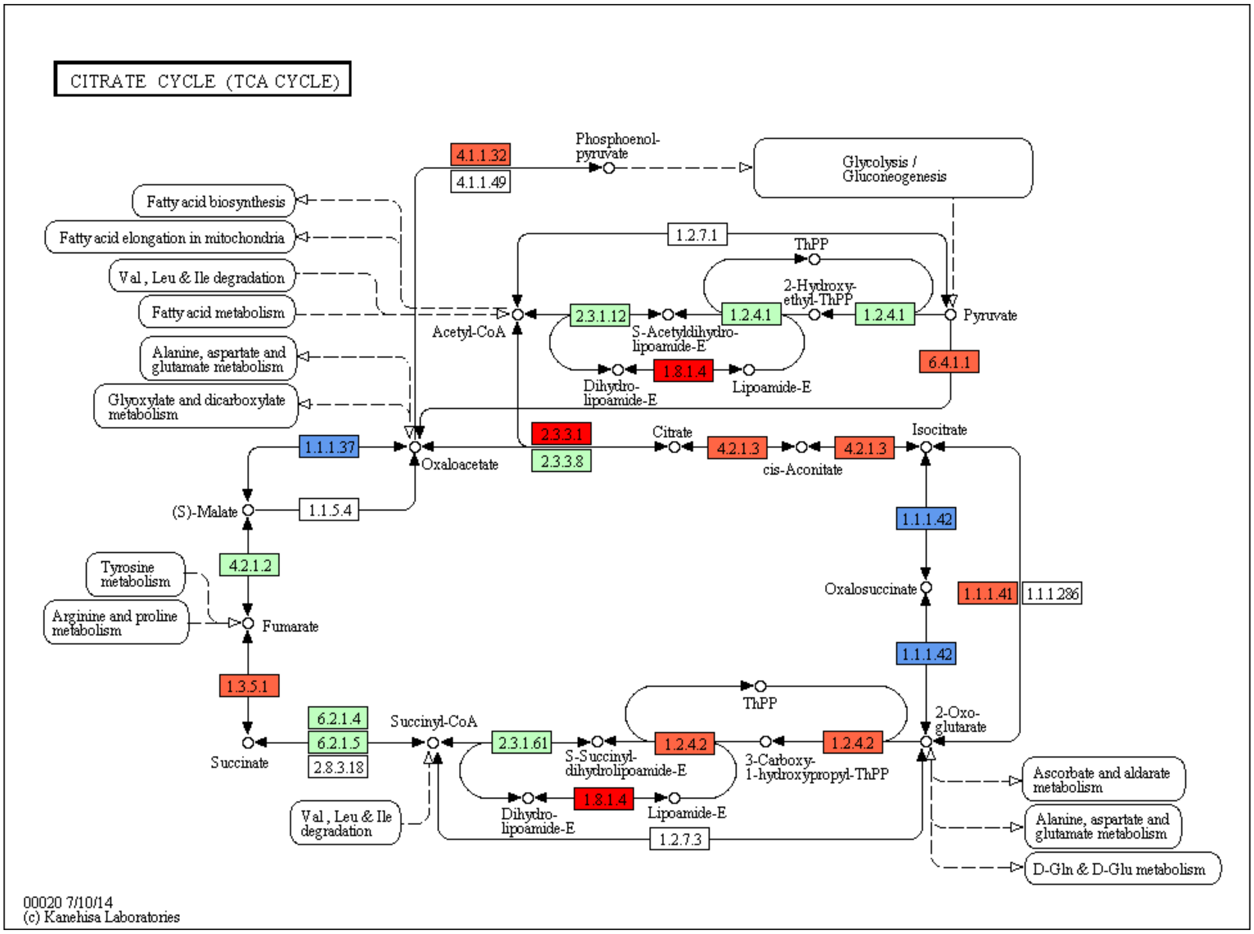

Additional Figure A5. DE genes involved in TCA cycle.

KEGG TCA cycle is shown. Genes and enzymatic processes present in Rat are highlighted green, those found to be transcriptionally downregulated are red while others found to DE increased and decreased at a ribo-seq level are colored blue and tomato respectively.

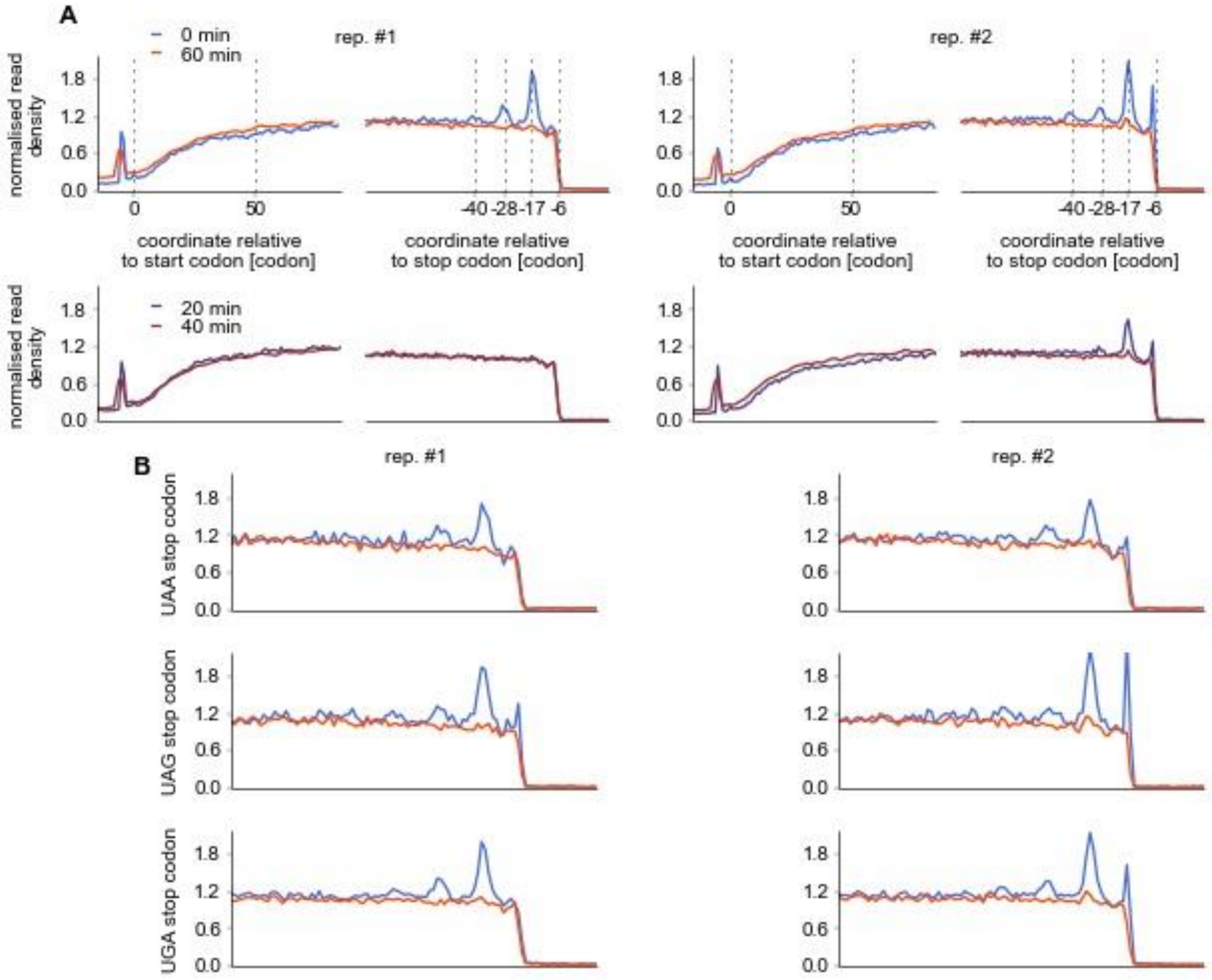

Additional Figure A6. The effect of OGD on ribosome queueing at upstream of stop codons.

(A) Metagene profiles of ribosome densities at 0, 20, 40 and 60 min of OGD for both replicas. The normalised profiles of a minimum of 5,000 mRNAs were used to produce these profiles **(B)** Metagene profiles of ribosome densities grouped based on the identity of stop codons.

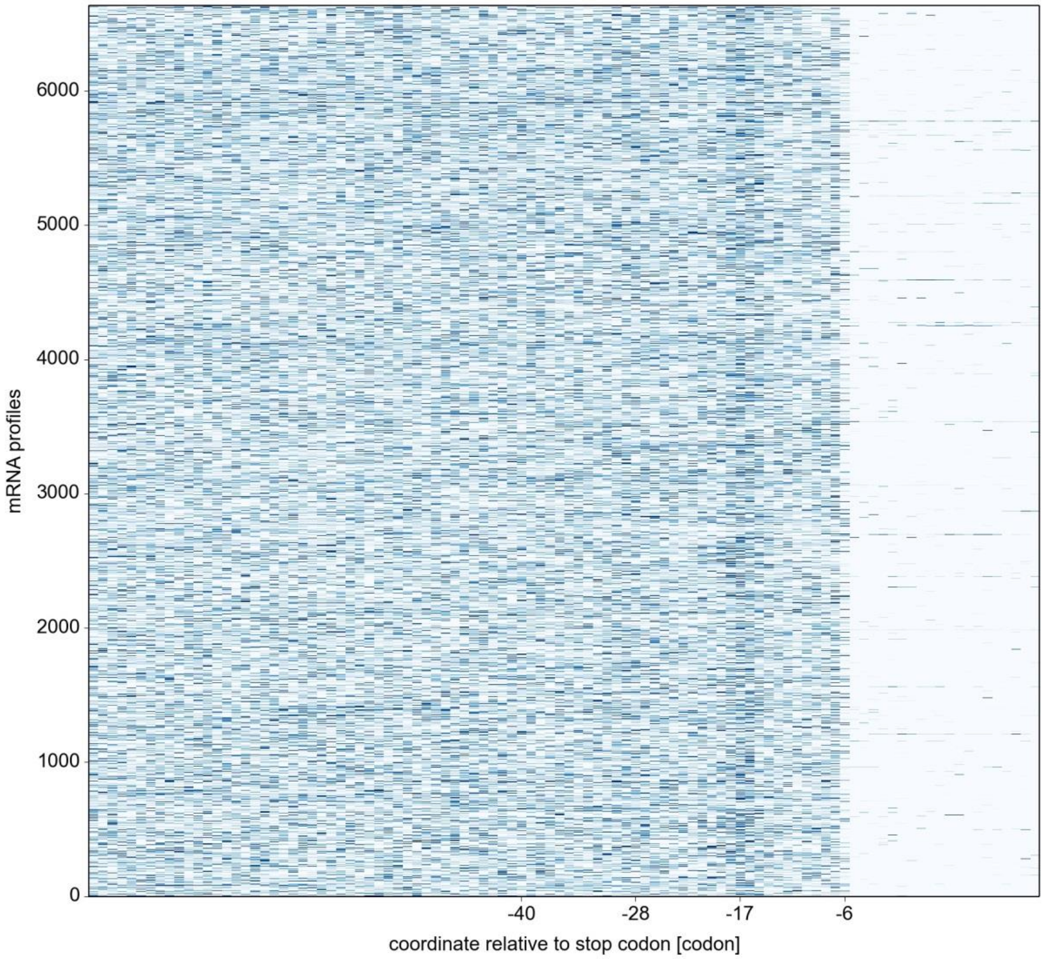

Additional Figure A7. Ribosome queueing appears to be widespread in highly translated gene under normoxia.

Individual mRNA profiles of ribosome density (5'end of reads) of mRNAs at 0 min. Reads from both replicates were aggregated to increase read density. Note periodic increased density at the distances with an interval cooresponding to the length of the ribosome footprint.

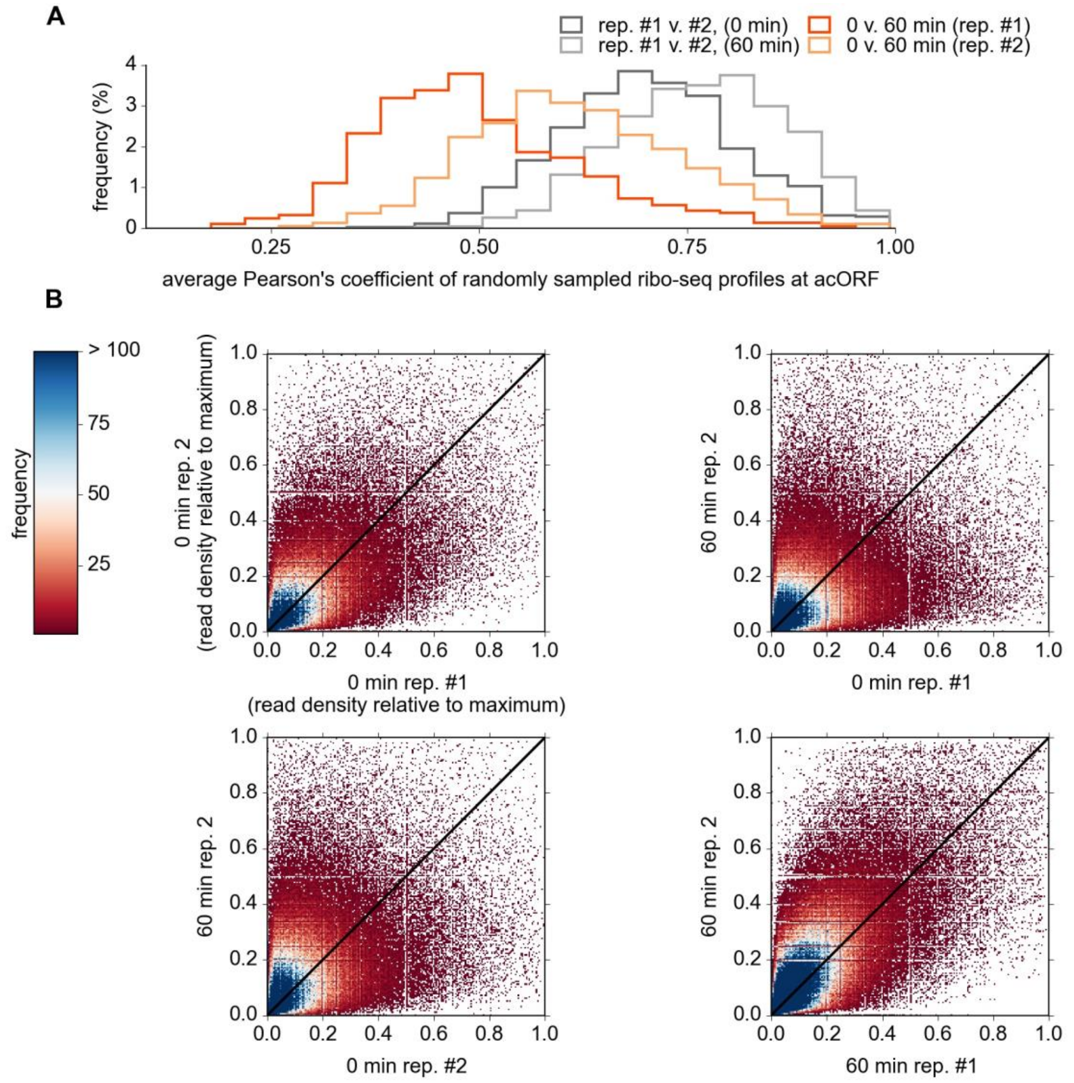

Additional Figure A8 Widespread changes of ribo-seq read density during OGD

(A) Distribution of the average Pearson's correlations of ribosome densities for individual mRNA profiles between replicas and across conditions after random sampling of profiles such that both profiles in pairwise comparison contain same read density. **(B)** 2 dimensional histogram displaying an aggregation of correlations between replicas and across conditions for mRNAs that contributed to Fig. 3E

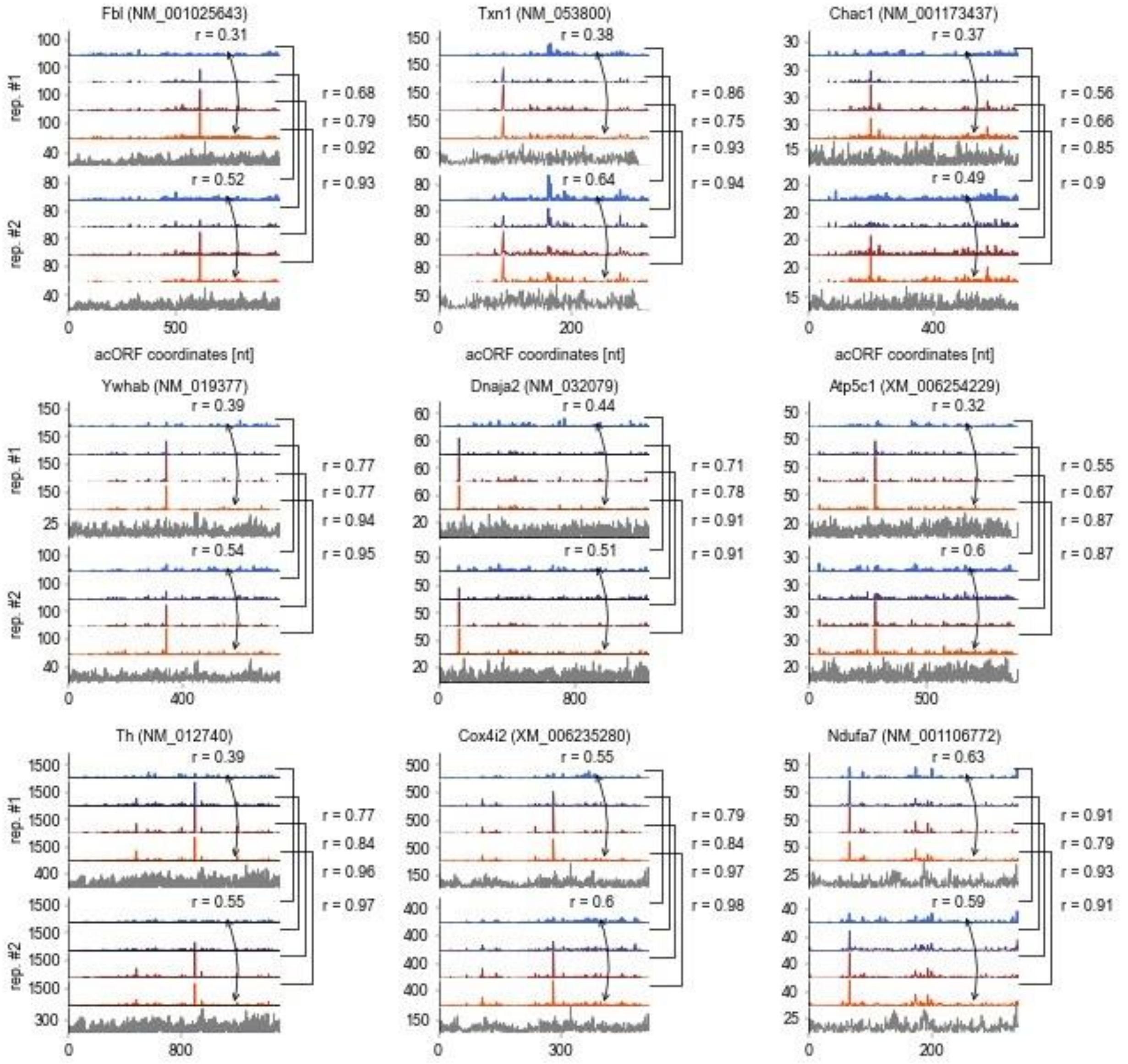

Additional Figure A9. Examples of mRNAs with OGD induced ribosome pauses.

Pearson correlation coefficients are indicated for the profiles obtained from independent experiments and between different time points of OGD. Standard gene symbols and RefSeq accession numbers for each mRNA sequence are indicated.

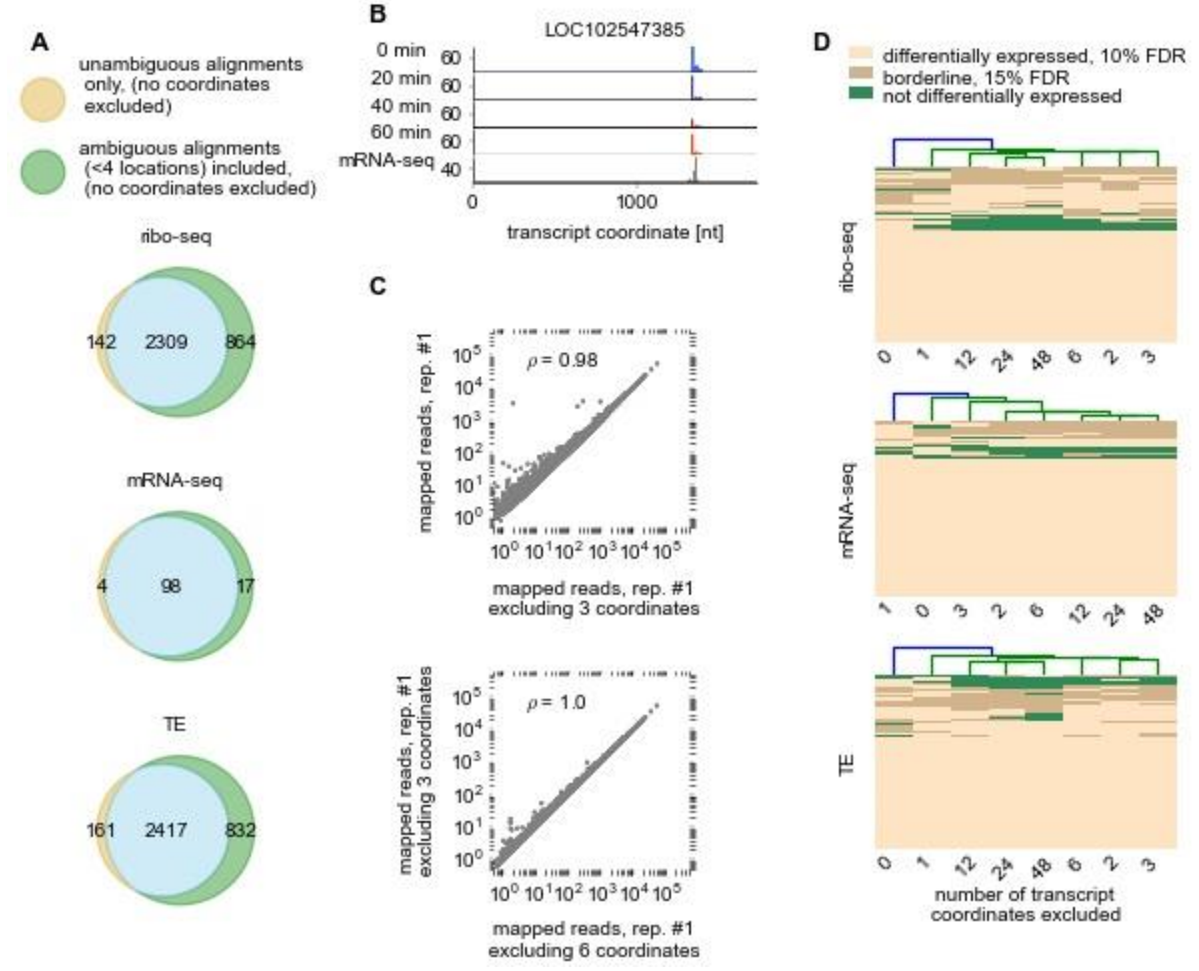

Additional Figure A10. The effect of ambiguously aligned reads and high peaks of density on detection of differentially expressed genes.

(A) Number of differentially expressed genes identified based on ribo-seq, mRNA-seq and TE after 60 min of OGD with or without the weighted inclusion of ambiguous alignments. **(B)** An example of a gene spuriously identified as differentially expressed. Ambiguously mapped reads represent the genes level of expression inaccurately. **(C)** The effect of highest density peaks exclusion on correlations of replicate number 1. Spearman correlation coefficient is indicated. **(D)** The effect of highest density peaks exclusion on differential gene expression detection.
