# Supplementary material for: Oxygen and glucose deprivation induces widespread alterations in mRNA translation within 20 minutes
Source: Genome Biol. 2015 May 6;16(1):90. doi: 10.1186/s13059-015-0651-z (PMC4419486; doi:10.1186/s13059-015-0651-z)
Supplement: Additional file 2: — This file contains a mini web site with additional ribosome profiles of individual mRNAs that are mentioned in the manuscript. The same mini web site is available at http://lapti.ucc.ie/ogd/. [file 13059_2015_651_MOESM2_ESM.zip › ogd/index.html]

# Oxygen and glucose deprivation induces widespread alterations in mRNA translation within 20 minutes

## uORF translation, AUG start

| Gene symbol | RefSeq ID |
| Atp13a2 | NM\_001173432.1 |
| Bok | NM\_017312.2 |
| Ccdc92 | XM\_006249292.1 |
| Cenpq | NM\_001014215.1 |
| Eif5 | XM\_006240608.1 |
| Fam53a | XM\_006251262.1 |
| Ip6k1 | XM\_006243839.1 |
| LOC691153 | NM\_001109627.1 |
| Lppr2 | XM\_006242628.1 |
| Maf1 | NM\_001014085.1 |
| Mief1 | NM\_001007709.1 |
| Mmd | NM\_001007673.1 |
| Pold4 | XM\_006230841.1 |
| RGD1560212 | NM\_001077676.1 |
| Rpp14 | NM\_001108372.2 |
| Scarb1 | NM\_031541.1 |
| Slc27a4 | NM\_001100706.1 |
| Slc39a11 | XM\_006247668.1 |
| Syt9 | XM\_006229961.1 |
| Tbpl1 | NM\_001127201.1 |
| Tmem107 | NM\_001109648.1 |
| Trpm7 | NM\_053705.2 |
| Zdhhc8 | NM\_001039021.1 |
| Zfp133 | NM\_001024313.1 |

## uORF translation, CUG start

| Gene symbol | RefSeq ID |
| Anp32e | NM\_001013200.1 |
| Arfrp1 | XM\_006235718.1 |
| Cep19 | NM\_138865.1 |
| Csk | XM\_006243163.1 |
| Dclre1b | XM\_006233080.1 |
| Dnajc19 | XM\_006232259.1 |
| Dynll2 | NM\_080697.2 |
| Eef2k | XM\_006230156.1 |
| Eif1a | XM\_006254688.1 |
| Eif1ad | NM\_001008305.1 |
| Fam220a | NM\_001017485.1 |
| Fut1 | NM\_031236.1 |
| Junb | NM\_021836.2 |
| LOC361346 | XM\_006254927.1 |
| Lrrfip2 | XM\_006243960.1 |
| Lsm2 | NM\_001165922.1 |
| Mgat4b | NM\_001127533.1 |
| Nfyb | NM\_031553.2 |
| Pkmyt1 | NM\_001105766.1 |
| RGD1564400 | XM\_001058193.3 |
| Rpl39l | NM\_001195471.1 |
| Srd5a1 | NM\_017070.3 |
| Taok2 | XM\_006230331.1 |
| Tmem230 | NM\_001048043.1 |
| Wbp5 | NM\_001127502.1 |
| Zfp428 | XM\_006228447.1 |

## uORF translation, start site not known

| Gene symbol | RefSeq ID |
| Akr1a1 | NM\_031000.3 |
| Bbs9 | XM\_001074631.4 |
| Cdc42se1 | NM\_001039044.1 |
| Cks2 | NM\_001126083.1 |
| Cnppd1 | NM\_199112.1 |
| Dlk1 | NM\_053744.1 |
| Eif1ax | NM\_001106963.1 |
| Eif4g2 | NM\_001017374.2 |
| Grb2 | NM\_030846.2 |
| Map3k9 | XM\_006240303.1 |
| Polr2m | NM\_183402.1 |
| Rab34 | NM\_001012140.1 |
| Tatdn2 | NM\_001109252.1 |
| Tor1aip2 | NM\_001165897.1 |
| Vegfa | NM\_001110333.2 |
| Zfp706 | NM\_001126087.1 |
| Zfp868 | XM\_006252916.1 |

## uORF translation, multiple uORFs

| Gene symbol | RefSeq ID |
| Jun | NM\_021835.3 |
| LOC100911699 | XM\_003751181.2 |
| Ubl3 | NM\_001015030.1 |

## frameshifting/dual coding

| Gene symbol | RefSeq ID |
| Amn | XM\_006240588.1 |
| Romo1 | NM\_001195490.1 |
| RGD1307752 | NM\_001013922.1 |

## N-terminal extension

| Gene symbol | RefSeq ID |
| Adm | NM\_012715.1 |
| Bcl2l11 | XM\_006234986.1 |
| Fam178b | XM\_006244842.1 |
| Ppp1r2 | NM\_138823.2 |
| Ptms | NM\_031975.2 |

## stop codon readthrough

| Gene symbol | RefSeq ID |
| Fkbp1a | NM\_013102.3 |
| Hadhb | NM\_133618.2 |
| Hs1bp3 | XM\_233975.7 |
| Klc1 | NM\_001081974.1 |
| LOC102554884 | XM\_006240612.1 |
| Mdh1 | NM\_033235.1 |
| Mrto4 | XM\_006239178.1 |
| Nedd8 | NM\_138878.2 |
| Nudcd2 | NM\_001009621.2 |
| Plat | NM\_013151.2 |
| Polr2l | NM\_001143911.1 |
| Ppp4c | XM\_006230206.1 |
| Rfc2 | NM\_053786.1 |
| Rnf111 | XM\_006243373.1 |
| Sec13 | NM\_001006978.1 |
| Slc7a1 | NM\_013111.2 |
| Ssna1 | XM\_006233613.1 |
| Thy1 | NM\_012673.2 |

## bicistronic

| Gene symbol | RefSeq ID |
| Eif1b | NM\_001106867.1 |
| Mien1 | NM\_001108296.1 |
| Ost4 | NM\_001134691.1 |
| RGD1565784 | NM\_001109028.1 |
| Rpp14 | NM\_001108372.2 |
| Ubl3 | NM\_001015030.1 |
