# Supplementary figures and images for: Oxygen and glucose deprivation induces widespread alterations in mRNA translation within 20 minutes (part 2 of 2)
Source: Genome Biol. 2015 May 6;16(1):90. doi: 10.1186/s13059-015-0651-z (PMC4419486; doi:10.1186/s13059-015-0651-z)

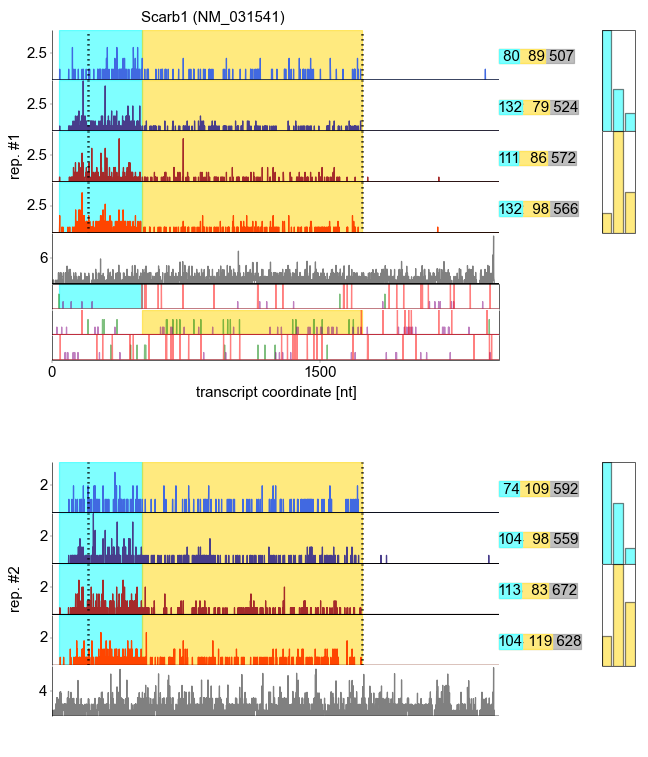

Supplement: Additional file 2: — This file contains a mini web site with additional ribosome profiles of individual mRNAs that are mentioned in the manuscript. The same mini web site is available at http://lapti.ucc.ie/ogd/. [file 13059_2015_651_MOESM2_ESM.zip › ogd/profiles/NM_031541.png]
