# Supplementary figures and images for: Oxygen and glucose deprivation induces widespread alterations in mRNA translation within 20 minutes (part 1 of 2)
Source: Genome Biol. 2015 May 6;16(1):90. doi: 10.1186/s13059-015-0651-z (PMC4419486; doi:10.1186/s13059-015-0651-z)

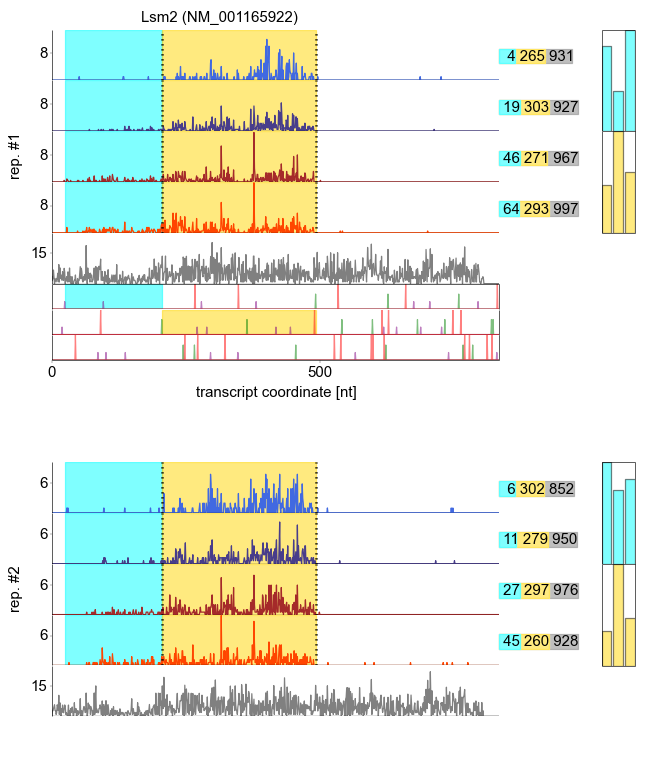

Supplement: Additional file 2: — This file contains a mini web site with additional ribosome profiles of individual mRNAs that are mentioned in the manuscript. The same mini web site is available at http://lapti.ucc.ie/ogd/. [file 13059_2015_651_MOESM2_ESM.zip › ogd/profiles/NM_001165922.png]

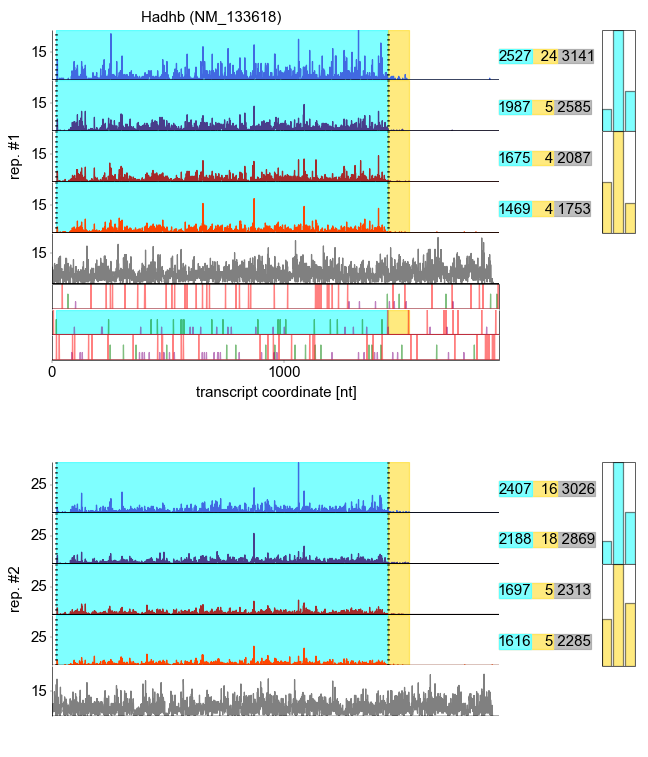

Supplement: Additional file 2: — This file contains a mini web site with additional ribosome profiles of individual mRNAs that are mentioned in the manuscript. The same mini web site is available at http://lapti.ucc.ie/ogd/. [file 13059_2015_651_MOESM2_ESM.zip › ogd/profiles/NM_133618.png]

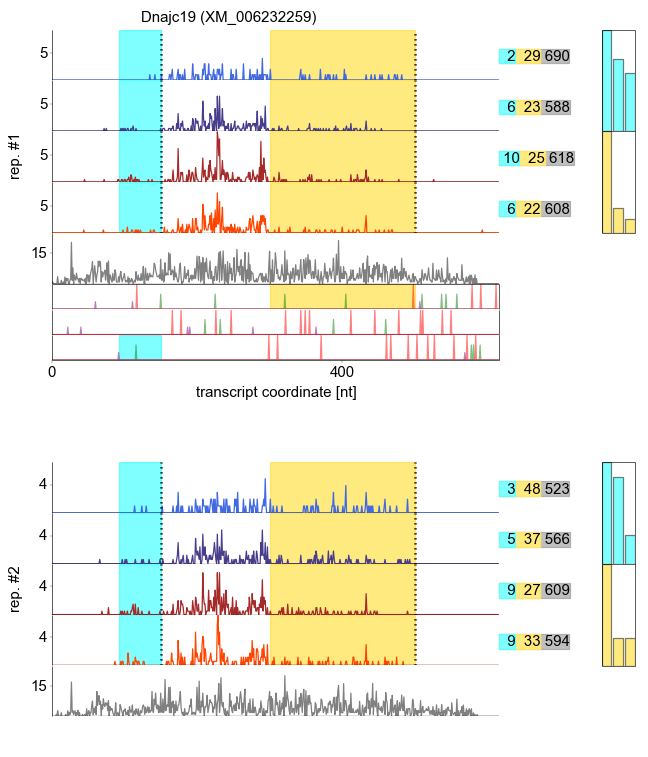

Supplement: Additional file 2: — This file contains a mini web site with additional ribosome profiles of individual mRNAs that are mentioned in the manuscript. The same mini web site is available at http://lapti.ucc.ie/ogd/. [file 13059_2015_651_MOESM2_ESM.zip › ogd/profiles/XM_006232259.png]

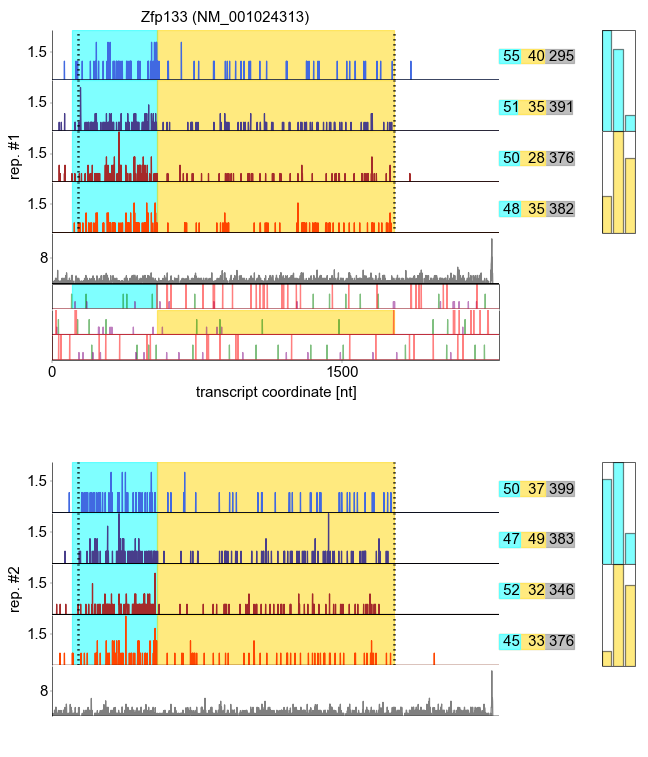

Supplement: Additional file 2: — This file contains a mini web site with additional ribosome profiles of individual mRNAs that are mentioned in the manuscript. The same mini web site is available at http://lapti.ucc.ie/ogd/. [file 13059_2015_651_MOESM2_ESM.zip › ogd/profiles/NM_001024313.png]

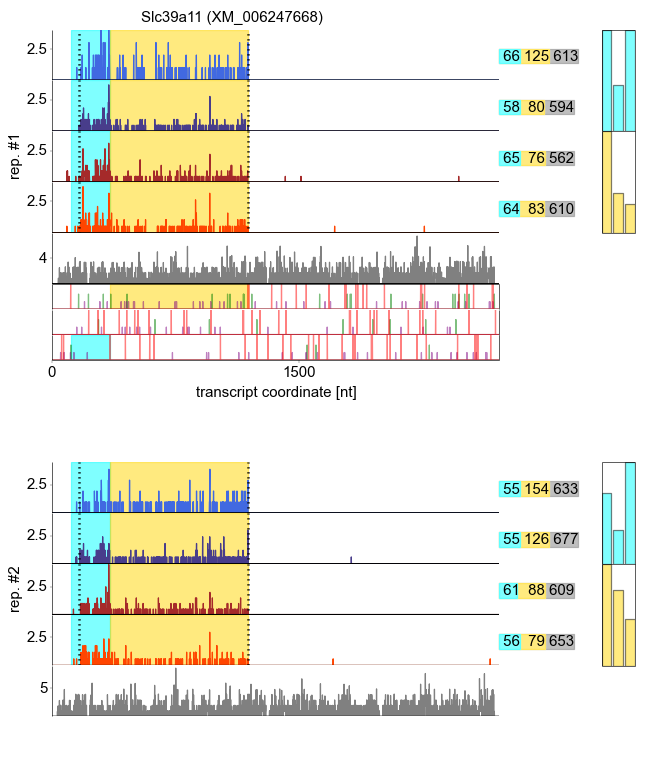

Supplement: Additional file 2: — This file contains a mini web site with additional ribosome profiles of individual mRNAs that are mentioned in the manuscript. The same mini web site is available at http://lapti.ucc.ie/ogd/. [file 13059_2015_651_MOESM2_ESM.zip › ogd/profiles/XM_006247668.png]

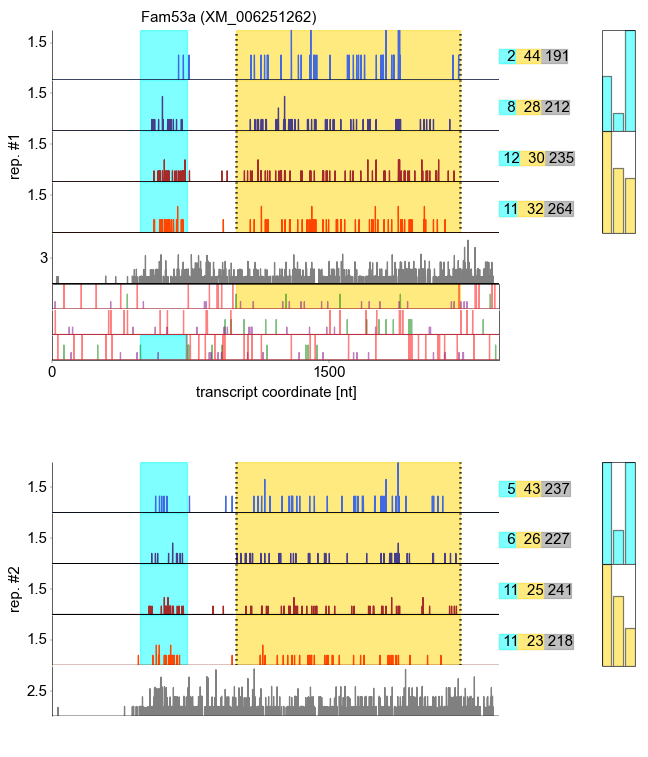

Supplement: Additional file 2: — This file contains a mini web site with additional ribosome profiles of individual mRNAs that are mentioned in the manuscript. The same mini web site is available at http://lapti.ucc.ie/ogd/. [file 13059_2015_651_MOESM2_ESM.zip › ogd/profiles/XM_006251262.png]

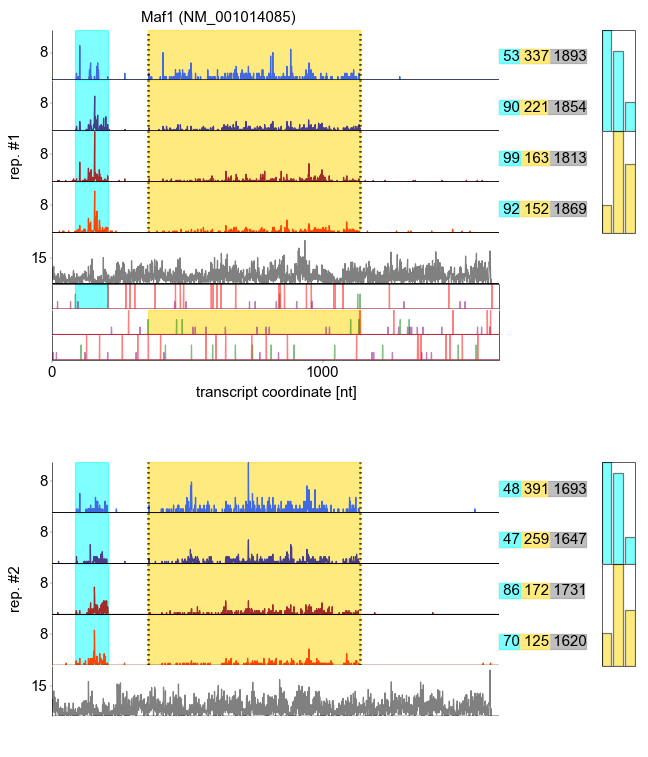

Supplement: Additional file 2: — This file contains a mini web site with additional ribosome profiles of individual mRNAs that are mentioned in the manuscript. The same mini web site is available at http://lapti.ucc.ie/ogd/. [file 13059_2015_651_MOESM2_ESM.zip › ogd/profiles/NM_001014085.png]

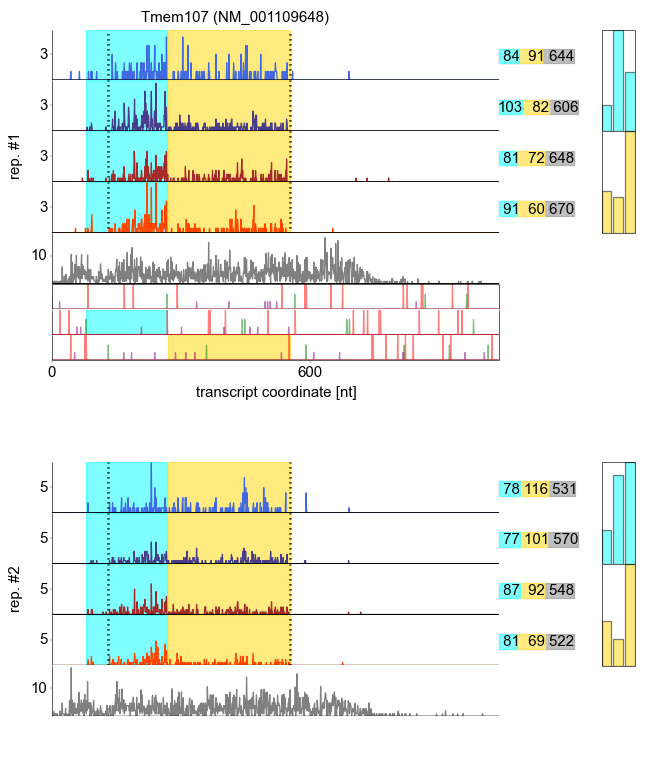

Supplement: Additional file 2: — This file contains a mini web site with additional ribosome profiles of individual mRNAs that are mentioned in the manuscript. The same mini web site is available at http://lapti.ucc.ie/ogd/. [file 13059_2015_651_MOESM2_ESM.zip › ogd/profiles/NM_001109648.png]

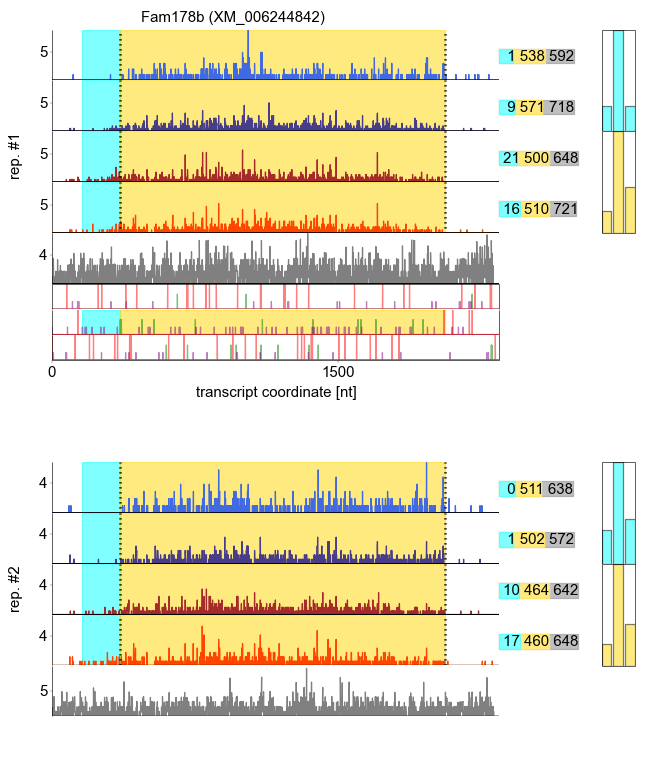

Supplement: Additional file 2: — This file contains a mini web site with additional ribosome profiles of individual mRNAs that are mentioned in the manuscript. The same mini web site is available at http://lapti.ucc.ie/ogd/. [file 13059_2015_651_MOESM2_ESM.zip › ogd/profiles/XM_006244842.png]

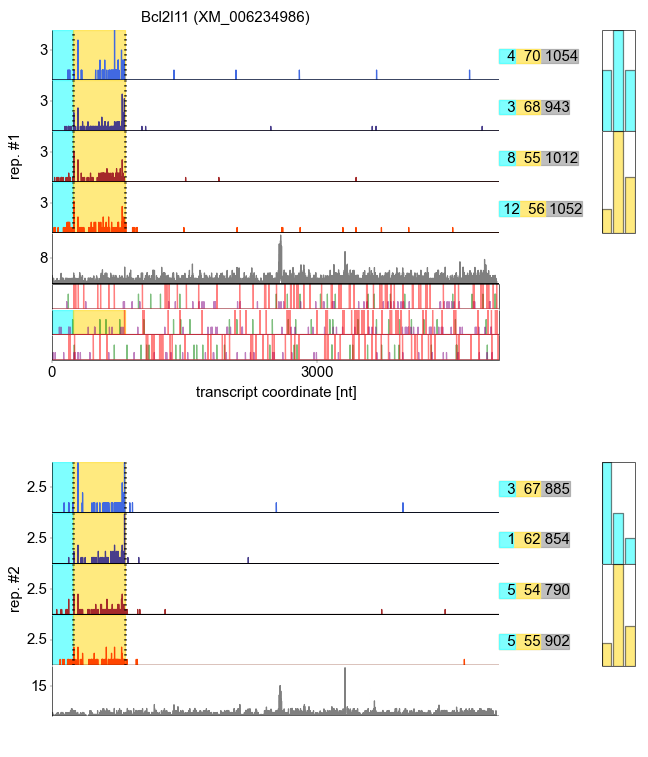

Supplement: Additional file 2: — This file contains a mini web site with additional ribosome profiles of individual mRNAs that are mentioned in the manuscript. The same mini web site is available at http://lapti.ucc.ie/ogd/. [file 13059_2015_651_MOESM2_ESM.zip › ogd/profiles/XM_006234986.png]

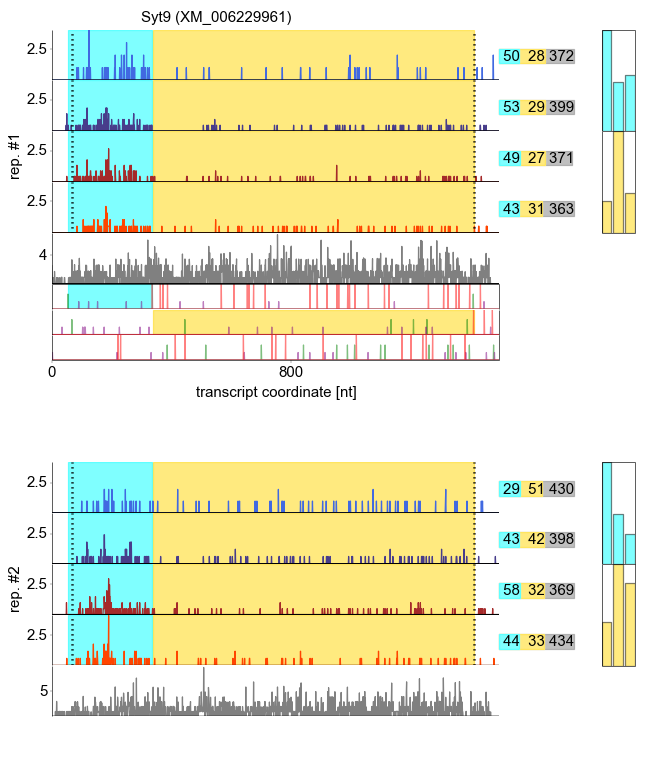

Supplement: Additional file 2: — This file contains a mini web site with additional ribosome profiles of individual mRNAs that are mentioned in the manuscript. The same mini web site is available at http://lapti.ucc.ie/ogd/. [file 13059_2015_651_MOESM2_ESM.zip › ogd/profiles/XM_006229961.png]

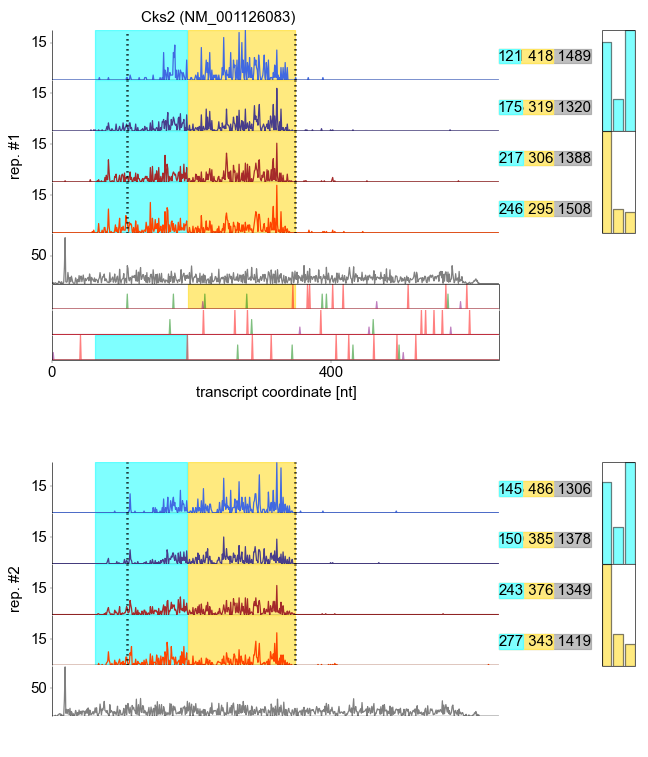

Supplement: Additional file 2: — This file contains a mini web site with additional ribosome profiles of individual mRNAs that are mentioned in the manuscript. The same mini web site is available at http://lapti.ucc.ie/ogd/. [file 13059_2015_651_MOESM2_ESM.zip › ogd/profiles/NM_001126083.png]

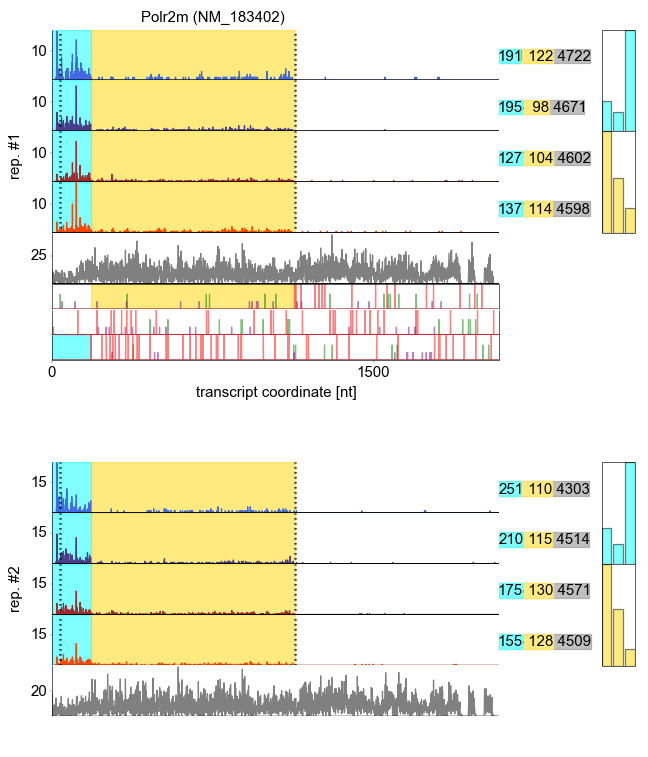

Supplement: Additional file 2: — This file contains a mini web site with additional ribosome profiles of individual mRNAs that are mentioned in the manuscript. The same mini web site is available at http://lapti.ucc.ie/ogd/. [file 13059_2015_651_MOESM2_ESM.zip › ogd/profiles/NM_183402.png]

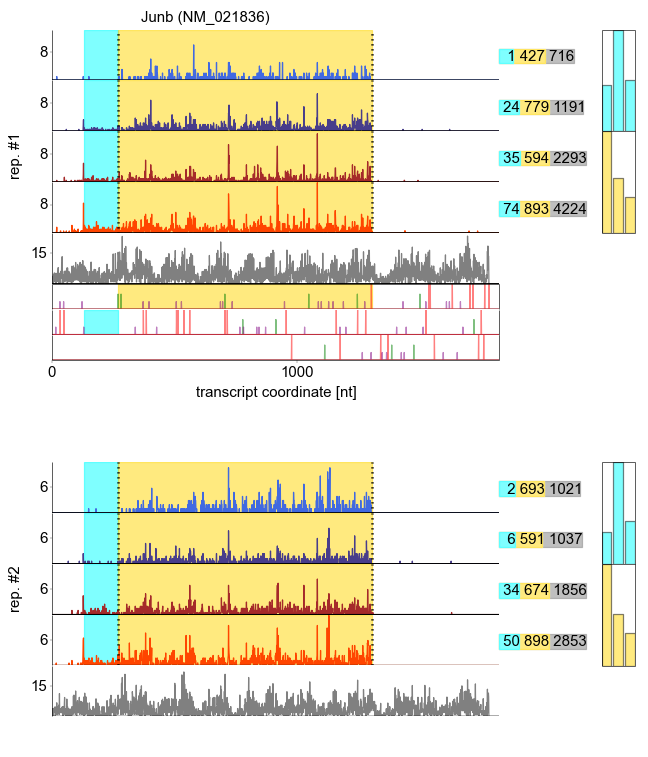

Supplement: Additional file 2: — This file contains a mini web site with additional ribosome profiles of individual mRNAs that are mentioned in the manuscript. The same mini web site is available at http://lapti.ucc.ie/ogd/. [file 13059_2015_651_MOESM2_ESM.zip › ogd/profiles/NM_021836.png]

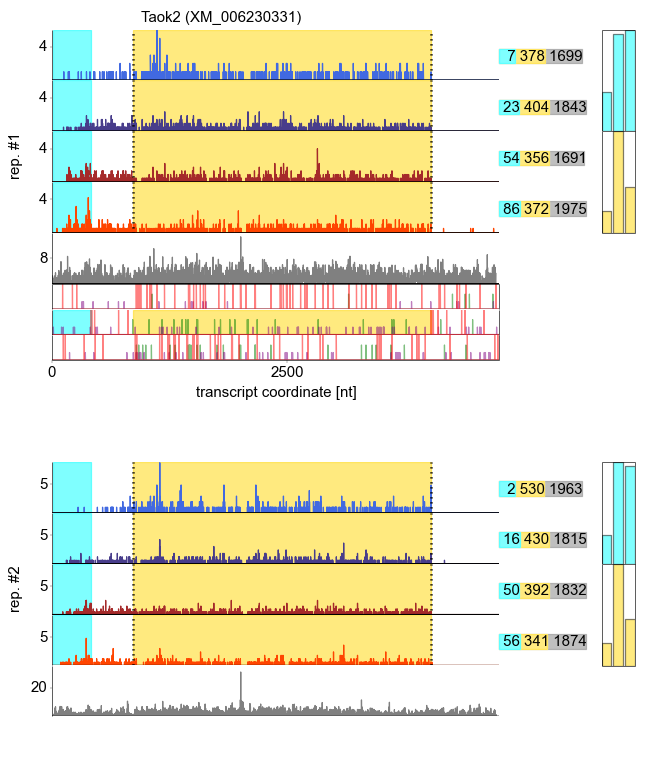

Supplement: Additional file 2: — This file contains a mini web site with additional ribosome profiles of individual mRNAs that are mentioned in the manuscript. The same mini web site is available at http://lapti.ucc.ie/ogd/. [file 13059_2015_651_MOESM2_ESM.zip › ogd/profiles/XM_006230331.png]

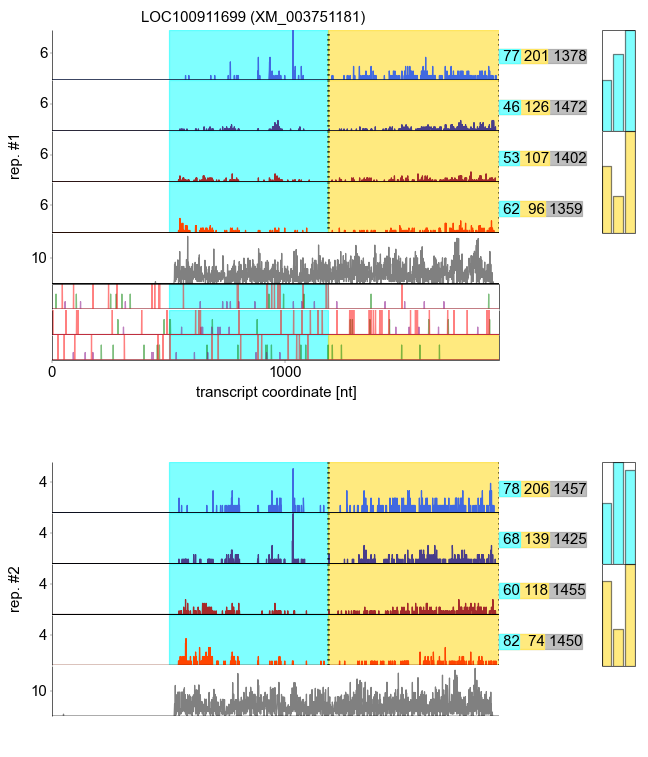

Supplement: Additional file 2: — This file contains a mini web site with additional ribosome profiles of individual mRNAs that are mentioned in the manuscript. The same mini web site is available at http://lapti.ucc.ie/ogd/. [file 13059_2015_651_MOESM2_ESM.zip › ogd/profiles/XM_003751181.png]

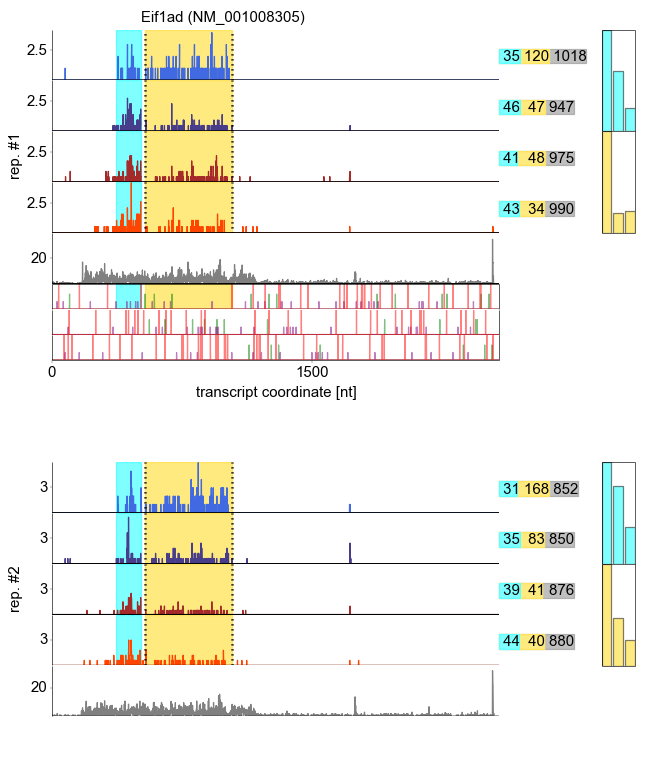

Supplement: Additional file 2: — This file contains a mini web site with additional ribosome profiles of individual mRNAs that are mentioned in the manuscript. The same mini web site is available at http://lapti.ucc.ie/ogd/. [file 13059_2015_651_MOESM2_ESM.zip › ogd/profiles/NM_001008305.png]

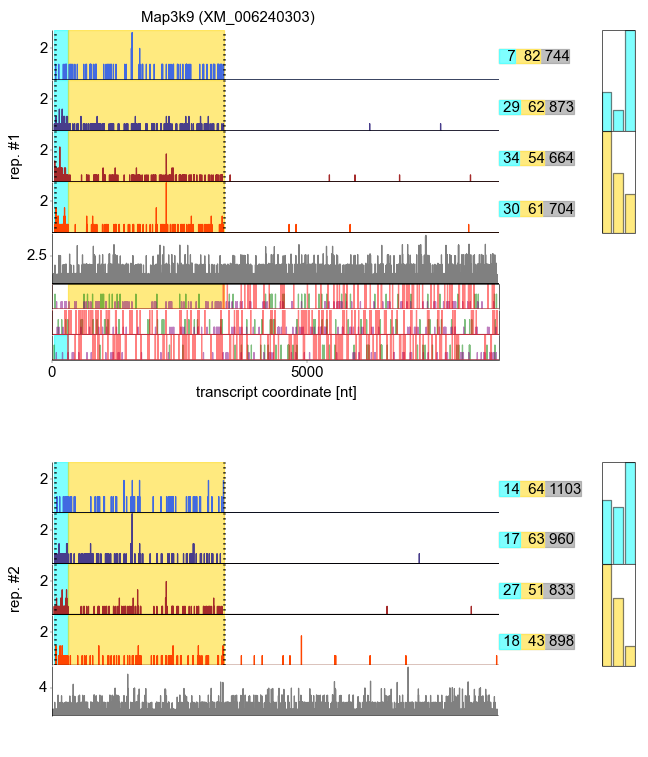

Supplement: Additional file 2: — This file contains a mini web site with additional ribosome profiles of individual mRNAs that are mentioned in the manuscript. The same mini web site is available at http://lapti.ucc.ie/ogd/. [file 13059_2015_651_MOESM2_ESM.zip › ogd/profiles/XM_006240303.png]

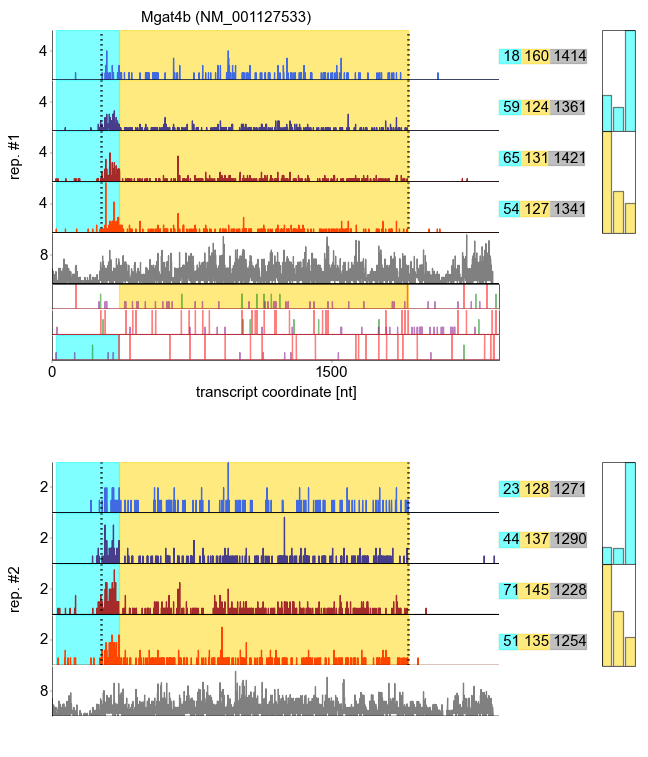

Supplement: Additional file 2: — This file contains a mini web site with additional ribosome profiles of individual mRNAs that are mentioned in the manuscript. The same mini web site is available at http://lapti.ucc.ie/ogd/. [file 13059_2015_651_MOESM2_ESM.zip › ogd/profiles/NM_001127533.png]

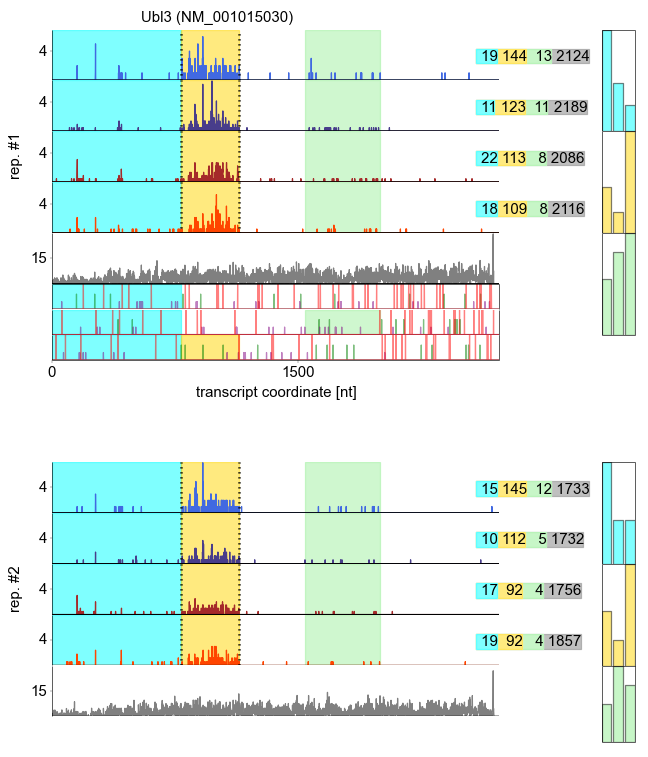

Supplement: Additional file 2: — This file contains a mini web site with additional ribosome profiles of individual mRNAs that are mentioned in the manuscript. The same mini web site is available at http://lapti.ucc.ie/ogd/. [file 13059_2015_651_MOESM2_ESM.zip › ogd/profiles/NM_001015030.png]

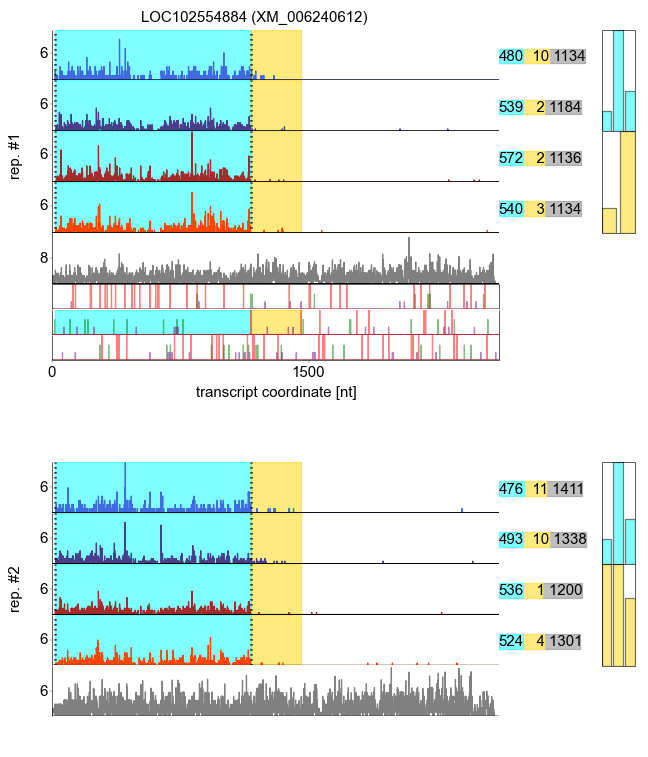

Supplement: Additional file 2: — This file contains a mini web site with additional ribosome profiles of individual mRNAs that are mentioned in the manuscript. The same mini web site is available at http://lapti.ucc.ie/ogd/. [file 13059_2015_651_MOESM2_ESM.zip › ogd/profiles/XM_006240612.png]

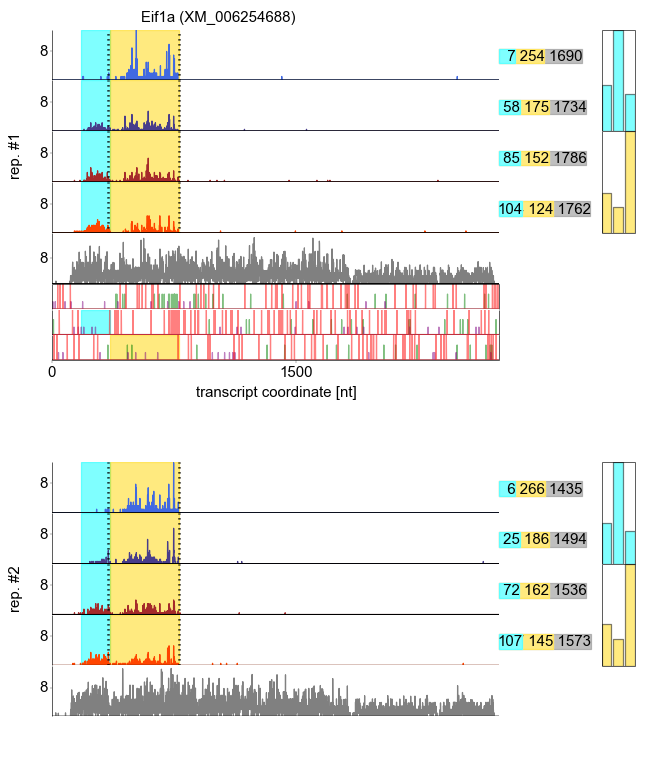

Supplement: Additional file 2: — This file contains a mini web site with additional ribosome profiles of individual mRNAs that are mentioned in the manuscript. The same mini web site is available at http://lapti.ucc.ie/ogd/. [file 13059_2015_651_MOESM2_ESM.zip › ogd/profiles/XM_006254688.png]

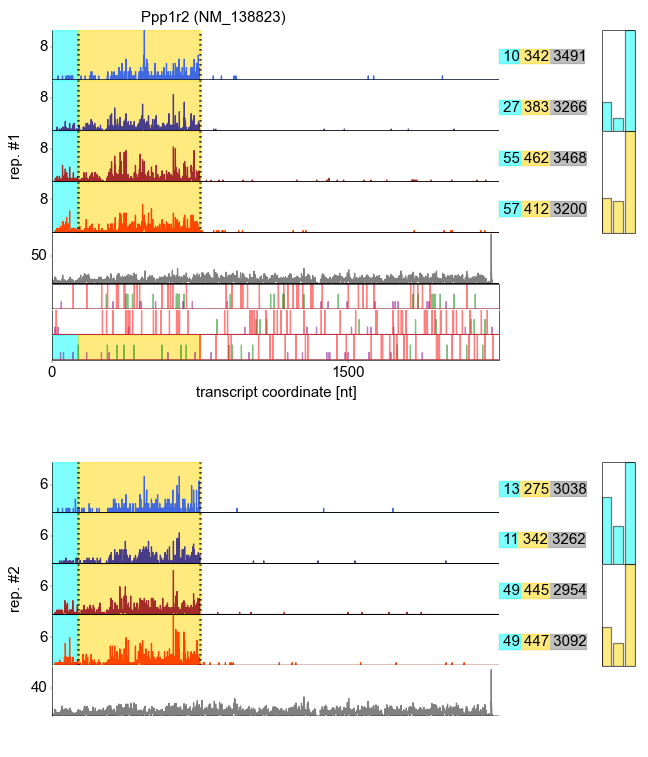

Supplement: Additional file 2: — This file contains a mini web site with additional ribosome profiles of individual mRNAs that are mentioned in the manuscript. The same mini web site is available at http://lapti.ucc.ie/ogd/. [file 13059_2015_651_MOESM2_ESM.zip › ogd/profiles/NM_138823.png]

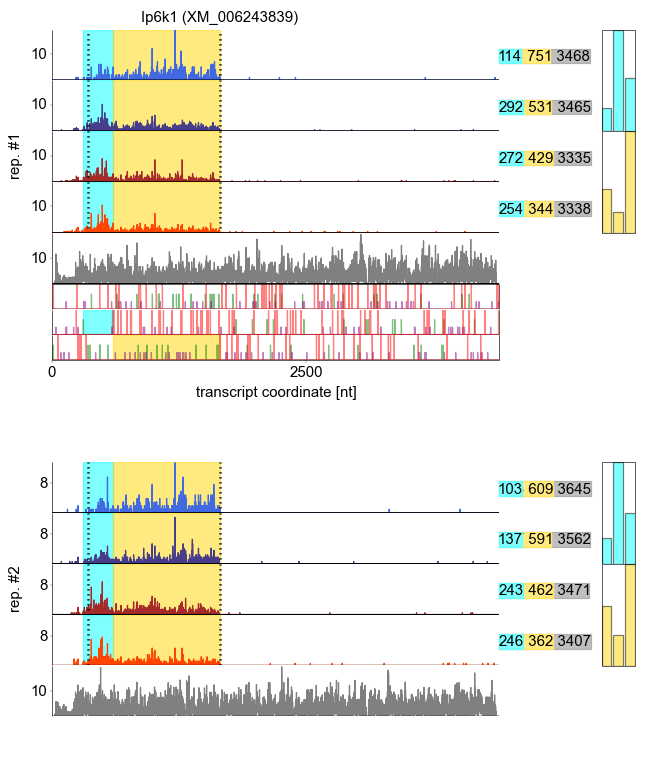

Supplement: Additional file 2: — This file contains a mini web site with additional ribosome profiles of individual mRNAs that are mentioned in the manuscript. The same mini web site is available at http://lapti.ucc.ie/ogd/. [file 13059_2015_651_MOESM2_ESM.zip › ogd/profiles/XM_006243839.png]

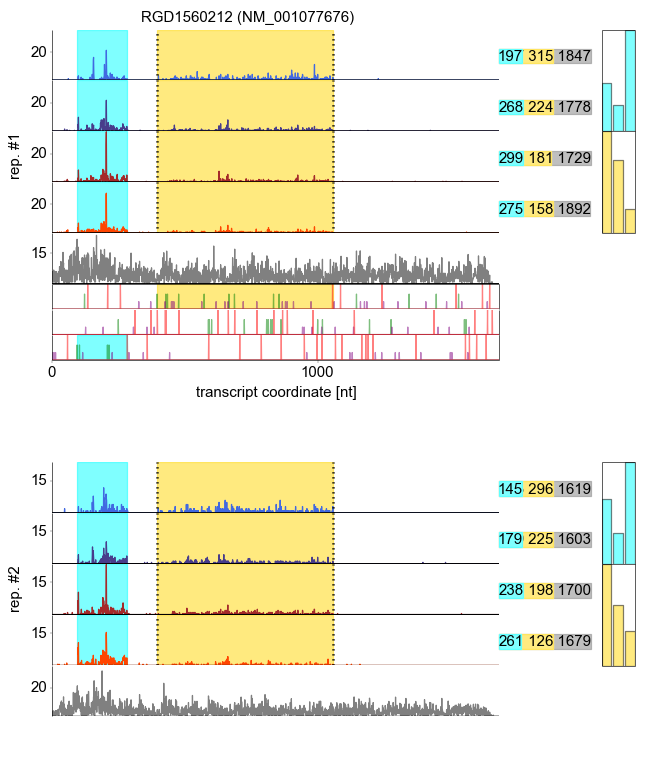

Supplement: Additional file 2: — This file contains a mini web site with additional ribosome profiles of individual mRNAs that are mentioned in the manuscript. The same mini web site is available at http://lapti.ucc.ie/ogd/. [file 13059_2015_651_MOESM2_ESM.zip › ogd/profiles/NM_001077676.png]

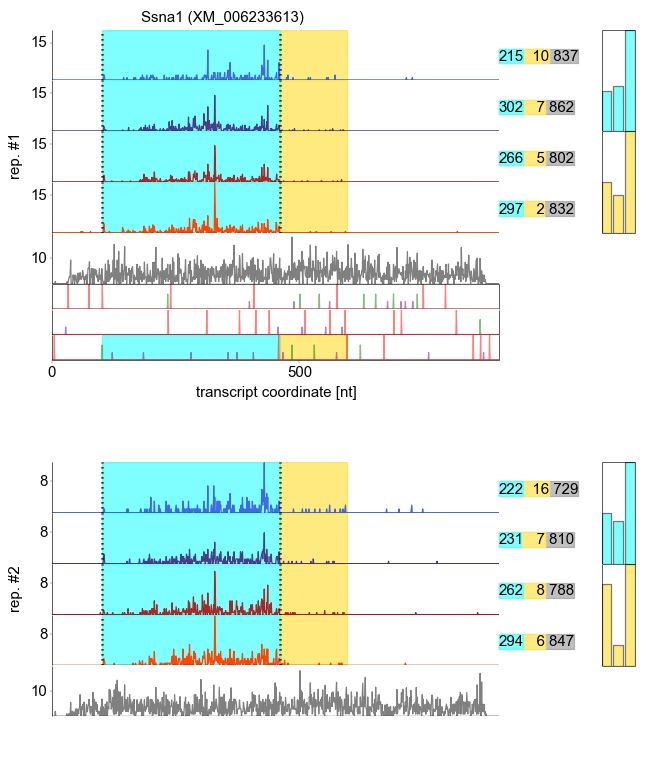

Supplement: Additional file 2: — This file contains a mini web site with additional ribosome profiles of individual mRNAs that are mentioned in the manuscript. The same mini web site is available at http://lapti.ucc.ie/ogd/. [file 13059_2015_651_MOESM2_ESM.zip › ogd/profiles/XM_006233613.png]

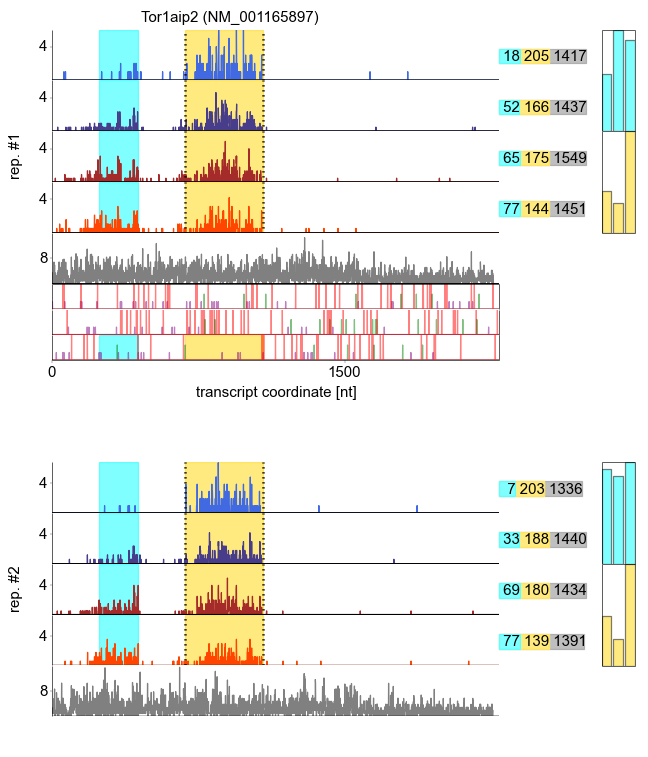

Supplement: Additional file 2: — This file contains a mini web site with additional ribosome profiles of individual mRNAs that are mentioned in the manuscript. The same mini web site is available at http://lapti.ucc.ie/ogd/. [file 13059_2015_651_MOESM2_ESM.zip › ogd/profiles/NM_001165897.png]

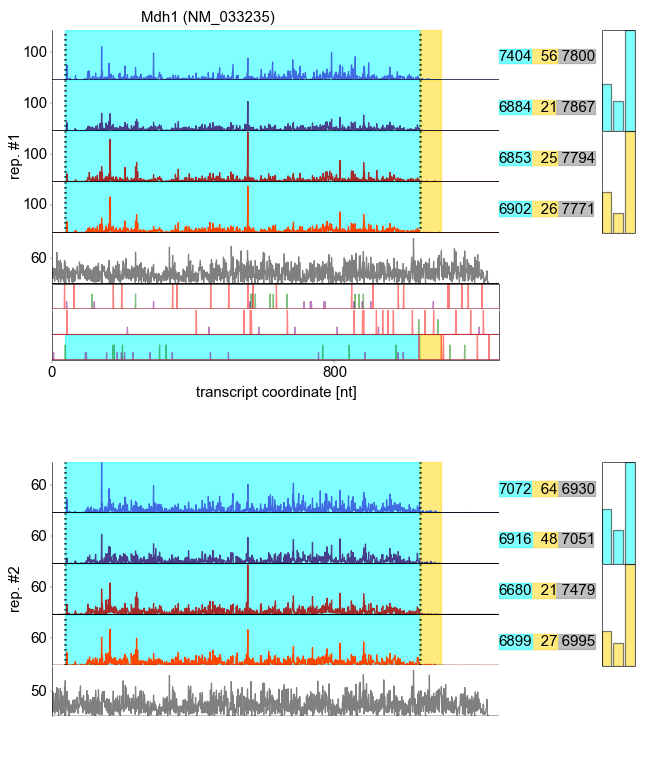

Supplement: Additional file 2: — This file contains a mini web site with additional ribosome profiles of individual mRNAs that are mentioned in the manuscript. The same mini web site is available at http://lapti.ucc.ie/ogd/. [file 13059_2015_651_MOESM2_ESM.zip › ogd/profiles/NM_033235.png]

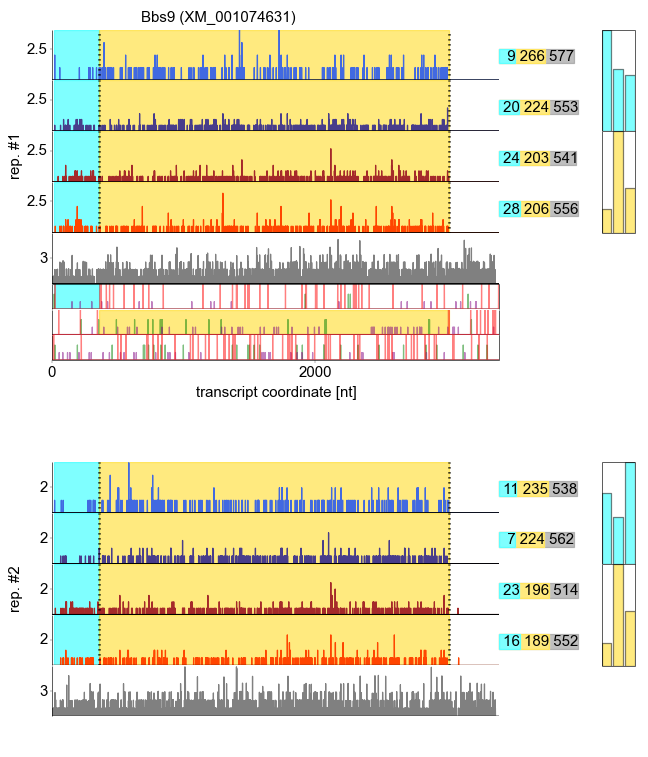

Supplement: Additional file 2: — This file contains a mini web site with additional ribosome profiles of individual mRNAs that are mentioned in the manuscript. The same mini web site is available at http://lapti.ucc.ie/ogd/. [file 13059_2015_651_MOESM2_ESM.zip › ogd/profiles/XM_001074631.png]

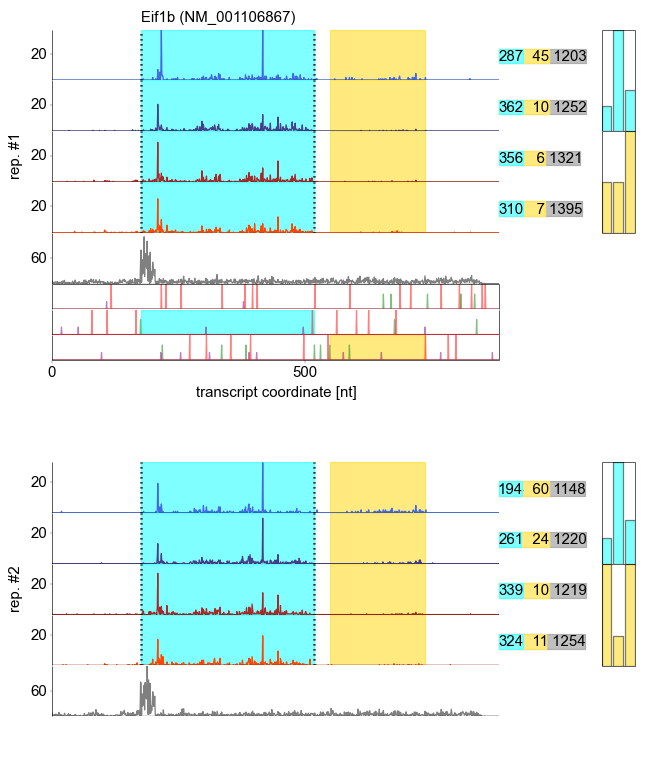

Supplement: Additional file 2: — This file contains a mini web site with additional ribosome profiles of individual mRNAs that are mentioned in the manuscript. The same mini web site is available at http://lapti.ucc.ie/ogd/. [file 13059_2015_651_MOESM2_ESM.zip › ogd/profiles/NM_001106867.png]

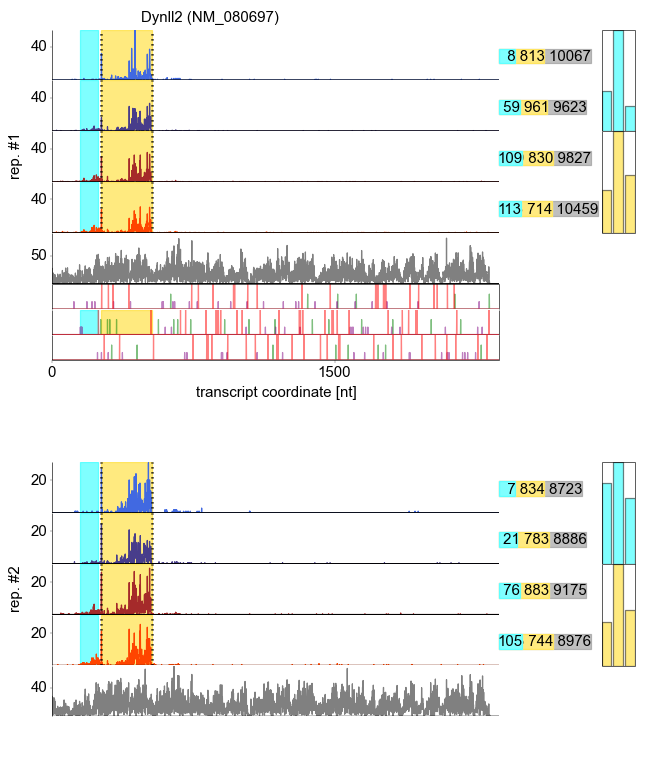

Supplement: Additional file 2: — This file contains a mini web site with additional ribosome profiles of individual mRNAs that are mentioned in the manuscript. The same mini web site is available at http://lapti.ucc.ie/ogd/. [file 13059_2015_651_MOESM2_ESM.zip › ogd/profiles/NM_080697.png]

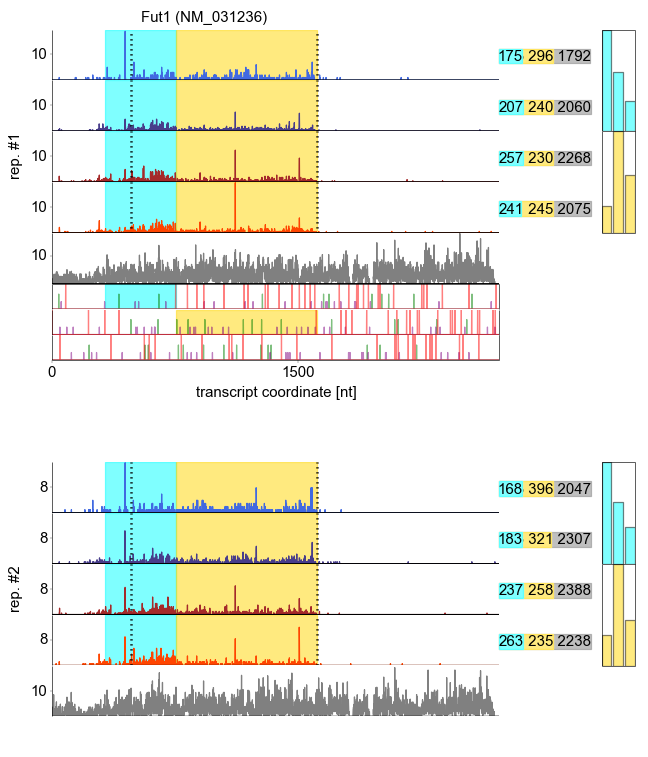

Supplement: Additional file 2: — This file contains a mini web site with additional ribosome profiles of individual mRNAs that are mentioned in the manuscript. The same mini web site is available at http://lapti.ucc.ie/ogd/. [file 13059_2015_651_MOESM2_ESM.zip › ogd/profiles/NM_031236.png]

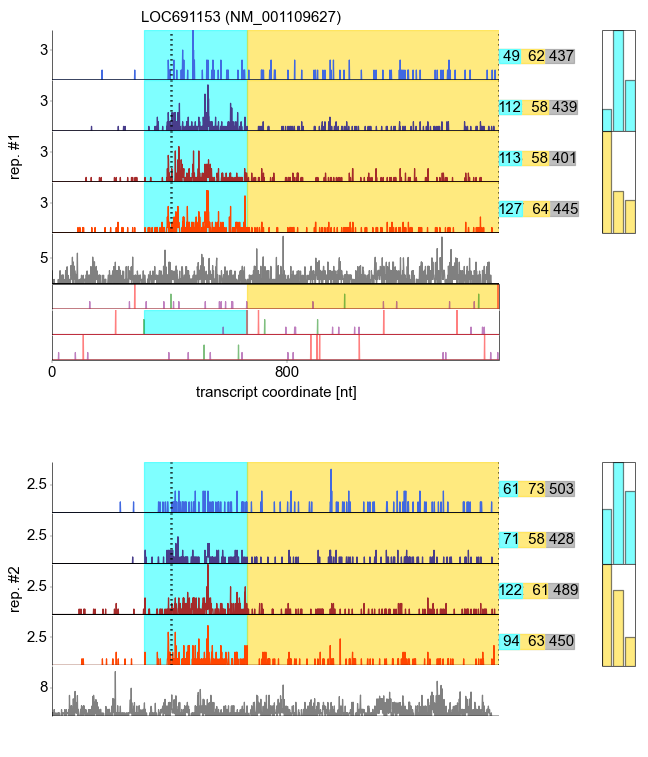

Supplement: Additional file 2: — This file contains a mini web site with additional ribosome profiles of individual mRNAs that are mentioned in the manuscript. The same mini web site is available at http://lapti.ucc.ie/ogd/. [file 13059_2015_651_MOESM2_ESM.zip › ogd/profiles/NM_001109627.png]

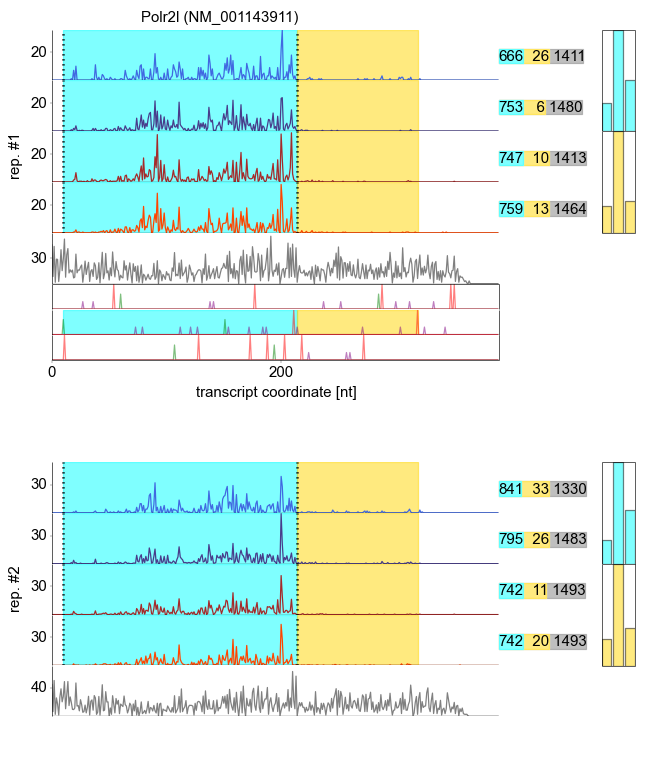

Supplement: Additional file 2: — This file contains a mini web site with additional ribosome profiles of individual mRNAs that are mentioned in the manuscript. The same mini web site is available at http://lapti.ucc.ie/ogd/. [file 13059_2015_651_MOESM2_ESM.zip › ogd/profiles/NM_001143911.png]

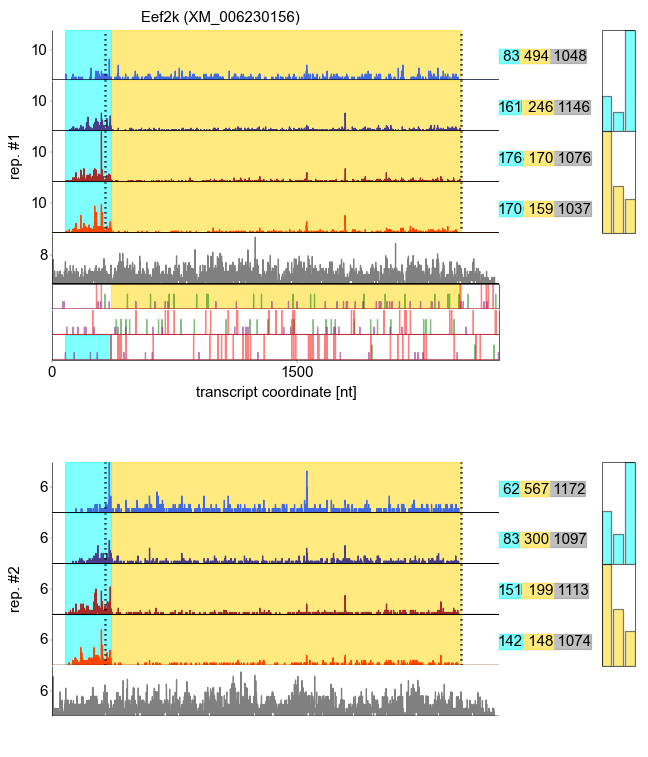

Supplement: Additional file 2: — This file contains a mini web site with additional ribosome profiles of individual mRNAs that are mentioned in the manuscript. The same mini web site is available at http://lapti.ucc.ie/ogd/. [file 13059_2015_651_MOESM2_ESM.zip › ogd/profiles/XM_006230156.png]

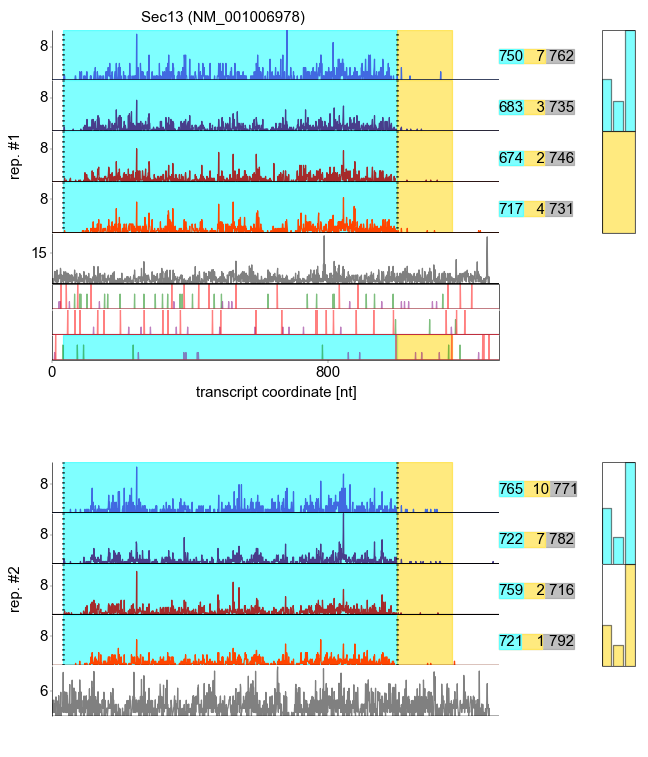

Supplement: Additional file 2: — This file contains a mini web site with additional ribosome profiles of individual mRNAs that are mentioned in the manuscript. The same mini web site is available at http://lapti.ucc.ie/ogd/. [file 13059_2015_651_MOESM2_ESM.zip › ogd/profiles/NM_001006978.png]

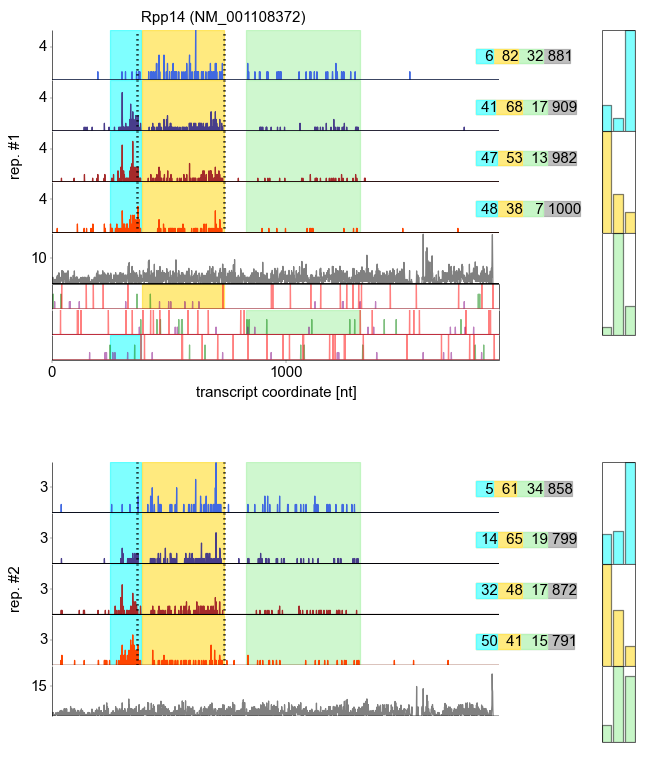

Supplement: Additional file 2: — This file contains a mini web site with additional ribosome profiles of individual mRNAs that are mentioned in the manuscript. The same mini web site is available at http://lapti.ucc.ie/ogd/. [file 13059_2015_651_MOESM2_ESM.zip › ogd/profiles/NM_001108372.png]

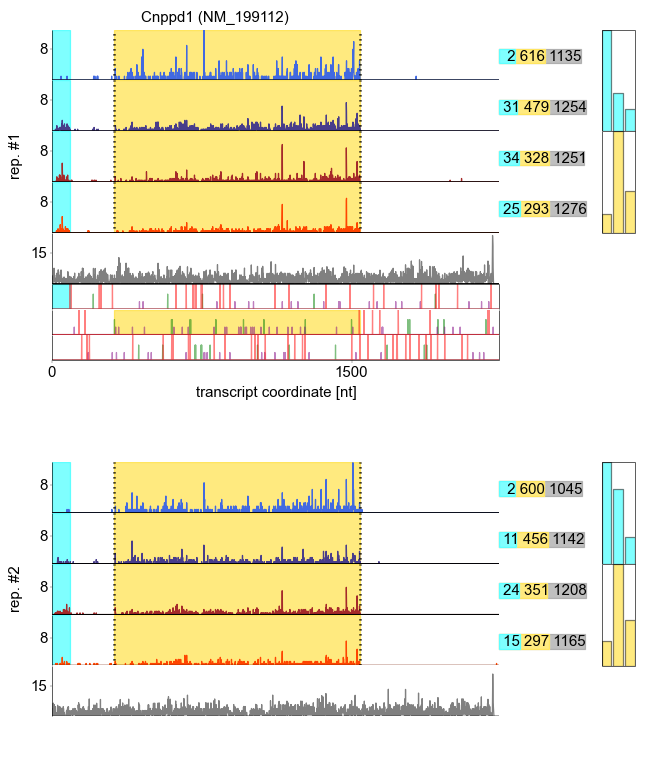

Supplement: Additional file 2: — This file contains a mini web site with additional ribosome profiles of individual mRNAs that are mentioned in the manuscript. The same mini web site is available at http://lapti.ucc.ie/ogd/. [file 13059_2015_651_MOESM2_ESM.zip › ogd/profiles/NM_199112.png]

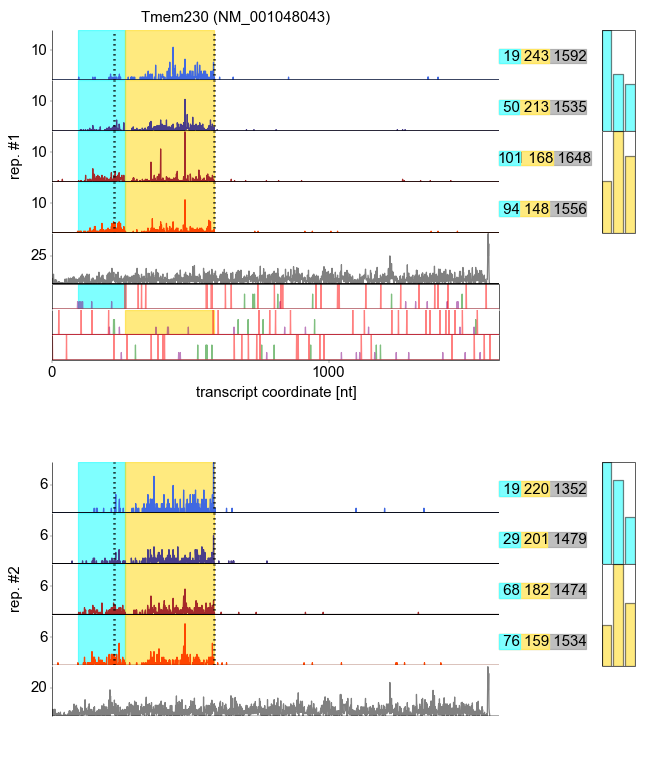

Supplement: Additional file 2: — This file contains a mini web site with additional ribosome profiles of individual mRNAs that are mentioned in the manuscript. The same mini web site is available at http://lapti.ucc.ie/ogd/. [file 13059_2015_651_MOESM2_ESM.zip › ogd/profiles/NM_001048043.png]

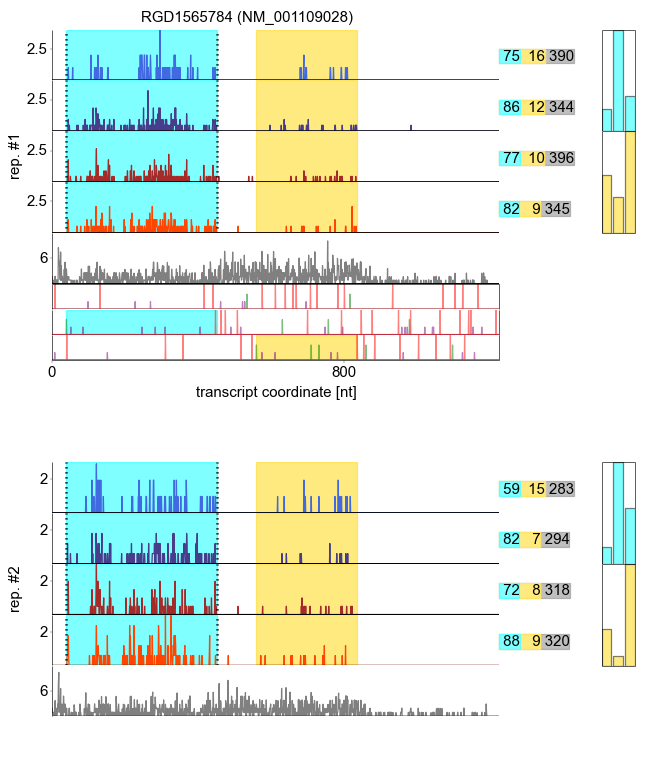

Supplement: Additional file 2: — This file contains a mini web site with additional ribosome profiles of individual mRNAs that are mentioned in the manuscript. The same mini web site is available at http://lapti.ucc.ie/ogd/. [file 13059_2015_651_MOESM2_ESM.zip › ogd/profiles/NM_001109028.png]

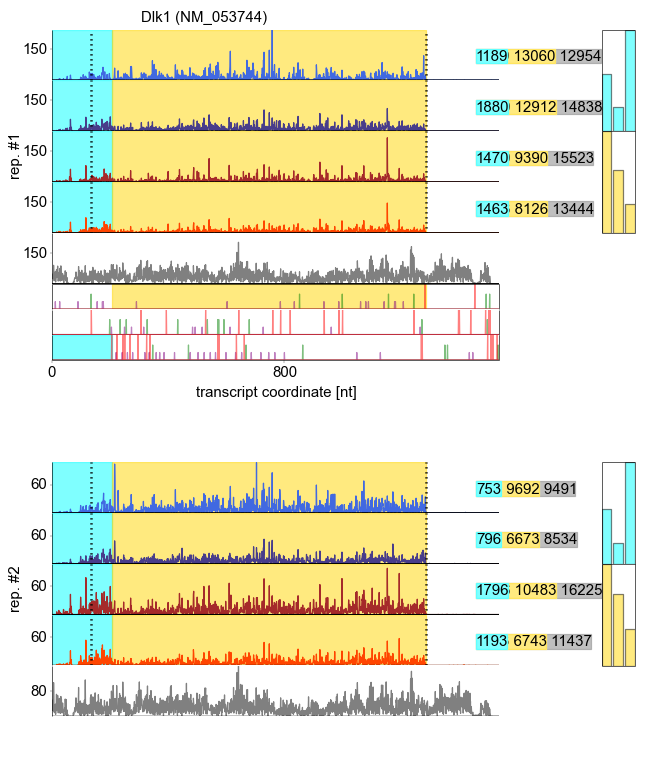

Supplement: Additional file 2: — This file contains a mini web site with additional ribosome profiles of individual mRNAs that are mentioned in the manuscript. The same mini web site is available at http://lapti.ucc.ie/ogd/. [file 13059_2015_651_MOESM2_ESM.zip › ogd/profiles/NM_053744.png]

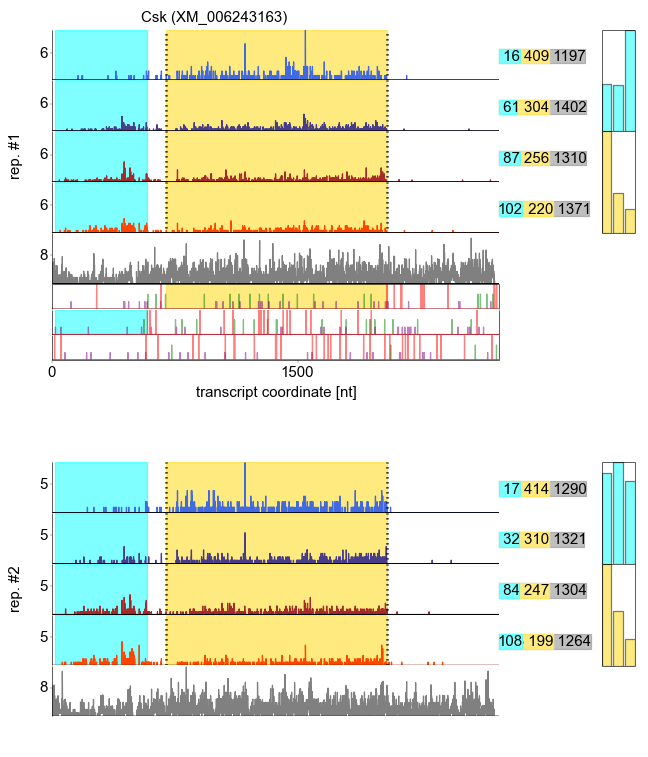

Supplement: Additional file 2: — This file contains a mini web site with additional ribosome profiles of individual mRNAs that are mentioned in the manuscript. The same mini web site is available at http://lapti.ucc.ie/ogd/. [file 13059_2015_651_MOESM2_ESM.zip › ogd/profiles/XM_006243163.png]

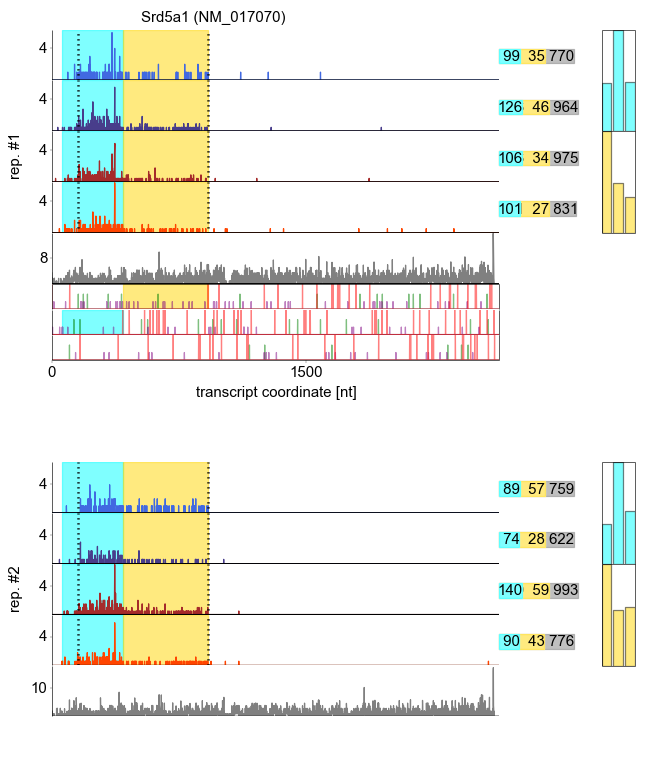

Supplement: Additional file 2: — This file contains a mini web site with additional ribosome profiles of individual mRNAs that are mentioned in the manuscript. The same mini web site is available at http://lapti.ucc.ie/ogd/. [file 13059_2015_651_MOESM2_ESM.zip › ogd/profiles/NM_017070.png]

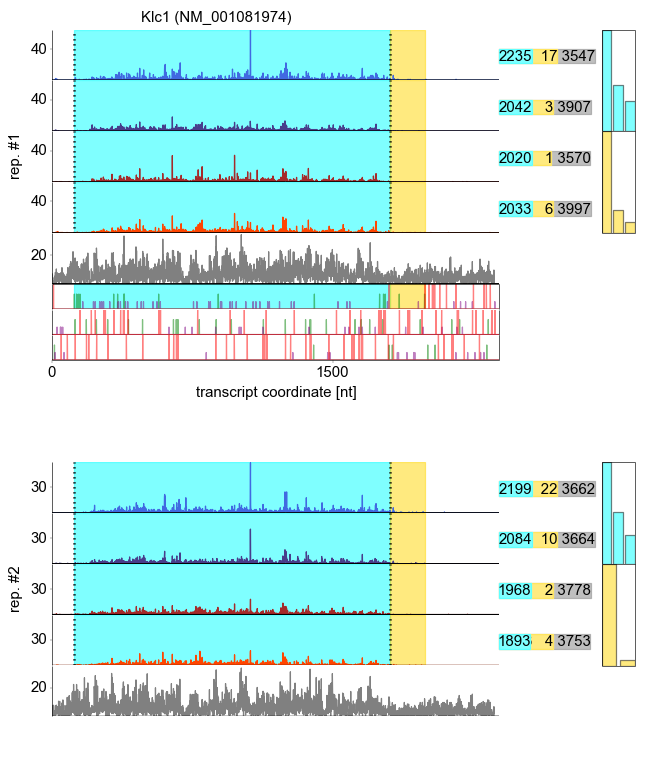

Supplement: Additional file 2: — This file contains a mini web site with additional ribosome profiles of individual mRNAs that are mentioned in the manuscript. The same mini web site is available at http://lapti.ucc.ie/ogd/. [file 13059_2015_651_MOESM2_ESM.zip › ogd/profiles/NM_001081974.png]

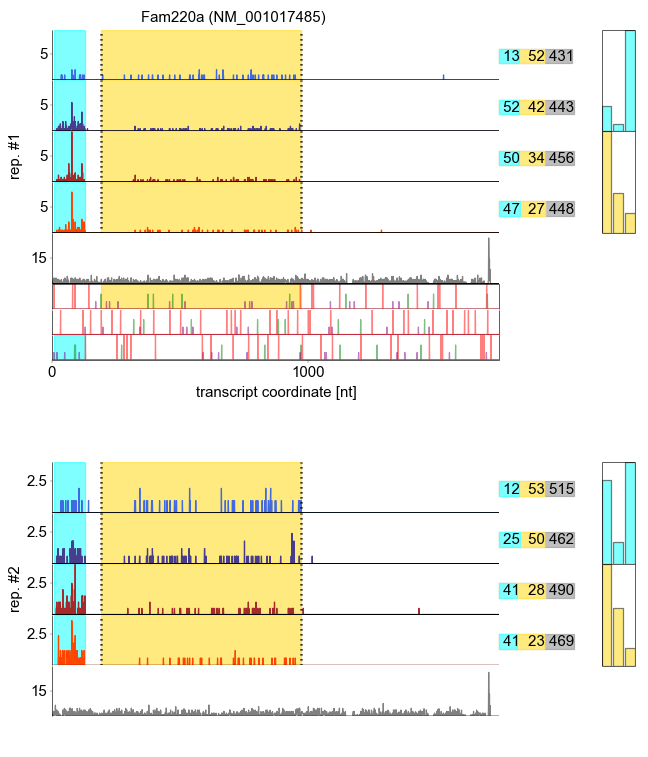

Supplement: Additional file 2: — This file contains a mini web site with additional ribosome profiles of individual mRNAs that are mentioned in the manuscript. The same mini web site is available at http://lapti.ucc.ie/ogd/. [file 13059_2015_651_MOESM2_ESM.zip › ogd/profiles/NM_001017485.png]

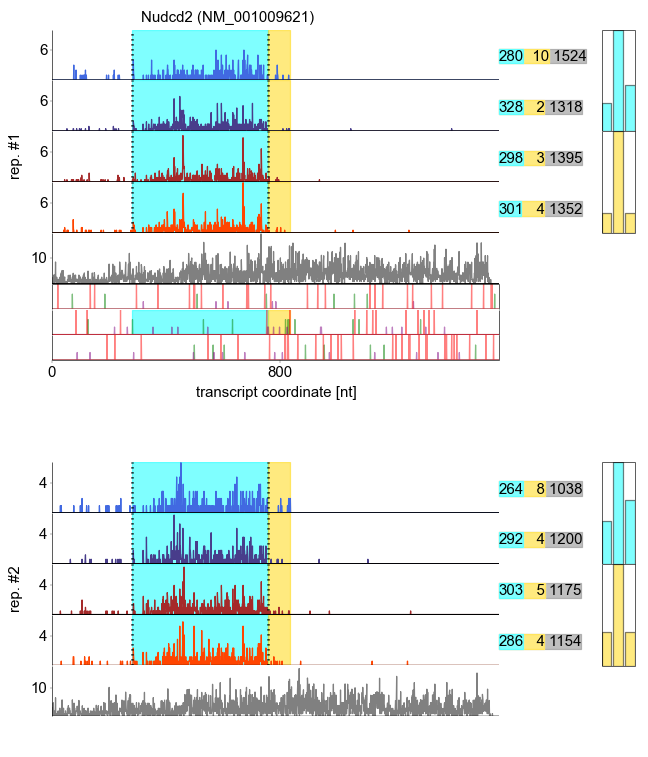

Supplement: Additional file 2: — This file contains a mini web site with additional ribosome profiles of individual mRNAs that are mentioned in the manuscript. The same mini web site is available at http://lapti.ucc.ie/ogd/. [file 13059_2015_651_MOESM2_ESM.zip › ogd/profiles/NM_001009621.png]

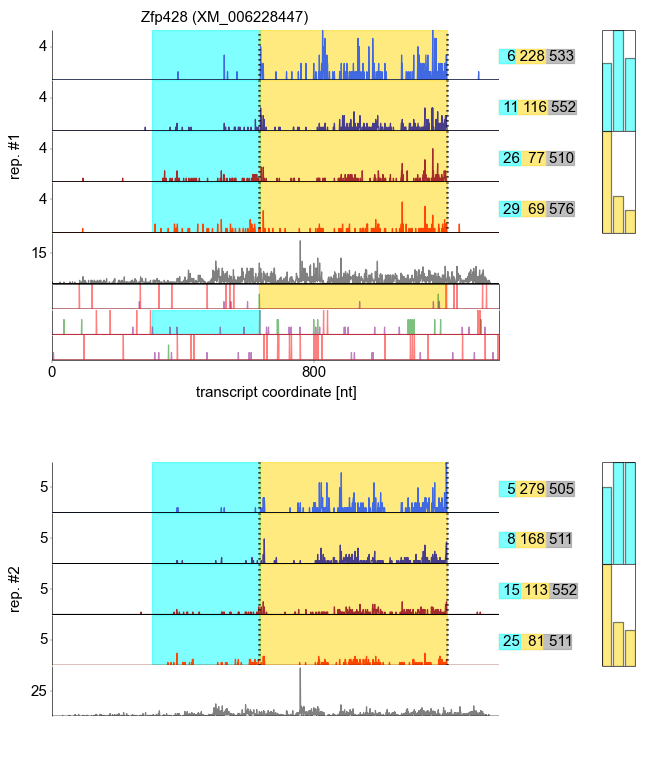

Supplement: Additional file 2: — This file contains a mini web site with additional ribosome profiles of individual mRNAs that are mentioned in the manuscript. The same mini web site is available at http://lapti.ucc.ie/ogd/. [file 13059_2015_651_MOESM2_ESM.zip › ogd/profiles/XM_006228447.png]

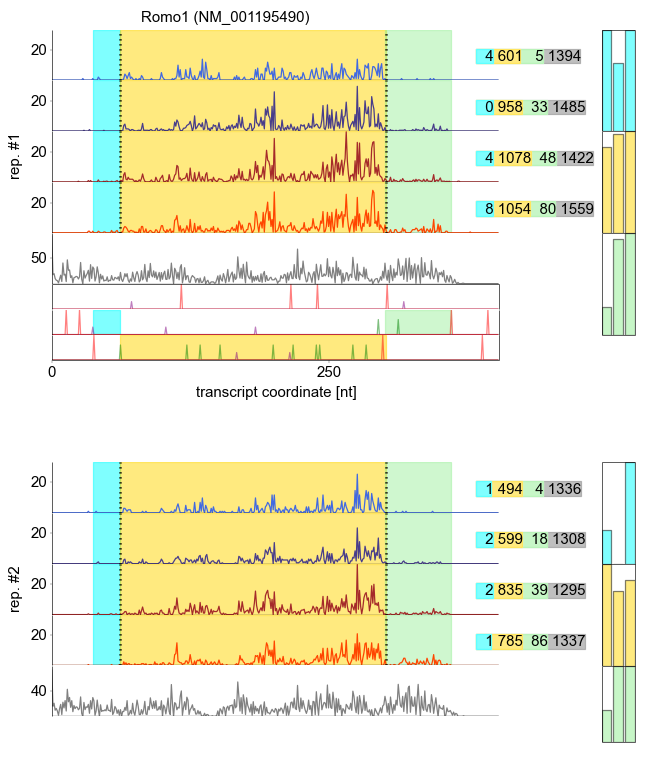

Supplement: Additional file 2: — This file contains a mini web site with additional ribosome profiles of individual mRNAs that are mentioned in the manuscript. The same mini web site is available at http://lapti.ucc.ie/ogd/. [file 13059_2015_651_MOESM2_ESM.zip › ogd/profiles/NM_001195490.png]

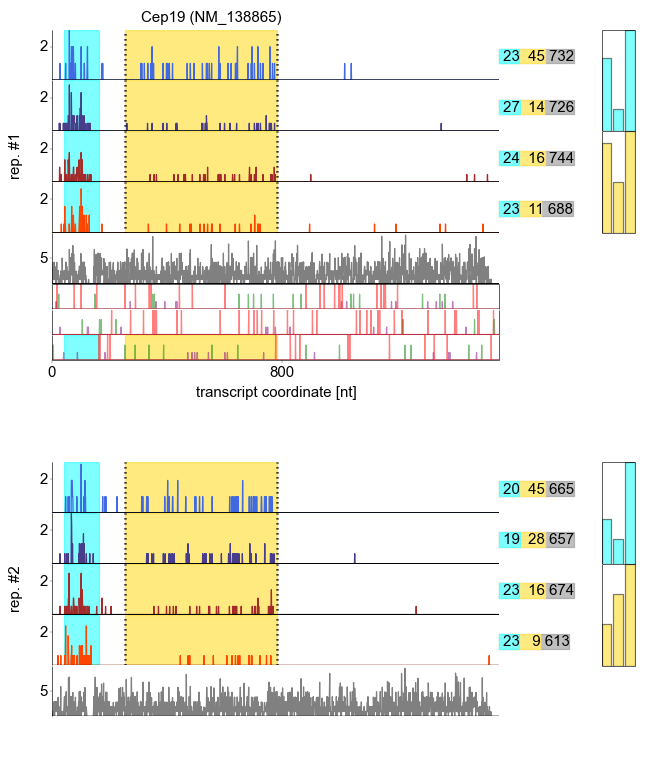

Supplement: Additional file 2: — This file contains a mini web site with additional ribosome profiles of individual mRNAs that are mentioned in the manuscript. The same mini web site is available at http://lapti.ucc.ie/ogd/. [file 13059_2015_651_MOESM2_ESM.zip › ogd/profiles/NM_138865.png]

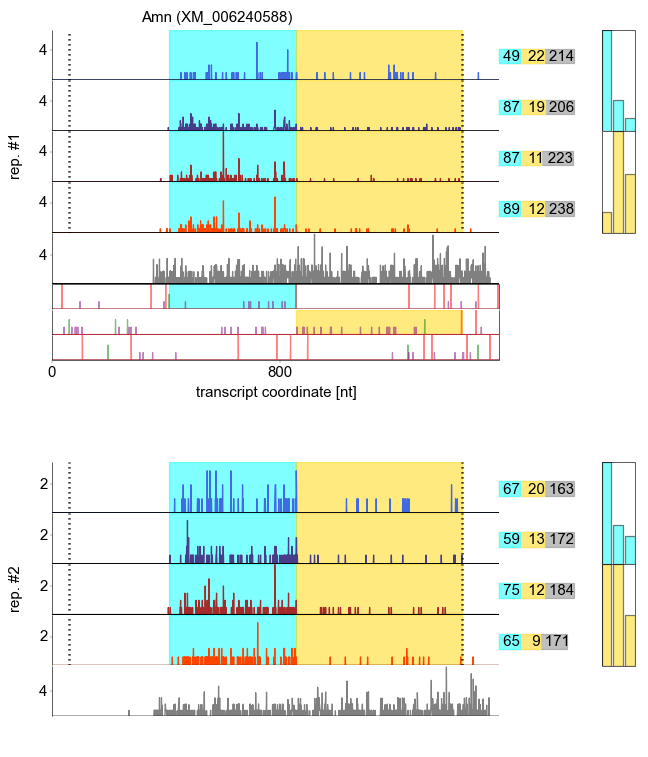

Supplement: Additional file 2: — This file contains a mini web site with additional ribosome profiles of individual mRNAs that are mentioned in the manuscript. The same mini web site is available at http://lapti.ucc.ie/ogd/. [file 13059_2015_651_MOESM2_ESM.zip › ogd/profiles/XM_006240588.png]

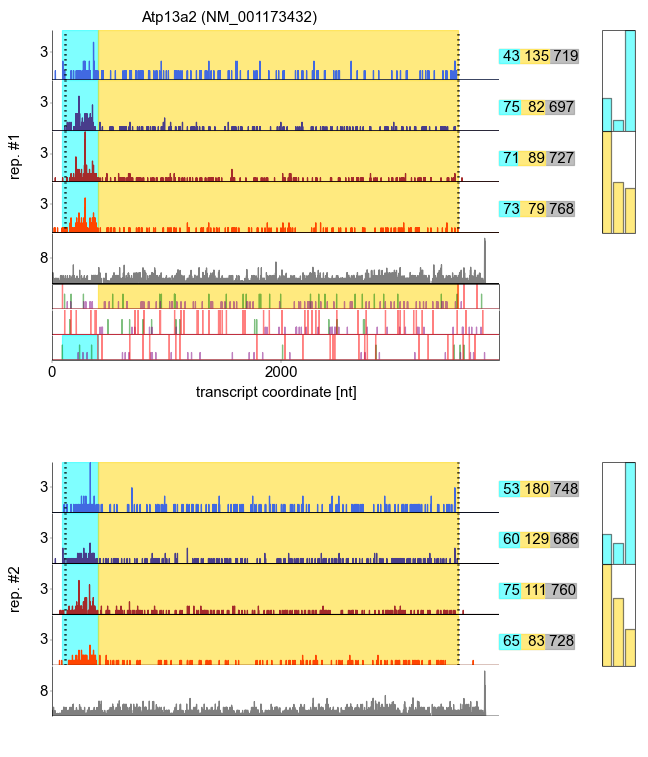

Supplement: Additional file 2: — This file contains a mini web site with additional ribosome profiles of individual mRNAs that are mentioned in the manuscript. The same mini web site is available at http://lapti.ucc.ie/ogd/. [file 13059_2015_651_MOESM2_ESM.zip › ogd/profiles/NM_001173432.png]

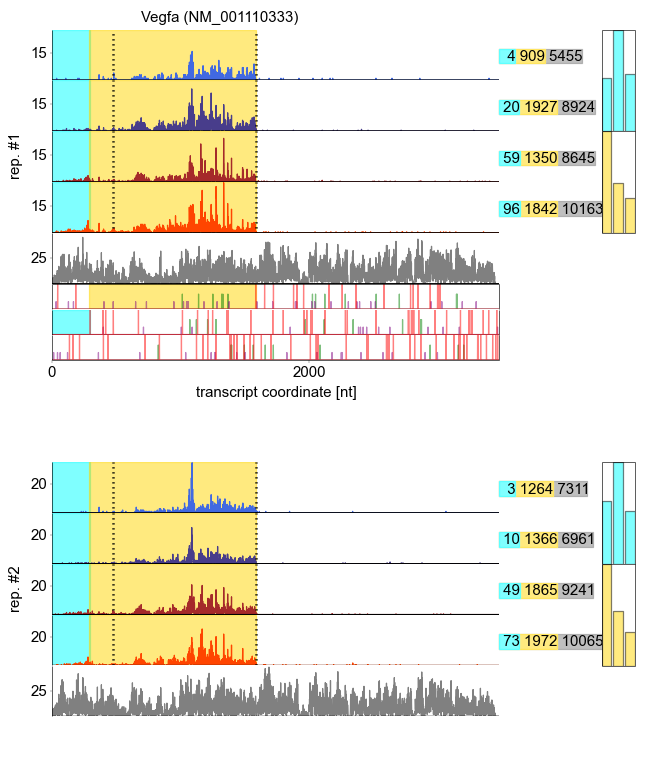

Supplement: Additional file 2: — This file contains a mini web site with additional ribosome profiles of individual mRNAs that are mentioned in the manuscript. The same mini web site is available at http://lapti.ucc.ie/ogd/. [file 13059_2015_651_MOESM2_ESM.zip › ogd/profiles/NM_001110333.png]

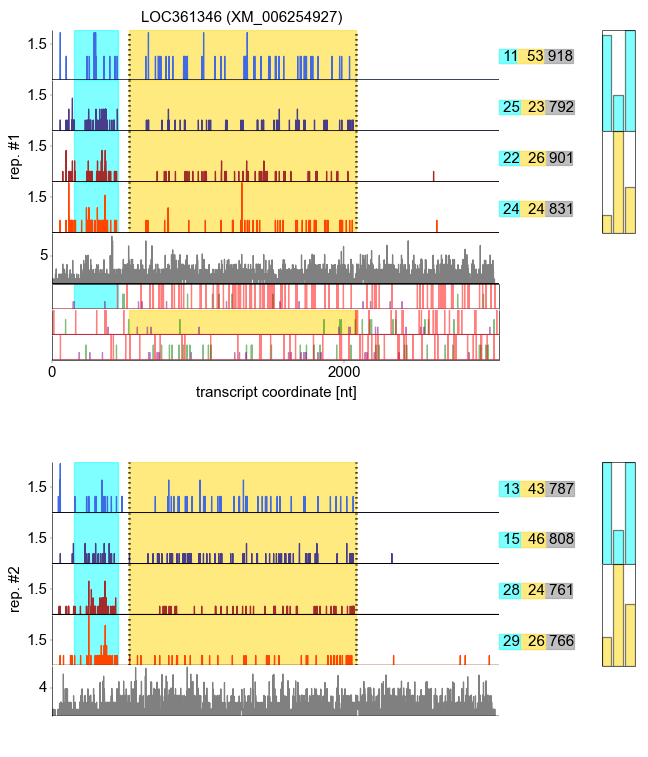

Supplement: Additional file 2: — This file contains a mini web site with additional ribosome profiles of individual mRNAs that are mentioned in the manuscript. The same mini web site is available at http://lapti.ucc.ie/ogd/. [file 13059_2015_651_MOESM2_ESM.zip › ogd/profiles/XM_006254927.png]

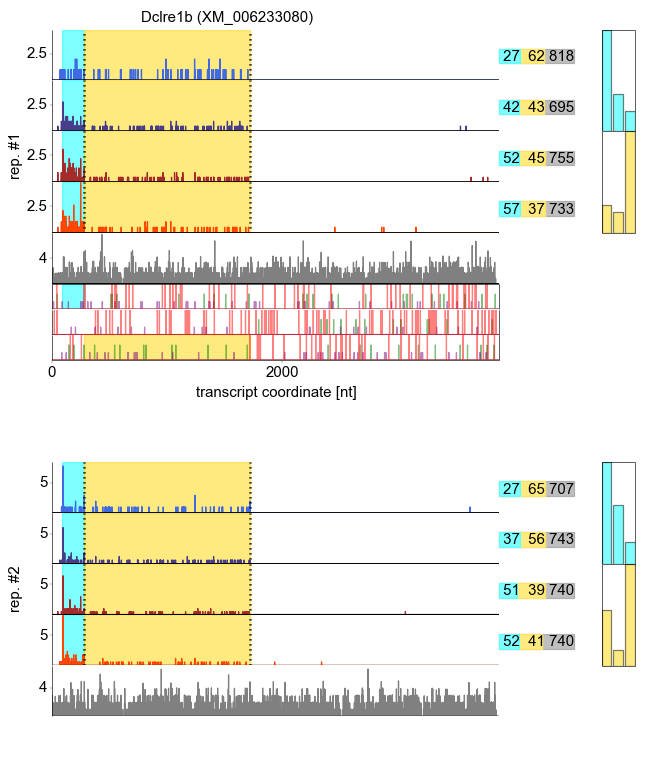

Supplement: Additional file 2: — This file contains a mini web site with additional ribosome profiles of individual mRNAs that are mentioned in the manuscript. The same mini web site is available at http://lapti.ucc.ie/ogd/. [file 13059_2015_651_MOESM2_ESM.zip › ogd/profiles/XM_006233080.png]

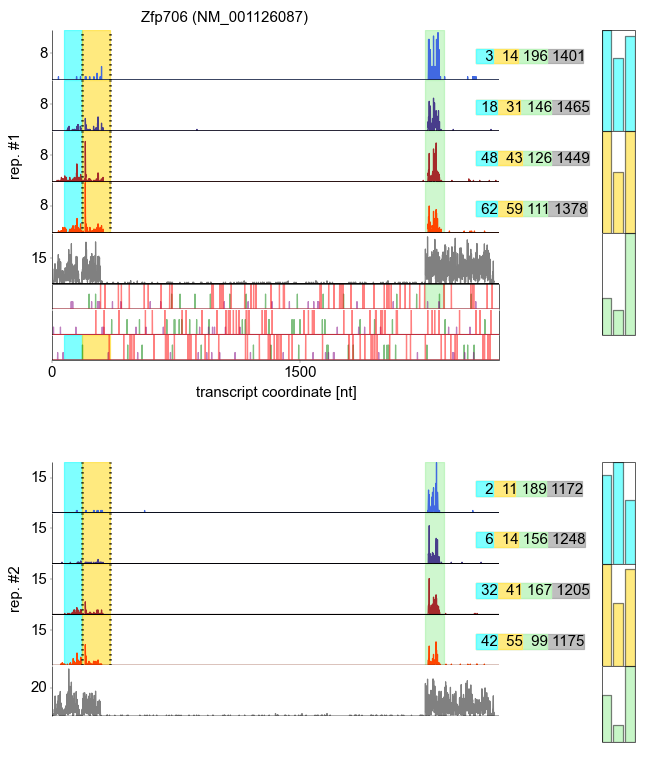

Supplement: Additional file 2: — This file contains a mini web site with additional ribosome profiles of individual mRNAs that are mentioned in the manuscript. The same mini web site is available at http://lapti.ucc.ie/ogd/. [file 13059_2015_651_MOESM2_ESM.zip › ogd/profiles/NM_001126087.png]

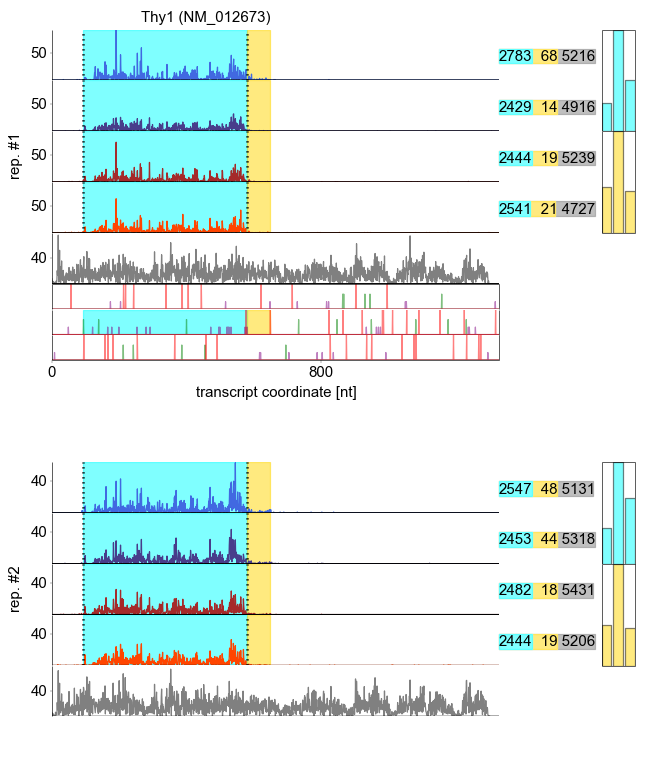

Supplement: Additional file 2: — This file contains a mini web site with additional ribosome profiles of individual mRNAs that are mentioned in the manuscript. The same mini web site is available at http://lapti.ucc.ie/ogd/. [file 13059_2015_651_MOESM2_ESM.zip › ogd/profiles/NM_012673.png]

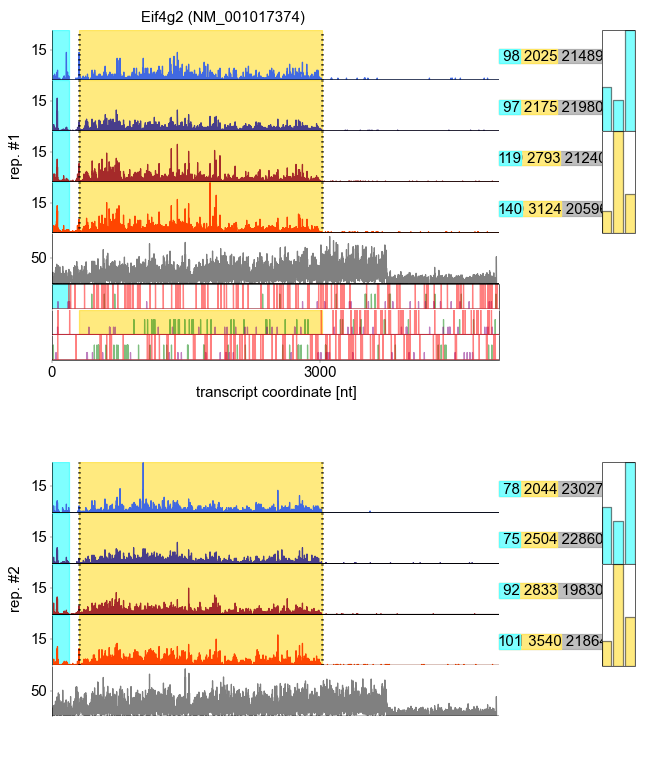

Supplement: Additional file 2: — This file contains a mini web site with additional ribosome profiles of individual mRNAs that are mentioned in the manuscript. The same mini web site is available at http://lapti.ucc.ie/ogd/. [file 13059_2015_651_MOESM2_ESM.zip › ogd/profiles/NM_001017374.png]

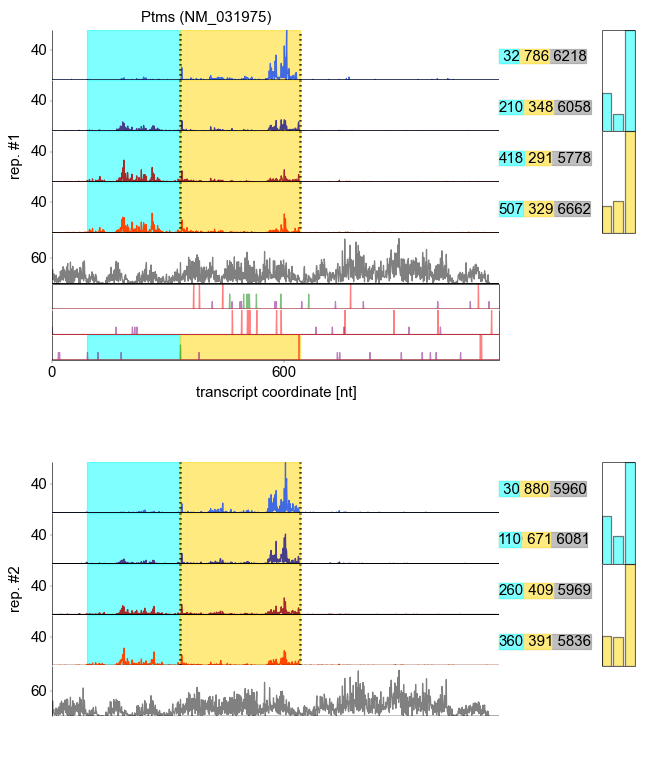

Supplement: Additional file 2: — This file contains a mini web site with additional ribosome profiles of individual mRNAs that are mentioned in the manuscript. The same mini web site is available at http://lapti.ucc.ie/ogd/. [file 13059_2015_651_MOESM2_ESM.zip › ogd/profiles/NM_031975.png]

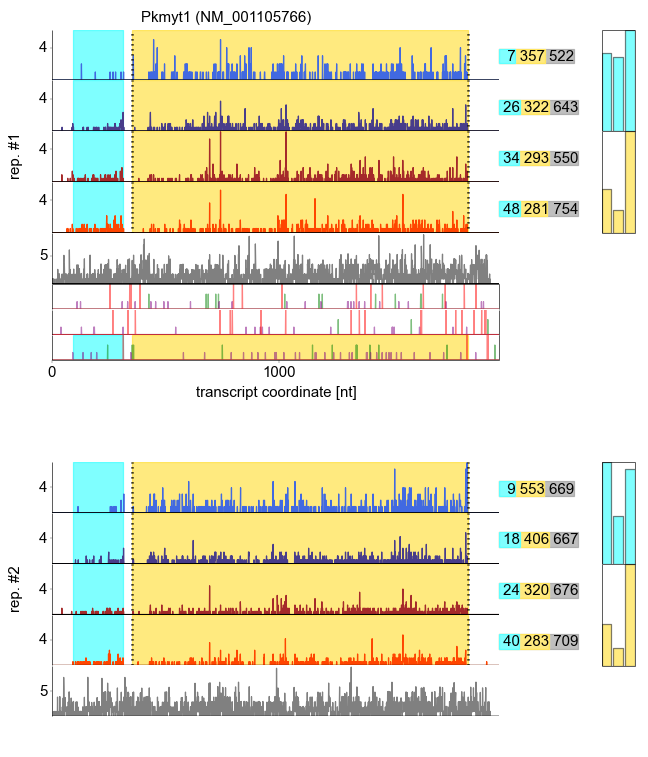

Supplement: Additional file 2: — This file contains a mini web site with additional ribosome profiles of individual mRNAs that are mentioned in the manuscript. The same mini web site is available at http://lapti.ucc.ie/ogd/. [file 13059_2015_651_MOESM2_ESM.zip › ogd/profiles/NM_001105766.png]

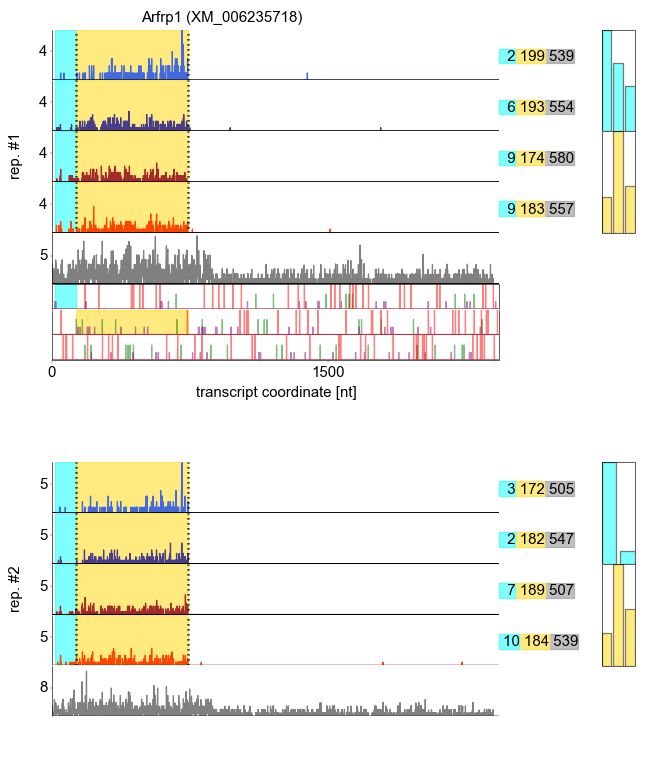

Supplement: Additional file 2: — This file contains a mini web site with additional ribosome profiles of individual mRNAs that are mentioned in the manuscript. The same mini web site is available at http://lapti.ucc.ie/ogd/. [file 13059_2015_651_MOESM2_ESM.zip › ogd/profiles/XM_006235718.png]

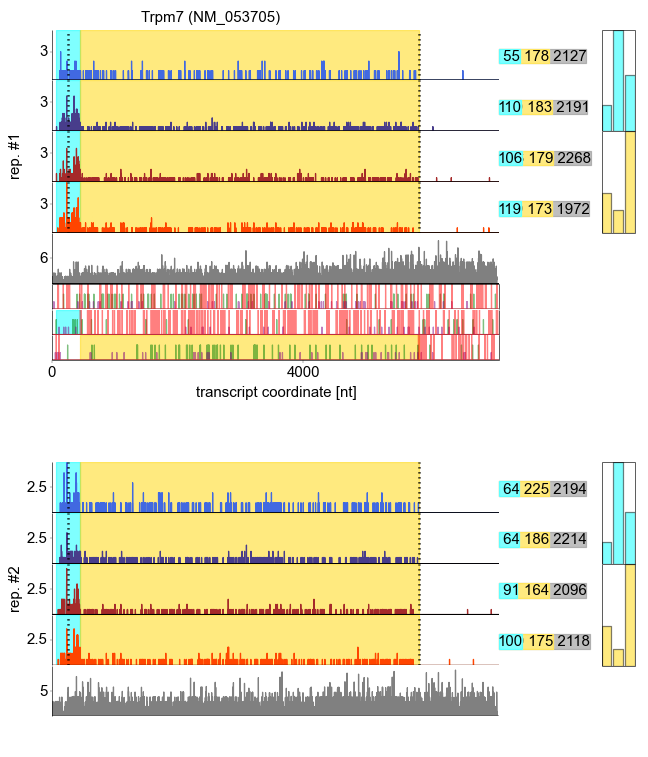

Supplement: Additional file 2: — This file contains a mini web site with additional ribosome profiles of individual mRNAs that are mentioned in the manuscript. The same mini web site is available at http://lapti.ucc.ie/ogd/. [file 13059_2015_651_MOESM2_ESM.zip › ogd/profiles/NM_053705.png]

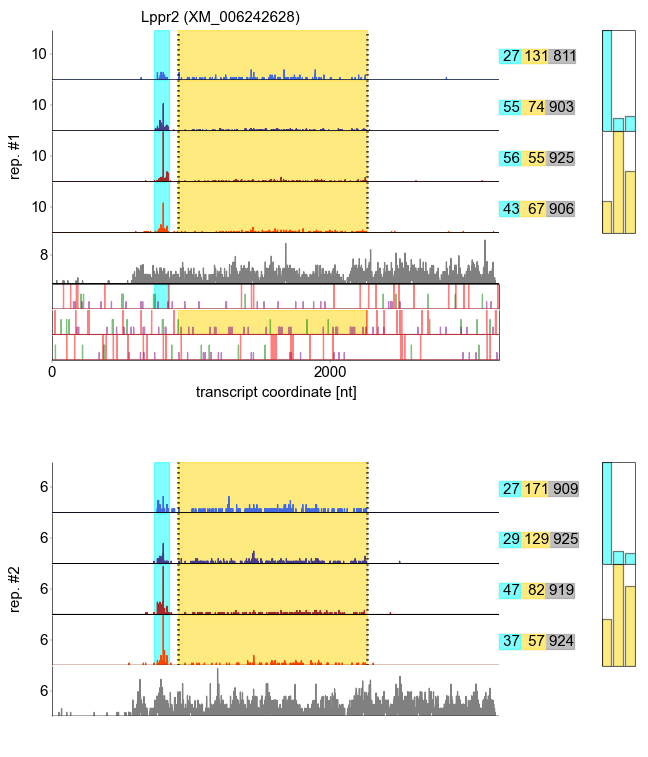

Supplement: Additional file 2: — This file contains a mini web site with additional ribosome profiles of individual mRNAs that are mentioned in the manuscript. The same mini web site is available at http://lapti.ucc.ie/ogd/. [file 13059_2015_651_MOESM2_ESM.zip › ogd/profiles/XM_006242628.png]

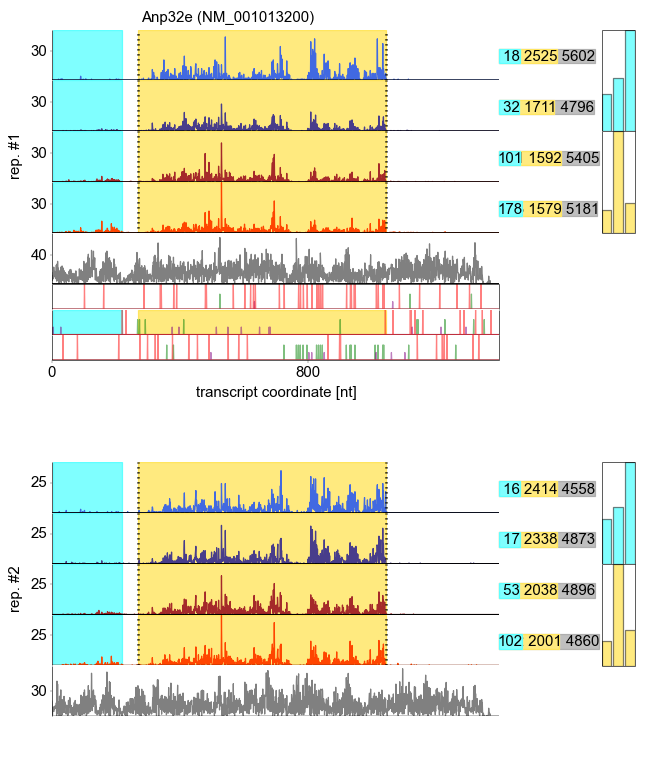

Supplement: Additional file 2: — This file contains a mini web site with additional ribosome profiles of individual mRNAs that are mentioned in the manuscript. The same mini web site is available at http://lapti.ucc.ie/ogd/. [file 13059_2015_651_MOESM2_ESM.zip › ogd/profiles/NM_001013200.png]

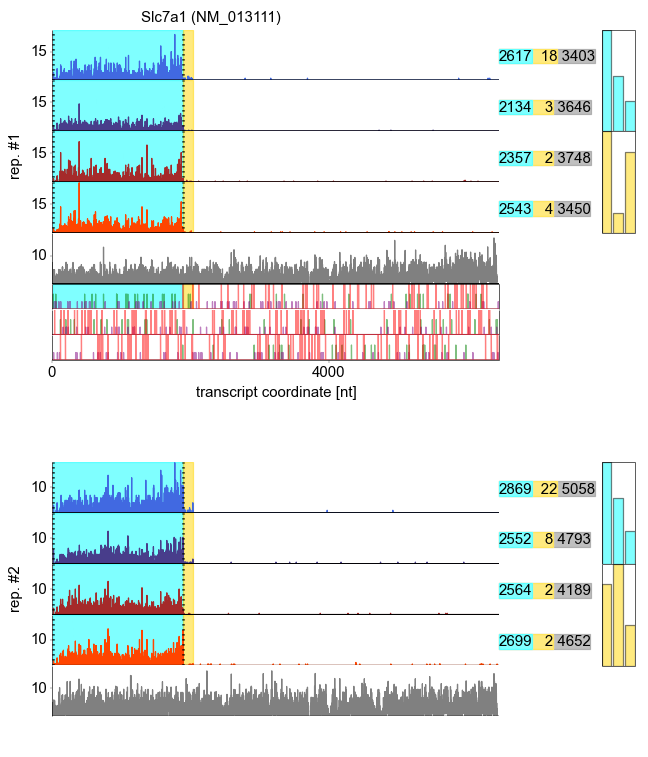

Supplement: Additional file 2: — This file contains a mini web site with additional ribosome profiles of individual mRNAs that are mentioned in the manuscript. The same mini web site is available at http://lapti.ucc.ie/ogd/. [file 13059_2015_651_MOESM2_ESM.zip › ogd/profiles/NM_013111.png]

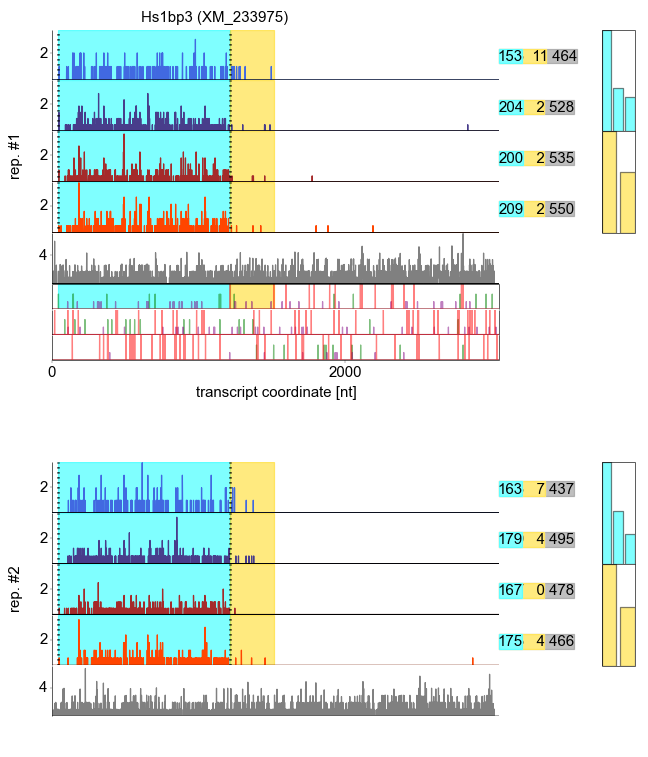

Supplement: Additional file 2: — This file contains a mini web site with additional ribosome profiles of individual mRNAs that are mentioned in the manuscript. The same mini web site is available at http://lapti.ucc.ie/ogd/. [file 13059_2015_651_MOESM2_ESM.zip › ogd/profiles/XM_233975.png]

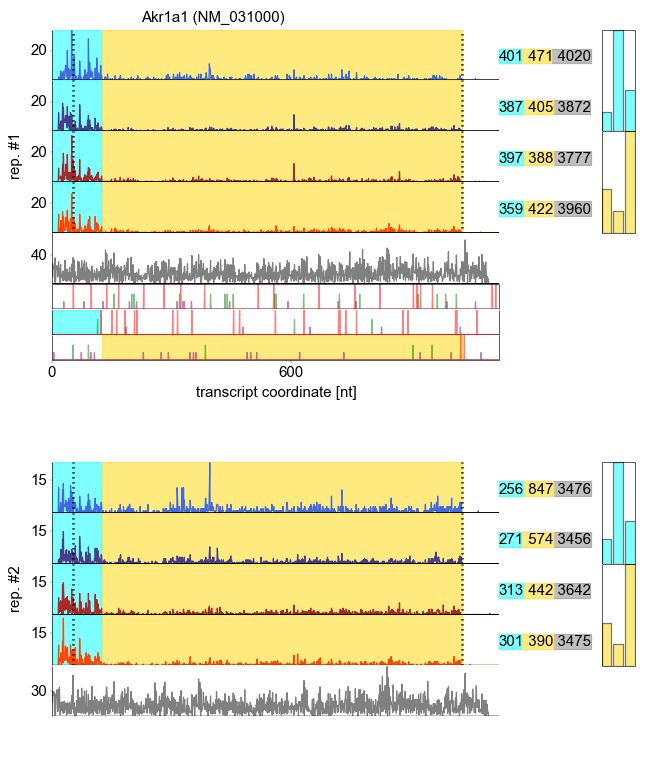

Supplement: Additional file 2: — This file contains a mini web site with additional ribosome profiles of individual mRNAs that are mentioned in the manuscript. The same mini web site is available at http://lapti.ucc.ie/ogd/. [file 13059_2015_651_MOESM2_ESM.zip › ogd/profiles/NM_031000.png]

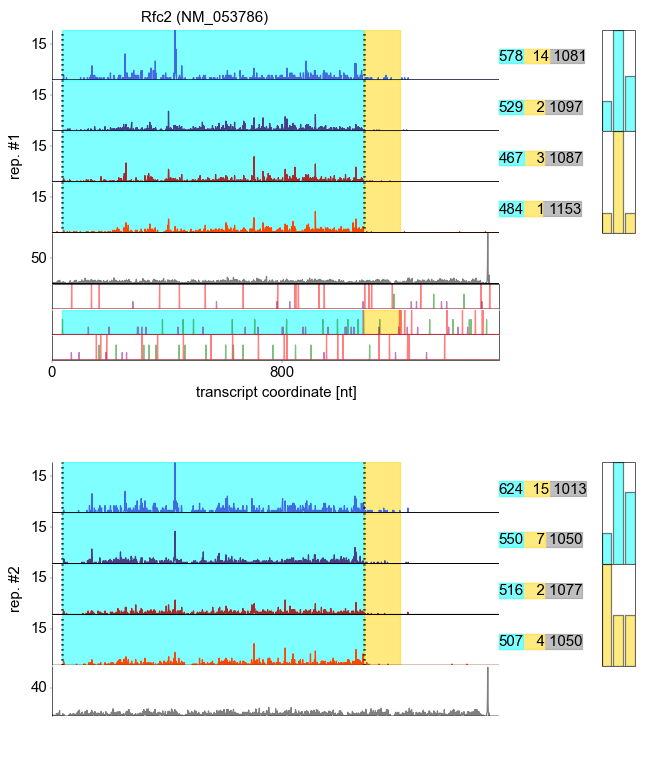

Supplement: Additional file 2: — This file contains a mini web site with additional ribosome profiles of individual mRNAs that are mentioned in the manuscript. The same mini web site is available at http://lapti.ucc.ie/ogd/. [file 13059_2015_651_MOESM2_ESM.zip › ogd/profiles/NM_053786.png]

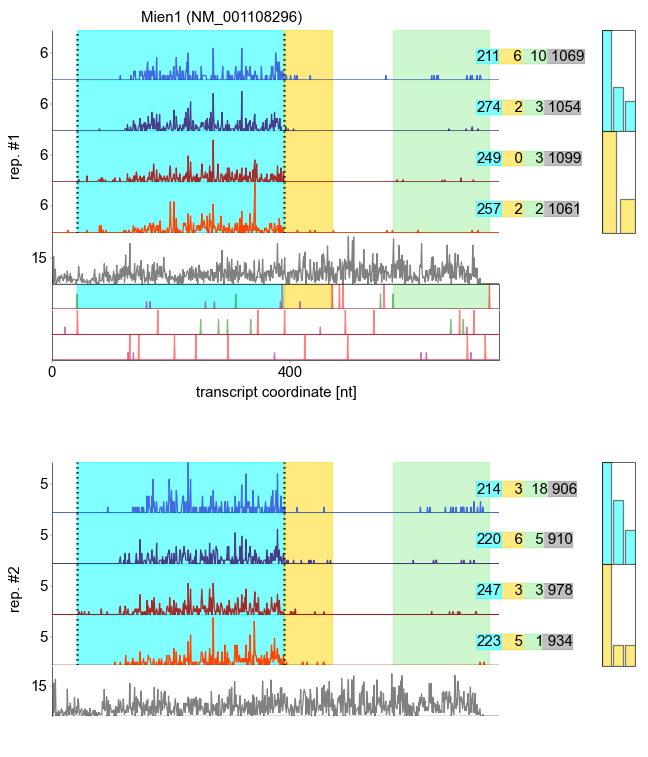

Supplement: Additional file 2: — This file contains a mini web site with additional ribosome profiles of individual mRNAs that are mentioned in the manuscript. The same mini web site is available at http://lapti.ucc.ie/ogd/. [file 13059_2015_651_MOESM2_ESM.zip › ogd/profiles/NM_001108296.png]

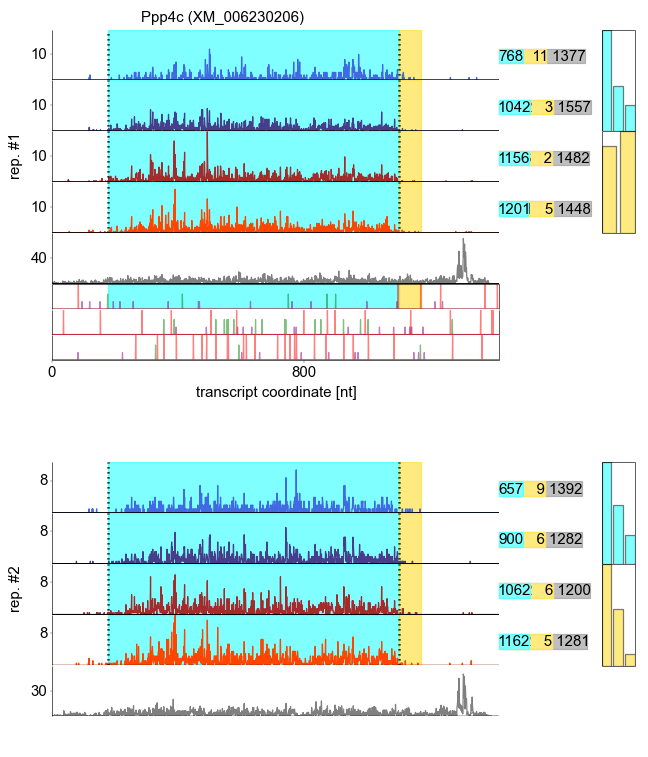

Supplement: Additional file 2: — This file contains a mini web site with additional ribosome profiles of individual mRNAs that are mentioned in the manuscript. The same mini web site is available at http://lapti.ucc.ie/ogd/. [file 13059_2015_651_MOESM2_ESM.zip › ogd/profiles/XM_006230206.png]

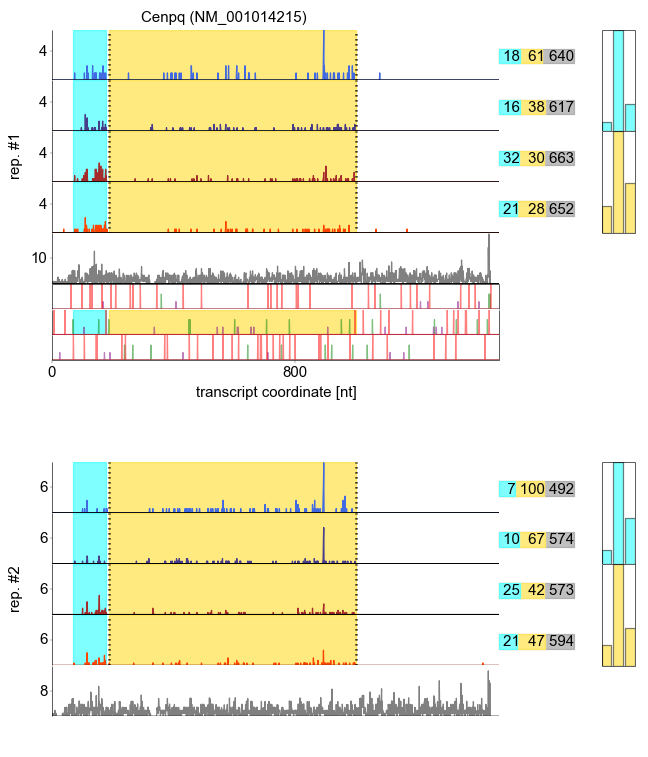

Supplement: Additional file 2: — This file contains a mini web site with additional ribosome profiles of individual mRNAs that are mentioned in the manuscript. The same mini web site is available at http://lapti.ucc.ie/ogd/. [file 13059_2015_651_MOESM2_ESM.zip › ogd/profiles/NM_001014215.png]

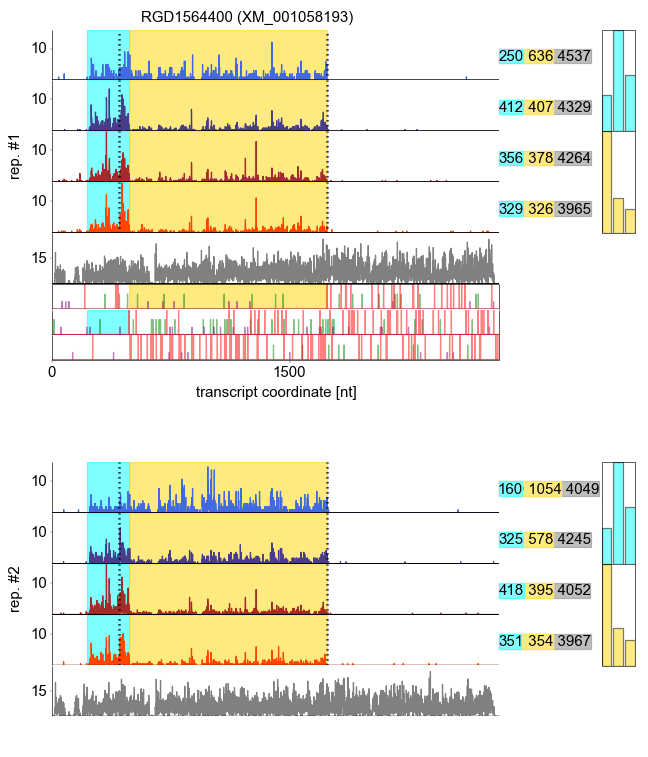

Supplement: Additional file 2: — This file contains a mini web site with additional ribosome profiles of individual mRNAs that are mentioned in the manuscript. The same mini web site is available at http://lapti.ucc.ie/ogd/. [file 13059_2015_651_MOESM2_ESM.zip › ogd/profiles/XM_001058193.png]

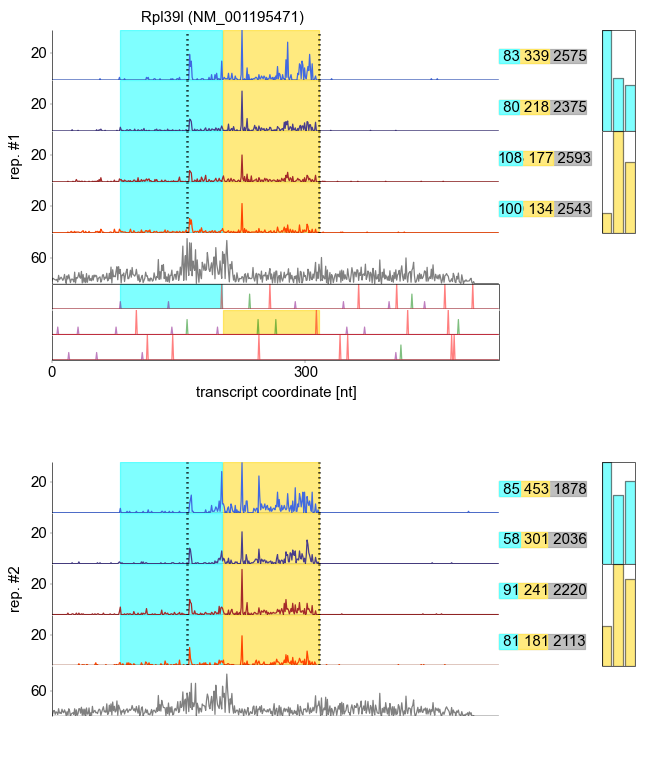

Supplement: Additional file 2: — This file contains a mini web site with additional ribosome profiles of individual mRNAs that are mentioned in the manuscript. The same mini web site is available at http://lapti.ucc.ie/ogd/. [file 13059_2015_651_MOESM2_ESM.zip › ogd/profiles/NM_001195471.png]

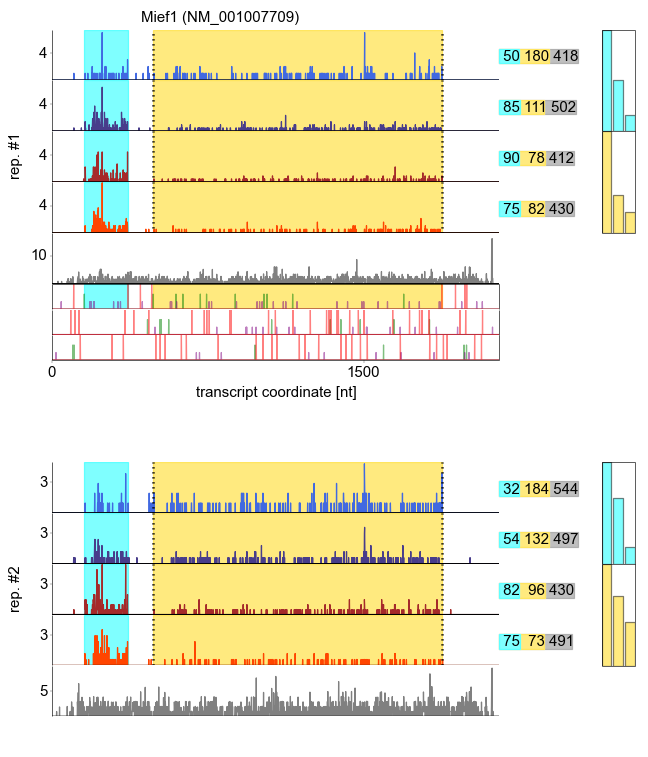

Supplement: Additional file 2: — This file contains a mini web site with additional ribosome profiles of individual mRNAs that are mentioned in the manuscript. The same mini web site is available at http://lapti.ucc.ie/ogd/. [file 13059_2015_651_MOESM2_ESM.zip › ogd/profiles/NM_001007709.png]

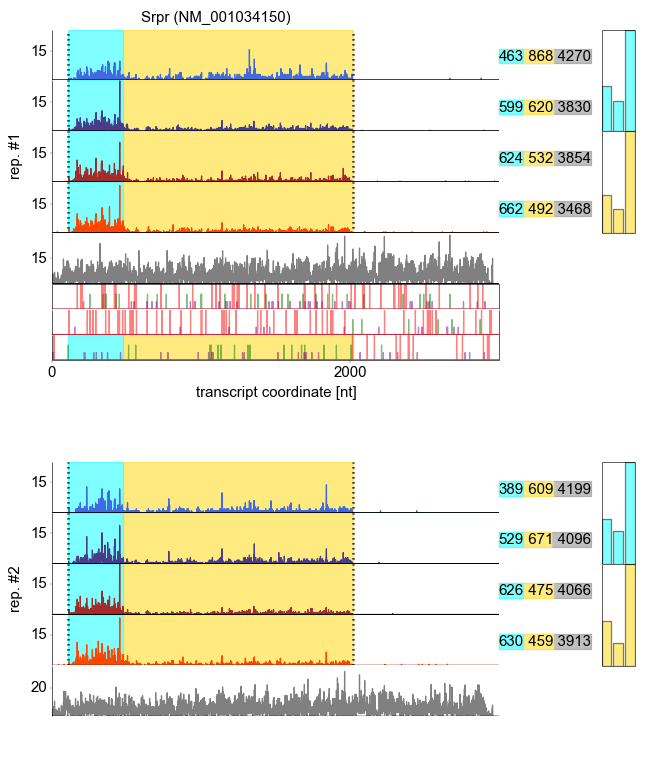

Supplement: Additional file 2: — This file contains a mini web site with additional ribosome profiles of individual mRNAs that are mentioned in the manuscript. The same mini web site is available at http://lapti.ucc.ie/ogd/. [file 13059_2015_651_MOESM2_ESM.zip › ogd/profiles/NM_001034150.png]

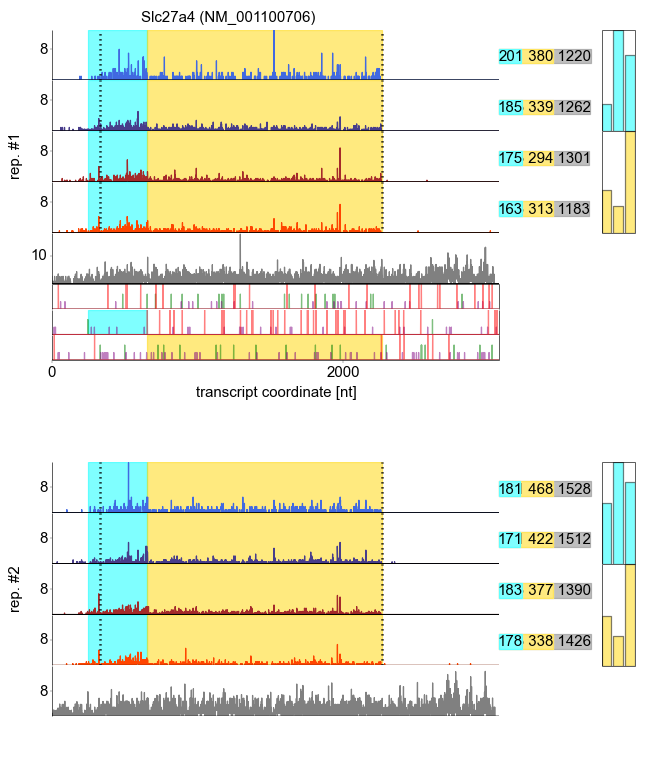

Supplement: Additional file 2: — This file contains a mini web site with additional ribosome profiles of individual mRNAs that are mentioned in the manuscript. The same mini web site is available at http://lapti.ucc.ie/ogd/. [file 13059_2015_651_MOESM2_ESM.zip › ogd/profiles/NM_001100706.png]

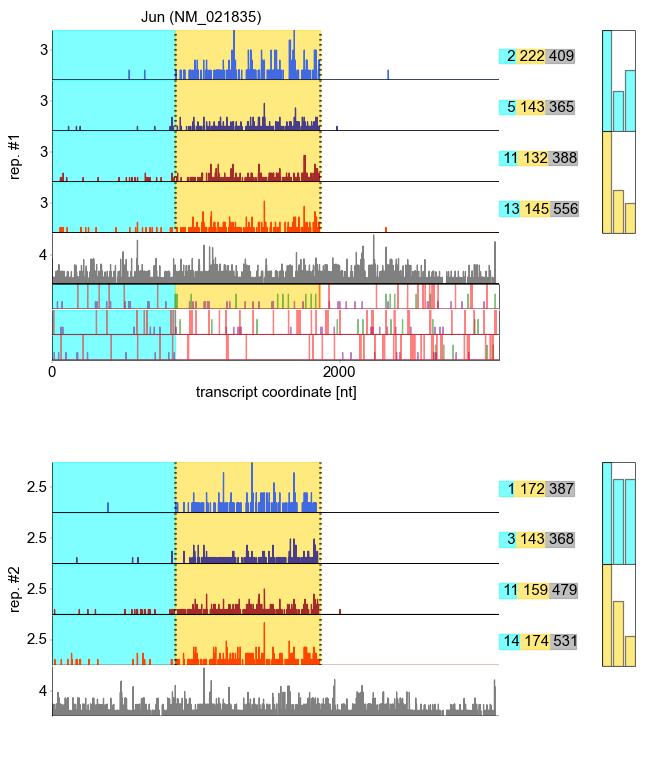

Supplement: Additional file 2: — This file contains a mini web site with additional ribosome profiles of individual mRNAs that are mentioned in the manuscript. The same mini web site is available at http://lapti.ucc.ie/ogd/. [file 13059_2015_651_MOESM2_ESM.zip › ogd/profiles/NM_021835.png]

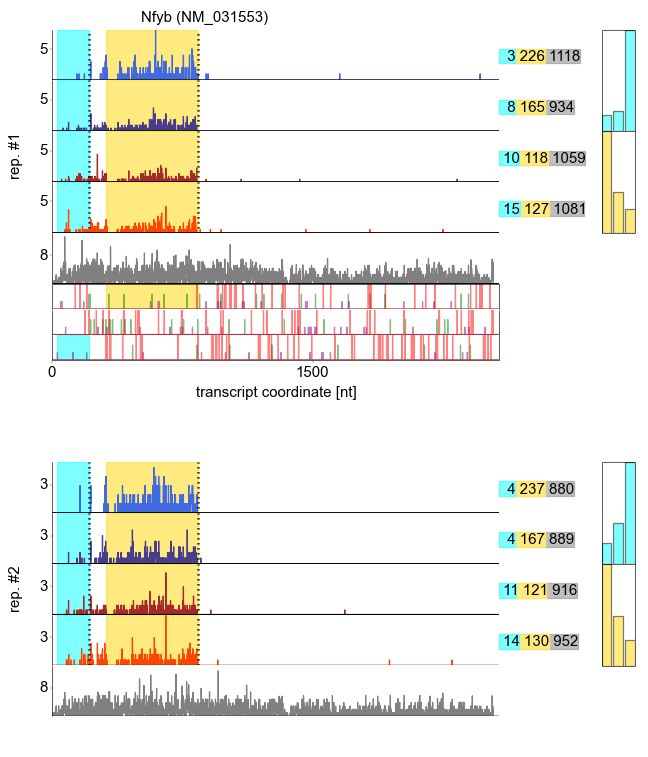

Supplement: Additional file 2: — This file contains a mini web site with additional ribosome profiles of individual mRNAs that are mentioned in the manuscript. The same mini web site is available at http://lapti.ucc.ie/ogd/. [file 13059_2015_651_MOESM2_ESM.zip › ogd/profiles/NM_031553.png]

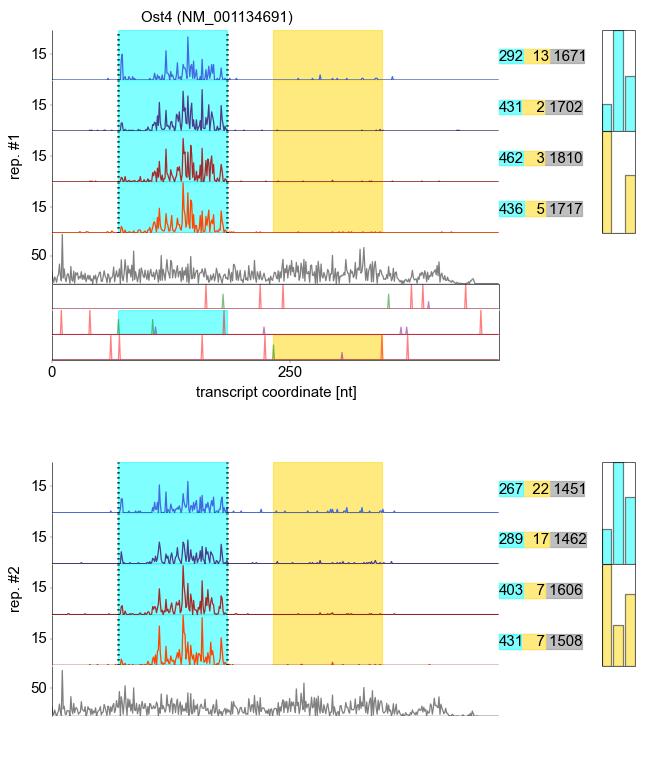

Supplement: Additional file 2: — This file contains a mini web site with additional ribosome profiles of individual mRNAs that are mentioned in the manuscript. The same mini web site is available at http://lapti.ucc.ie/ogd/. [file 13059_2015_651_MOESM2_ESM.zip › ogd/profiles/NM_001134691.png]

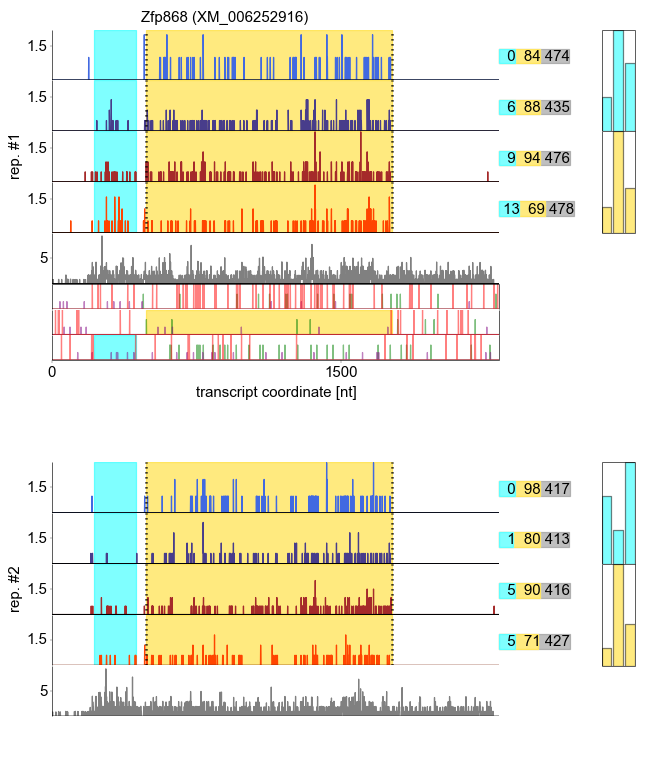

Supplement: Additional file 2: — This file contains a mini web site with additional ribosome profiles of individual mRNAs that are mentioned in the manuscript. The same mini web site is available at http://lapti.ucc.ie/ogd/. [file 13059_2015_651_MOESM2_ESM.zip › ogd/profiles/XM_006252916.png]

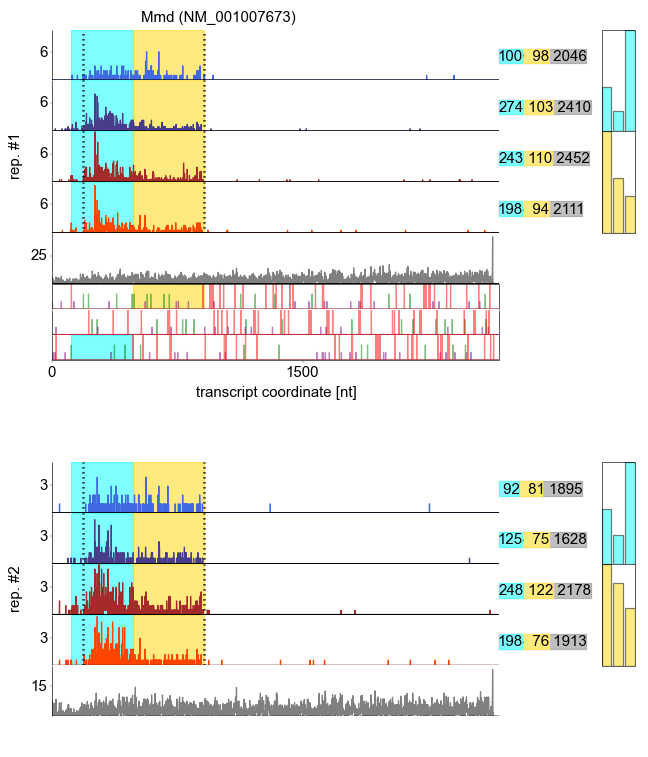

Supplement: Additional file 2: — This file contains a mini web site with additional ribosome profiles of individual mRNAs that are mentioned in the manuscript. The same mini web site is available at http://lapti.ucc.ie/ogd/. [file 13059_2015_651_MOESM2_ESM.zip › ogd/profiles/NM_001007673.png]

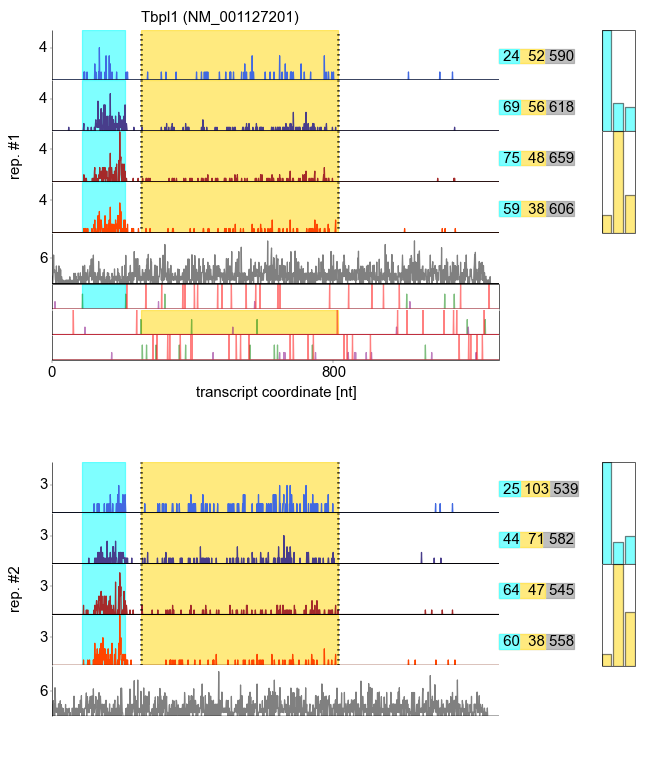

Supplement: Additional file 2: — This file contains a mini web site with additional ribosome profiles of individual mRNAs that are mentioned in the manuscript. The same mini web site is available at http://lapti.ucc.ie/ogd/. [file 13059_2015_651_MOESM2_ESM.zip › ogd/profiles/NM_001127201.png]

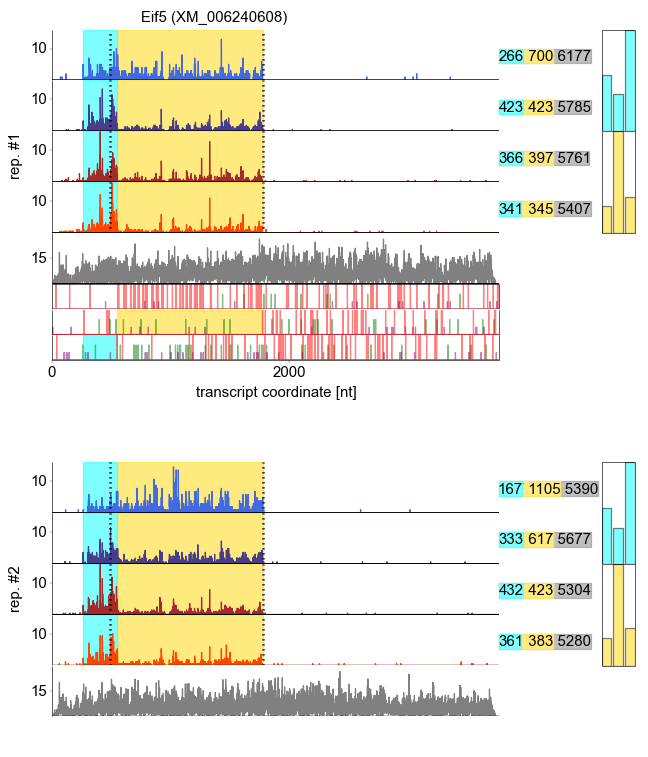

Supplement: Additional file 2: — This file contains a mini web site with additional ribosome profiles of individual mRNAs that are mentioned in the manuscript. The same mini web site is available at http://lapti.ucc.ie/ogd/. [file 13059_2015_651_MOESM2_ESM.zip › ogd/profiles/XM_006240608.png]

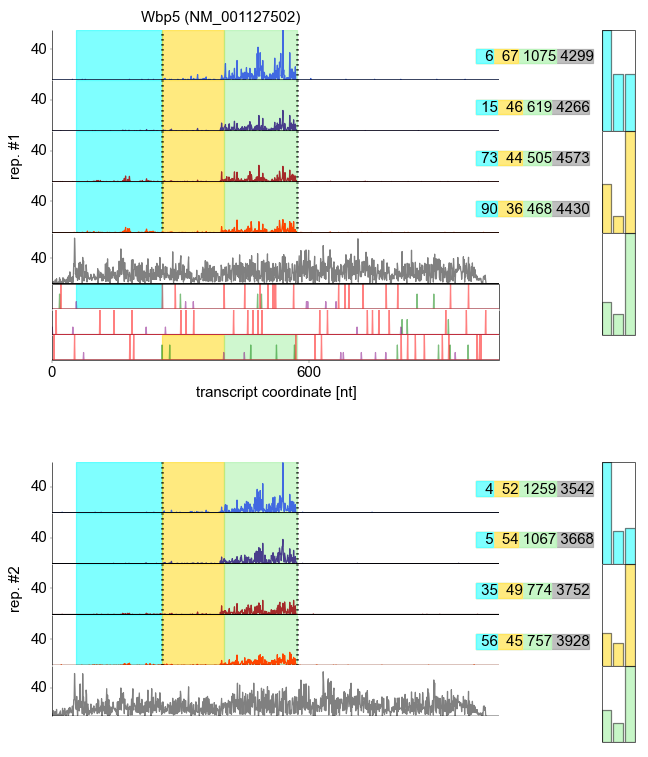

Supplement: Additional file 2: — This file contains a mini web site with additional ribosome profiles of individual mRNAs that are mentioned in the manuscript. The same mini web site is available at http://lapti.ucc.ie/ogd/. [file 13059_2015_651_MOESM2_ESM.zip › ogd/profiles/NM_001127502.png]

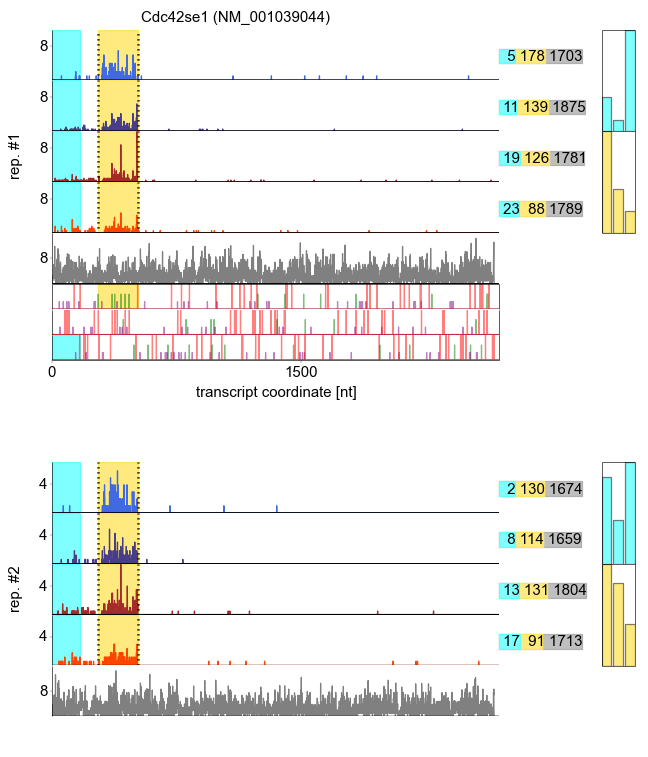

Supplement: Additional file 2: — This file contains a mini web site with additional ribosome profiles of individual mRNAs that are mentioned in the manuscript. The same mini web site is available at http://lapti.ucc.ie/ogd/. [file 13059_2015_651_MOESM2_ESM.zip › ogd/profiles/NM_001039044.png]

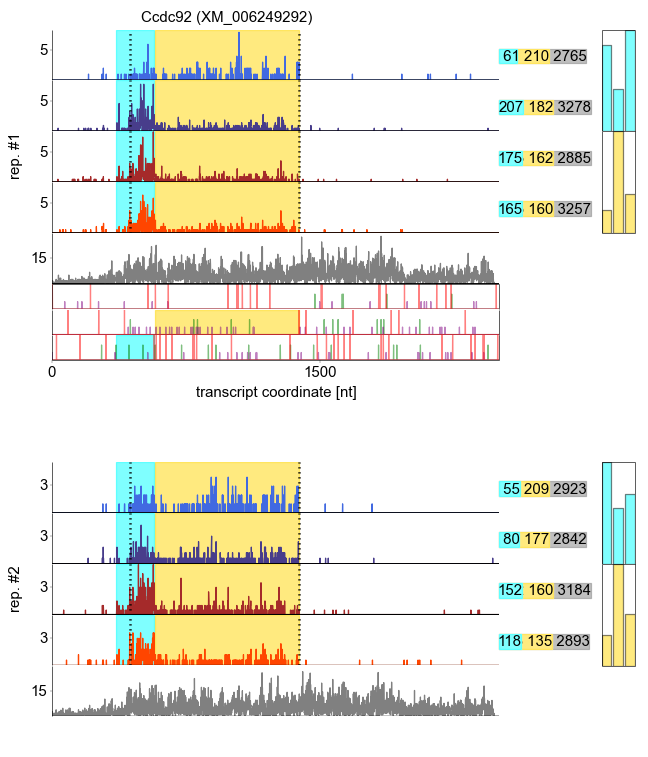

Supplement: Additional file 2: — This file contains a mini web site with additional ribosome profiles of individual mRNAs that are mentioned in the manuscript. The same mini web site is available at http://lapti.ucc.ie/ogd/. [file 13059_2015_651_MOESM2_ESM.zip › ogd/profiles/XM_006249292.png]

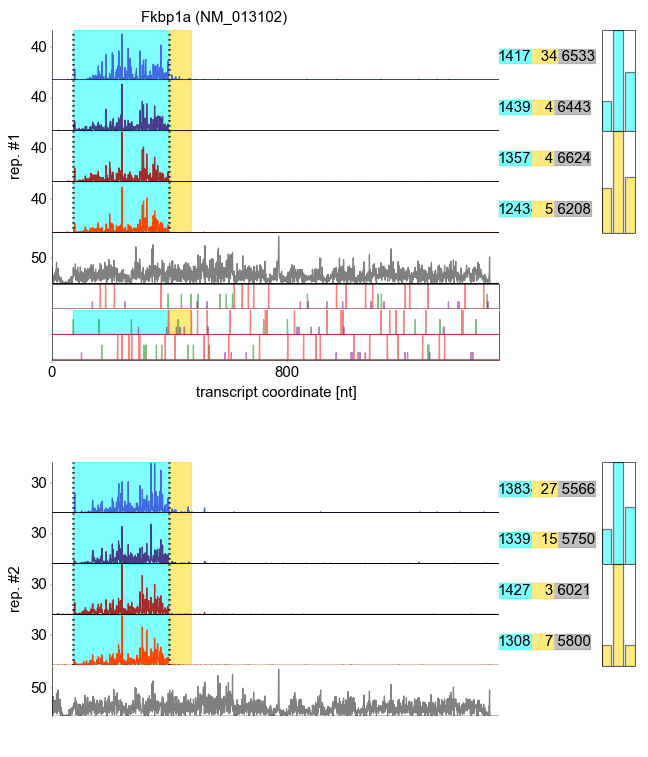

Supplement: Additional file 2: — This file contains a mini web site with additional ribosome profiles of individual mRNAs that are mentioned in the manuscript. The same mini web site is available at http://lapti.ucc.ie/ogd/. [file 13059_2015_651_MOESM2_ESM.zip › ogd/profiles/NM_013102.png]

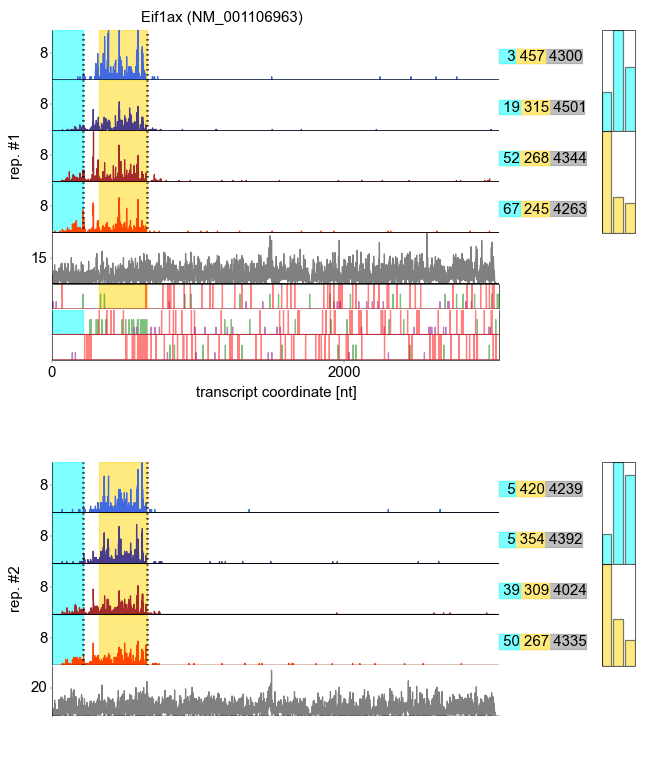

Supplement: Additional file 2: — This file contains a mini web site with additional ribosome profiles of individual mRNAs that are mentioned in the manuscript. The same mini web site is available at http://lapti.ucc.ie/ogd/. [file 13059_2015_651_MOESM2_ESM.zip › ogd/profiles/NM_001106963.png]

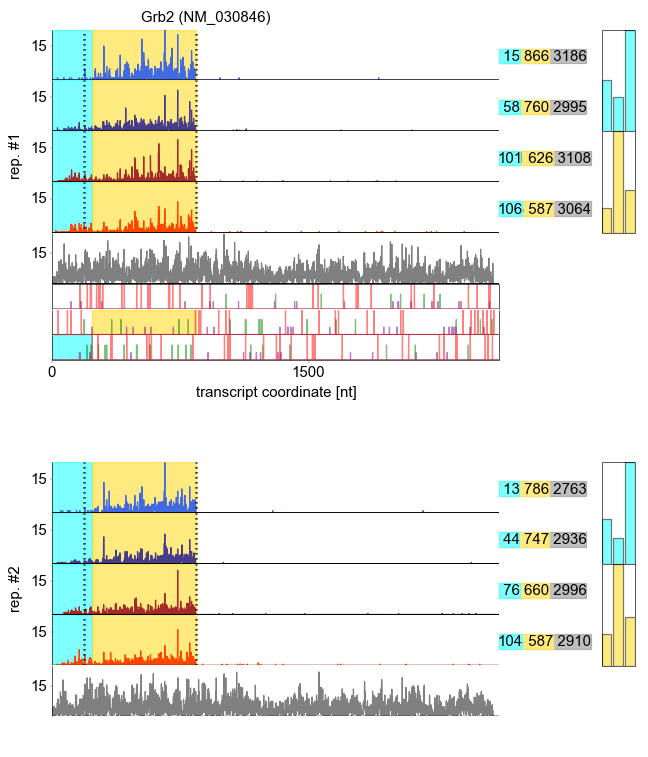

Supplement: Additional file 2: — This file contains a mini web site with additional ribosome profiles of individual mRNAs that are mentioned in the manuscript. The same mini web site is available at http://lapti.ucc.ie/ogd/. [file 13059_2015_651_MOESM2_ESM.zip › ogd/profiles/NM_030846.png]

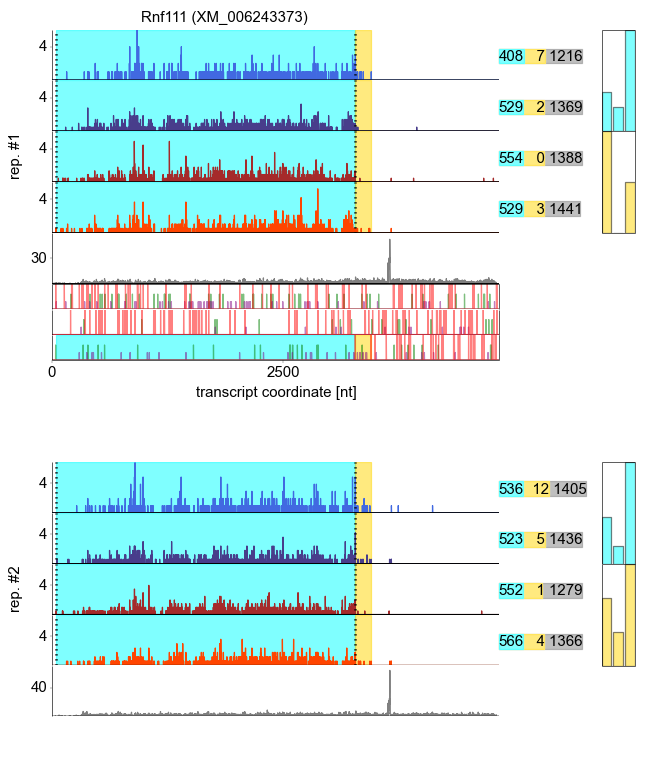

Supplement: Additional file 2: — This file contains a mini web site with additional ribosome profiles of individual mRNAs that are mentioned in the manuscript. The same mini web site is available at http://lapti.ucc.ie/ogd/. [file 13059_2015_651_MOESM2_ESM.zip › ogd/profiles/XM_006243373.png]

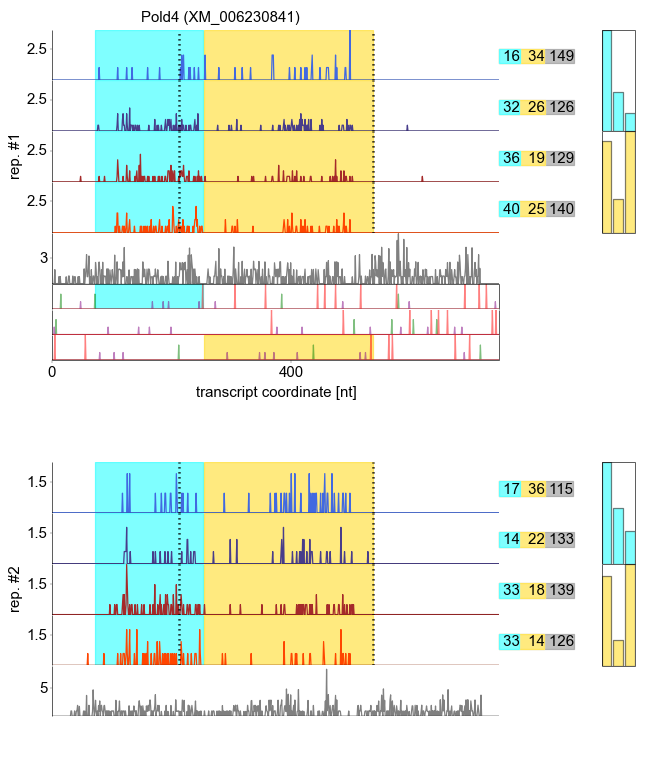

Supplement: Additional file 2: — This file contains a mini web site with additional ribosome profiles of individual mRNAs that are mentioned in the manuscript. The same mini web site is available at http://lapti.ucc.ie/ogd/. [file 13059_2015_651_MOESM2_ESM.zip › ogd/profiles/XM_006230841.png]

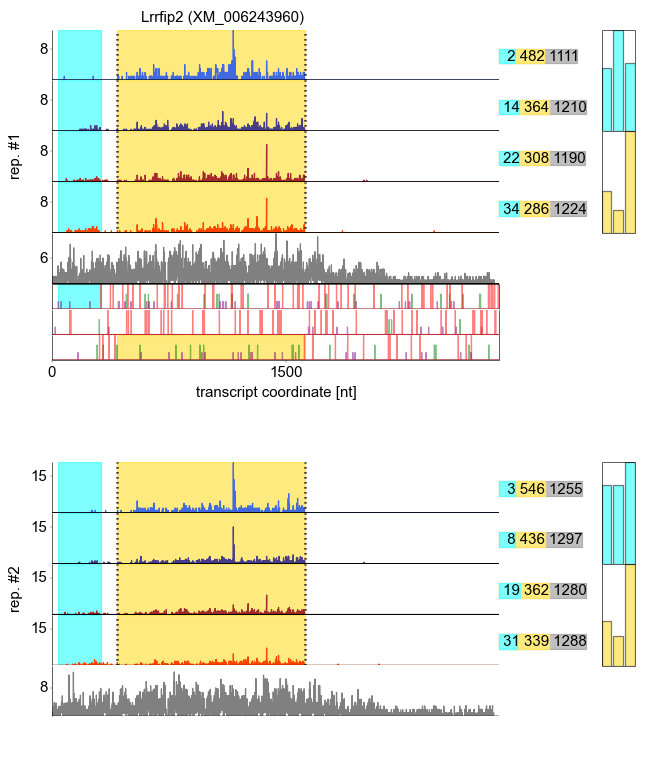

Supplement: Additional file 2: — This file contains a mini web site with additional ribosome profiles of individual mRNAs that are mentioned in the manuscript. The same mini web site is available at http://lapti.ucc.ie/ogd/. [file 13059_2015_651_MOESM2_ESM.zip › ogd/profiles/XM_006243960.png]

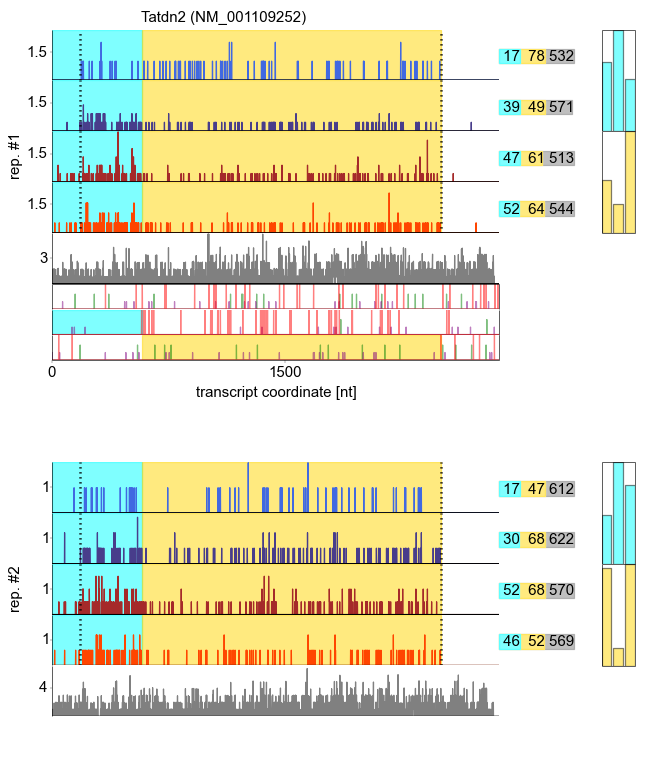

Supplement: Additional file 2: — This file contains a mini web site with additional ribosome profiles of individual mRNAs that are mentioned in the manuscript. The same mini web site is available at http://lapti.ucc.ie/ogd/. [file 13059_2015_651_MOESM2_ESM.zip › ogd/profiles/NM_001109252.png]

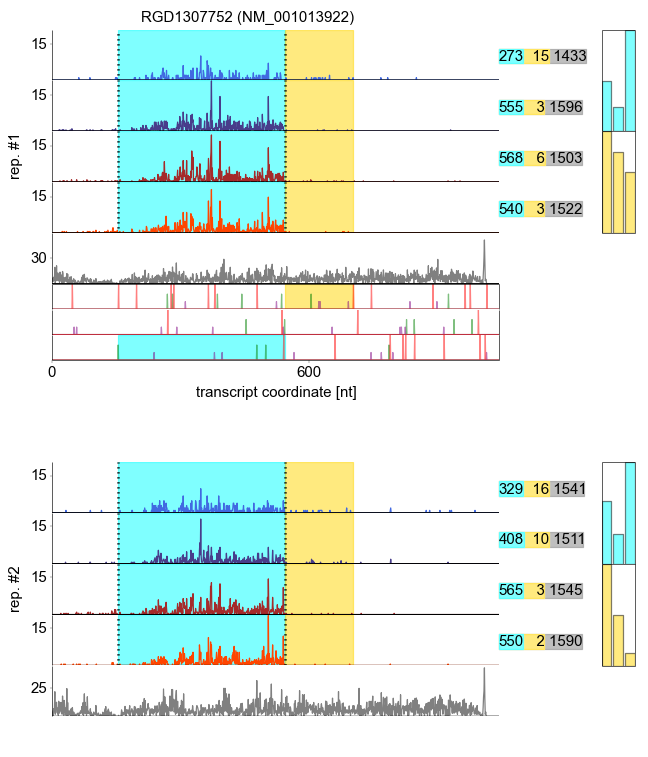

Supplement: Additional file 2: — This file contains a mini web site with additional ribosome profiles of individual mRNAs that are mentioned in the manuscript. The same mini web site is available at http://lapti.ucc.ie/ogd/. [file 13059_2015_651_MOESM2_ESM.zip › ogd/profiles/NM_001013922.png]

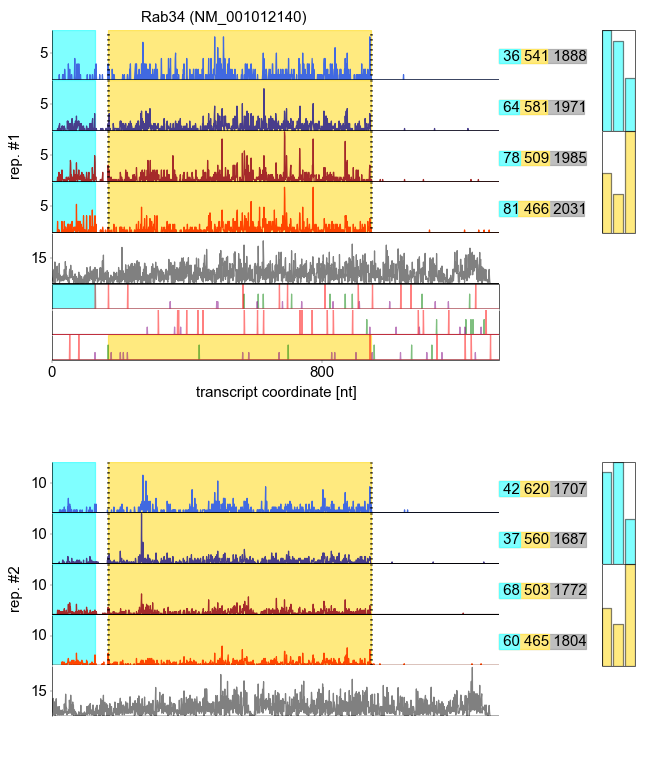

Supplement: Additional file 2: — This file contains a mini web site with additional ribosome profiles of individual mRNAs that are mentioned in the manuscript. The same mini web site is available at http://lapti.ucc.ie/ogd/. [file 13059_2015_651_MOESM2_ESM.zip › ogd/profiles/NM_001012140.png]

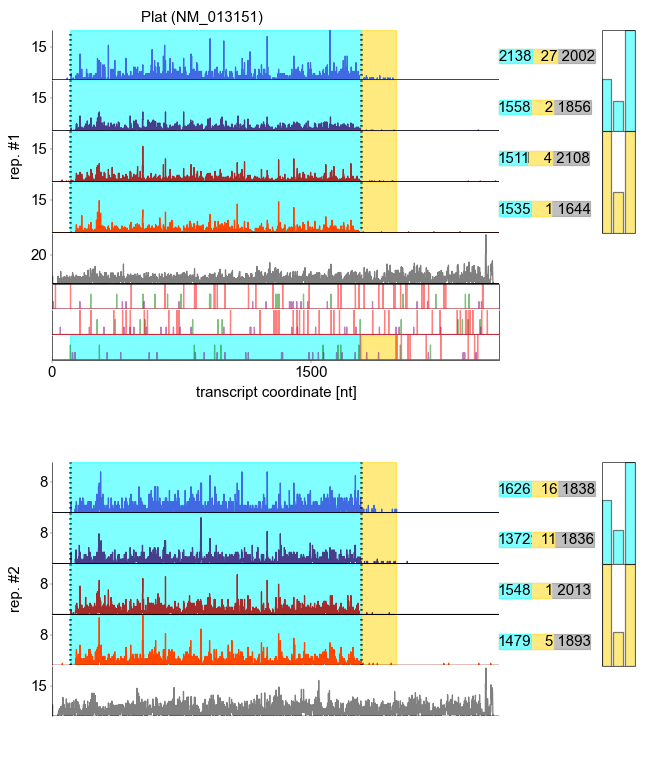

Supplement: Additional file 2: — This file contains a mini web site with additional ribosome profiles of individual mRNAs that are mentioned in the manuscript. The same mini web site is available at http://lapti.ucc.ie/ogd/. [file 13059_2015_651_MOESM2_ESM.zip › ogd/profiles/NM_013151.png]

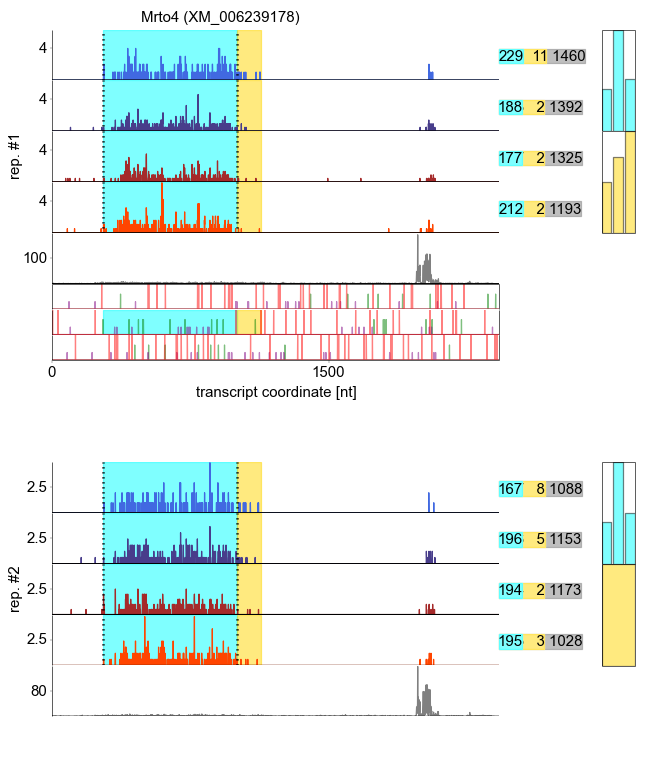

Supplement: Additional file 2: — This file contains a mini web site with additional ribosome profiles of individual mRNAs that are mentioned in the manuscript. The same mini web site is available at http://lapti.ucc.ie/ogd/. [file 13059_2015_651_MOESM2_ESM.zip › ogd/profiles/XM_006239178.png]

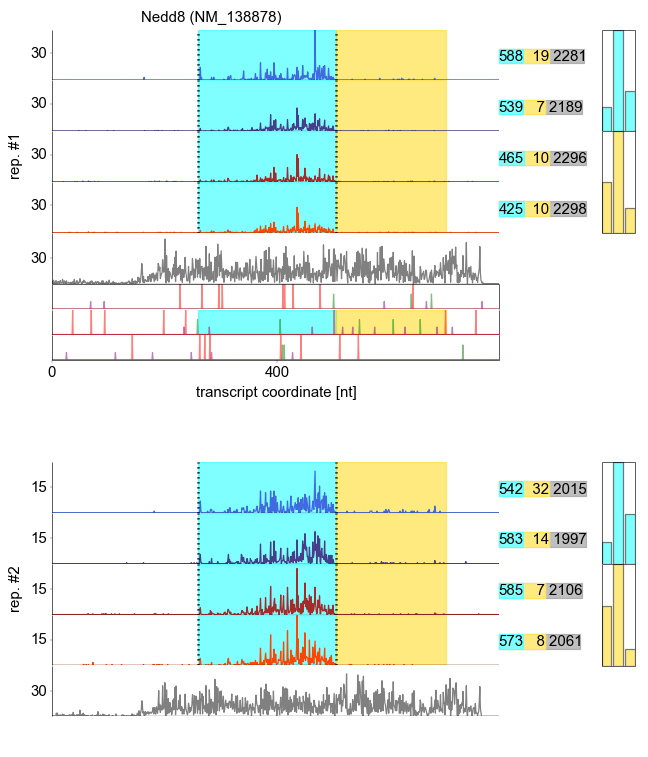

Supplement: Additional file 2: — This file contains a mini web site with additional ribosome profiles of individual mRNAs that are mentioned in the manuscript. The same mini web site is available at http://lapti.ucc.ie/ogd/. [file 13059_2015_651_MOESM2_ESM.zip › ogd/profiles/NM_138878.png]

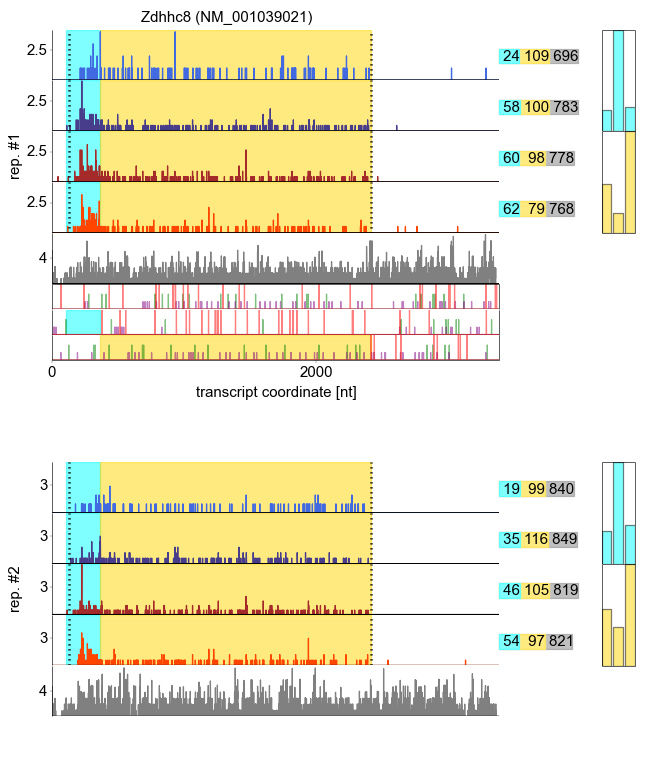

Supplement: Additional file 2: — This file contains a mini web site with additional ribosome profiles of individual mRNAs that are mentioned in the manuscript. The same mini web site is available at http://lapti.ucc.ie/ogd/. [file 13059_2015_651_MOESM2_ESM.zip › ogd/profiles/NM_001039021.png]

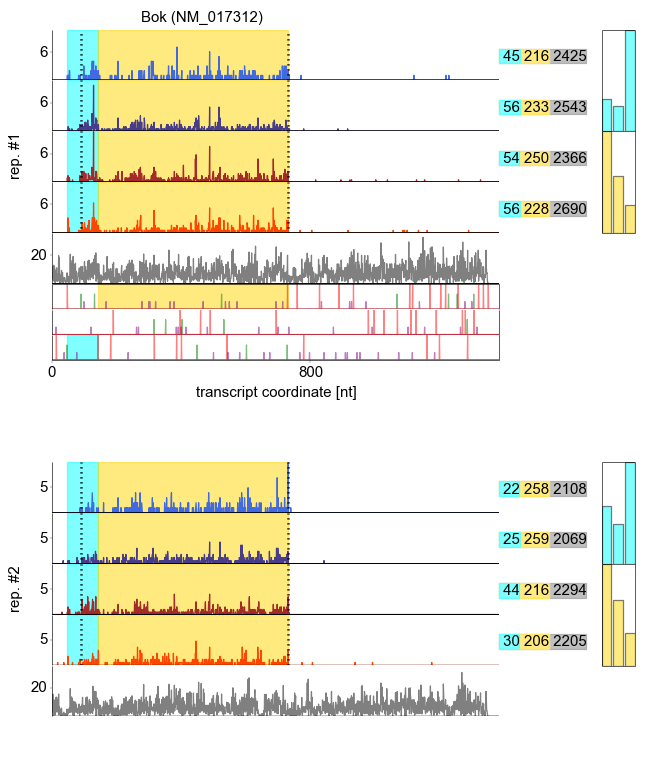

Supplement: Additional file 2: — This file contains a mini web site with additional ribosome profiles of individual mRNAs that are mentioned in the manuscript. The same mini web site is available at http://lapti.ucc.ie/ogd/. [file 13059_2015_651_MOESM2_ESM.zip › ogd/profiles/NM_017312.png]

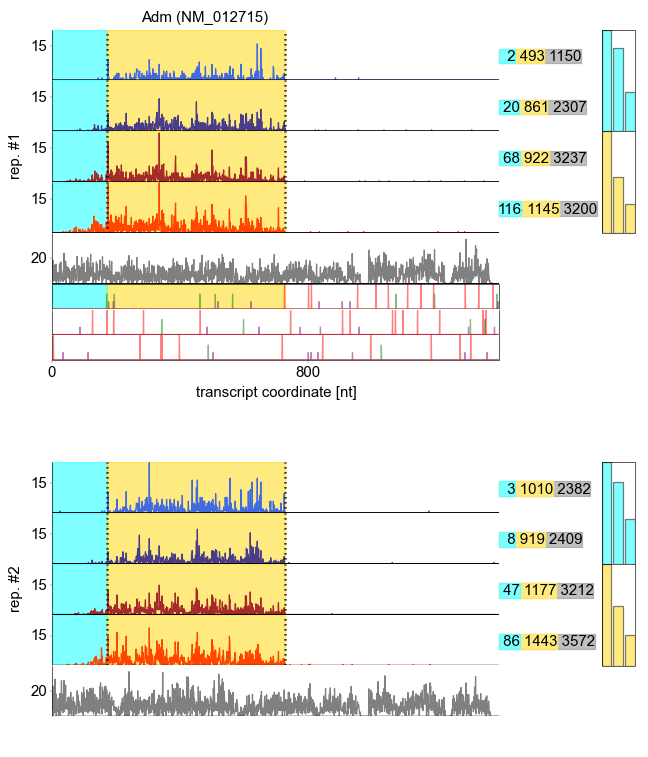

Supplement: Additional file 2: — This file contains a mini web site with additional ribosome profiles of individual mRNAs that are mentioned in the manuscript. The same mini web site is available at http://lapti.ucc.ie/ogd/. [file 13059_2015_651_MOESM2_ESM.zip › ogd/profiles/NM_012715.png]
