# Supplementary material for: Oxygen and glucose deprivation induces widespread alterations in mRNA translation within 20 minutes
Source: Genome Biol. 2015 May 6;16(1):90. doi: 10.1186/s13059-015-0651-z (PMC4419486; doi:10.1186/s13059-015-0651-z)
Supplement: Additional file 4: — Additional Table. Primary and secondary antibodies used. [file 13059_2015_651_MOESM4_ESM.pdf]

Additional Table. Primary and secondary antibodies used.

Company	Name	Cat N
	Primary antibodies	
Cell Signalling Technology, MA	Adenosine monophosphate activated protein kinase alpha (AMPKα)	2532
	phospho-AMPK (Thr172)	2535
	Mammalian target of rapamycin (mTOR)	2972
	phospho-mTOR (Ser2448)	2971
	phospho-Akt (Ser473)	4060
	Hexokinase II	2867
	Lactate dehydrogenase A (LDHA)	2012
	phospho-p44/42 MAPK (Erk1/2) (Thr202/Tyr204)	9101
	Aconitase 2 (ACO2)	6571
	Ubiquitin-Conjugating Enzyme E2S (UBE2S)	9630
	Eukaryotic elongation factor-2 kinase (eEF2k)	3692
	p-eEF2 (Thr56)	2331
	S6 Ribosomal Protein	2217
	phospho-S6 Ribosomal Protein (ser235/236)	2211
	Acetyl-CoA carboxylase (ACC)	3662
	phospho-ACC (Ser79)	3661
Millipore, CA	Akt / Protein Kinase B (PKB)	O7416
	Mitogen-activated protein kinase, p44/42 MAPK (Erk1/2)	06-182
	Anti-Uncoupling Protein-2 (UCP-2)	662047-100
R&D Systems, MN	Hypoxia inducible factor 1α (HIF-1 α)	MAB1536
	Hypoxia inducible factor 2α (HIF-2 α)	AF2886
Abcam, UK	Iron-sulfur cluster assembly enzyme (ISCU)	ab113812
	Jun B proto-oncogene (JunB)	ab31421
	Parathymosin (PTMS)	Ab172274
Acris, LA	Cytochrome oxidase subunit IV (COX IV) isoform 1	AM06506SU-N
Santa Cruz Biotech	Voltage-dependent anion channel 1 (VDAC1)	sc-8828
Proteintech, IL	ATP Synthase, H^+ Transporting, Mitochondrial F1 Complex, Alpha Subunit 1 (ATP5A1)	14676-1-AP
Sigma, MO	α -tubulin	T5168
	Secondary antibodies	
Sigma, MO	Mouse anti-goat/sheep IgG-peroxidase	A9452
	Mouse anti-rabbit IgG-peroxidase	A1949
	Goat anti-mouse IgG-peroxidase	A0168
